# Flipping the Switch: MeCP2‐Mediated Lactylation Rewires Microglial Metabolism and Inflammation via the HK2/mTOR Axis in Poststroke Neuroinflammation

**DOI:** 10.1002/advs.202513400

**Published:** 2025-12-22

**Authors:** Zengyu Zhang, Shanshan Huang, Yong Wang, Zhiwen Jiang, Zhuohang Liu, Chenran Wang, Rong Ji, Yiwen Yuan, Xueyu Mao, Kaicheng Yang, Huicong Niu, Yanqin Gao, Jing Zhao

**Affiliations:** ^1^ Department of Neurology of Minhang Hospital State Key Laboratory of Brain Function and Disorders MOE Frontiers Center for Brain Science, and Institutes of Brain Science Fudan University Shanghai 200032 China; ^2^ Department of Endocrinology Affiliated Hospital of Jiangnan University Jiangnan University Wuxi 214122 China; ^3^ Department of Neurology Zhongshan Hospital Fudan University Shanghai 200032 China; ^4^ Department of Neurosurgery of Huashan Hospital State Key Laboratory of Brain Function and Disorders MOE Frontiers Center for Brain Science and Institutes of Brain Science Fudan University Shanghai 200032 China; ^5^ Institute of Healthy Yangtze River Delta Shanghai Jiao Tong University Shanghai 200030 China

**Keywords:** MeCP2, ischemic stroke, lactylation, microglia, neuroinflammation

## Abstract

Microglial metabolic/inflammatory reprogramming critically influences stroke outcomes, yet its mechanisms remain poorly understood. Lysine lactylation, an epigenetic modification in which lactate‐derived lactyl groups modify lysine residues, regulates immune and neurological processes. Here, lysine lactylation is identified as a key link between ischemic metabolic stress and microglial dysfunction. Stroke‐induced lactate accumulation drives microglial protein lactylation, which correlates with poor neurological outcomes. Proteomics identified that methyl‐CpG binding protein 2 (MeCP2) is lactylated at lysine 210 (K210), enhancing its transcriptional activation of glycolytic/inflammatory genes, especially hexokinase 2 (HK2). HK2 overexpression mimics lactylation‐induced pathology (mitochondrial dysfunction, glycolytic shift, inflammation), while knockdown reverses these effects. Lactylated MeCP2 impairs mitochondrial respiration, disrupts metabolic signaling (leading to dysregulated activation of the mammalian target of rapamycin (mTOR)/AMPK pathway), and sustains neuroinflammation. Genetic ablation of MeCP2‐K210 lactylation (via K210R mutation), pharmacological inhibition of lactyltransferase p300, or HK2 inhibition with lonidamine restores mitochondrial function, attenuates neuroinflammation, and improves neurofunctional recovery. The findings establish MeCP2‐K210 lactylation as a critical metabolic‐epigenetic switch driving microglial activation via the HK2/mTOR axis, identifying a therapeutic target for postischemic neuroinflammation.

## Graphical abstract

Stroke‐induced lactate accumulation promotes p300‐mediated lactylation of methyl‐CpG binding protein 2 (MeCP2) at lysine 210, which reprograms microglial metabolism toward glycolysis and activates the hexokinase 2 (HK2)/mTOR axis. This cascade promotes proinflammatory responses and impairs neurofunctional outcomes. In contrast, targeting this lactylation‐metabolic axis—through HK2 inhibition, p300 blockade, or ablation of MeCP2 K210 lactylation—restores metabolic homeostasis, attenuates neuroinflammation, and enhances poststroke recovery.

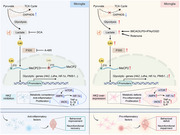

## 1 Introduction

Ischemic stroke remains a major cause of death and long‐term disability worldwide, driven not only by acute vascular occlusion but also by secondary cellular responses that exacerbate brain injury.^[^ ^1^ ^]^ Among these, microglia—the resident immune cells of the central nervous system—play a dual role, capable of both driving neuroinflammation and facilitating repair.^[^ ^2^, ^3^ ^]^ In the early phases of stroke, microglia rapidly shift toward proinflammatory phenotypes, secreting cytokines, amplifying oxidative stress, and aggravating neuronal death.^[^ ^4^, ^5^, ^6^ ^]^ This maladaptive activation is increasingly linked to metabolic dysfunction, yet the upstream molecular signals coupling metabolic changes to microglial transcriptional reprogramming remain incompletely understood.^[^ ^7^ ^]^

A hallmark of cerebral ischemia is the accumulation of lactate, a byproduct of enhanced glycolytic flux. Although previously viewed as a metabolic waste, lactate is now recognized as both an alternative energy substrate and a signaling molecule.^[^ ^8^, ^9^, ^10^ ^]^ In experimental models of stroke, exogenous lactate has been shown to improve outcomes, partially through its role in neuronal energetics.^[^ ^11^ ^]^ Beyond bioenergetics, lactate modulates gene expression by serving as a substrate for lysine lactylation (Kla), a recently discovered post‐translational modification that affects histone and nonhistone proteins.^[^ ^12^, ^13^ ^]^ Kla has emerged as a crucial interface between metabolism and epigenetic control, particularly under hypoxic or inflammatory stress. Recent studies have begun to explore lactylation in stroke: one identified methyl‐CpG binding protein 2 (MeCP2) lactylation as a protective mechanism against neuronal apoptosis via repressing proapoptotic genes,^[^ ^14^ ^]^ another demonstrated that microglial SMEK1 regulates H3K9 lactylation to modulate glycolysis and neuroinflammation postischemia,^[^ ^15^ ^]^ and a third showed that inhibiting glycolysis reduces LCP1 lactylation to alleviate cerebral infarction.^[^ ^16^ ^]^ Despite these advances, the physiological relevance of lactylation in shaping microglial functional states and its potential as a target for microglia‐specific therapies remain largely unexplored.

Growing evidence suggests that lactylation may fine‐tune immune cell phenotypes by coordinating metabolic status with transcriptional output.^[^ ^17^ ^]^ In macrophages, lactate‐induced Kla drives a transition from inflammatory to reparative states via selective activation of genes, such as *Arg1* and *Vegfa*.^[^ ^18^, ^19^, ^20^ ^]^ Given the shared ontogeny and plasticity of microglia and macrophages, it is plausible that similar lactate‐driven epigenetic mechanisms exist in microglia. Moreover, microglial heterogeneity in stroke—spanning proinflammatory, anti‐inflammatory, and proliferative states—is thought to underpin divergent effects on neurodegeneration and recovery.^[^ ^21^, ^22^ ^]^ Yet, whether lactylation participates in shaping these functional states in ischemic microglia remains unknown. Elucidating this mechanism could reveal how metabolic stressors are transduced into pathogenic transcriptional programs during stroke.

In this study, we identified lysine lactylation as a critical regulator of microglial activation following ischemic stroke. Using integrated in vivo and in vitro models, we demonstrated that stroke‐induced lactate accumulation drove selective protein lactylation in microglia, with proteomic profiling revealing MeCP2 as a major lactylated substrate.^[^ ^23^ ^]^ Lactylation at lysine 210 enhanced MeCP2's transcriptional activity at glycolytic and proinflammatory gene loci, notably hexokinase 2 (HK2), a central glycolytic checkpoint. This modification promoted mitochondrial dysfunction and disrupted mammalian target of rapamycin (mTOR)–AMPK signaling, sustaining microglial inflammatory activation and pathological proliferation. Importantly, we extend prior lactylation‐focused stroke research by showing that microglia‐specific targeting restores these deficits: this includes genetic disruption of MeCP2 K210 lactylation, pharmacological inhibition of its lactylation writer p300, or modulation via metabolic/mitochondrial agents (DCA, a metabolic modulator mitigating glycolytic flux and downstream lactylation; ROT, which targets microglial mitochondrial dysfunction linked to MeCP2‐driven reprogramming). Additionally, selective inhibition of HK2 using lonidamine (LND) mitigated neuroinflammation and enhanced long‐term functional outcomes in stroke models. These findings establish MeCP2 lactylation as a critical metabolic–epigenetic switch driving poststroke microglial dysfunction and identify the MeCP2–HK2 axis as a viable target for immunometabolic therapy in ischemic brain injury.

## 2 Results

### 2.1 Ischemic Stroke Drives Lactate Accumulation and Microglial Lactylation via Metabolic and Inflammatory Reprogramming

**Figure 1:**
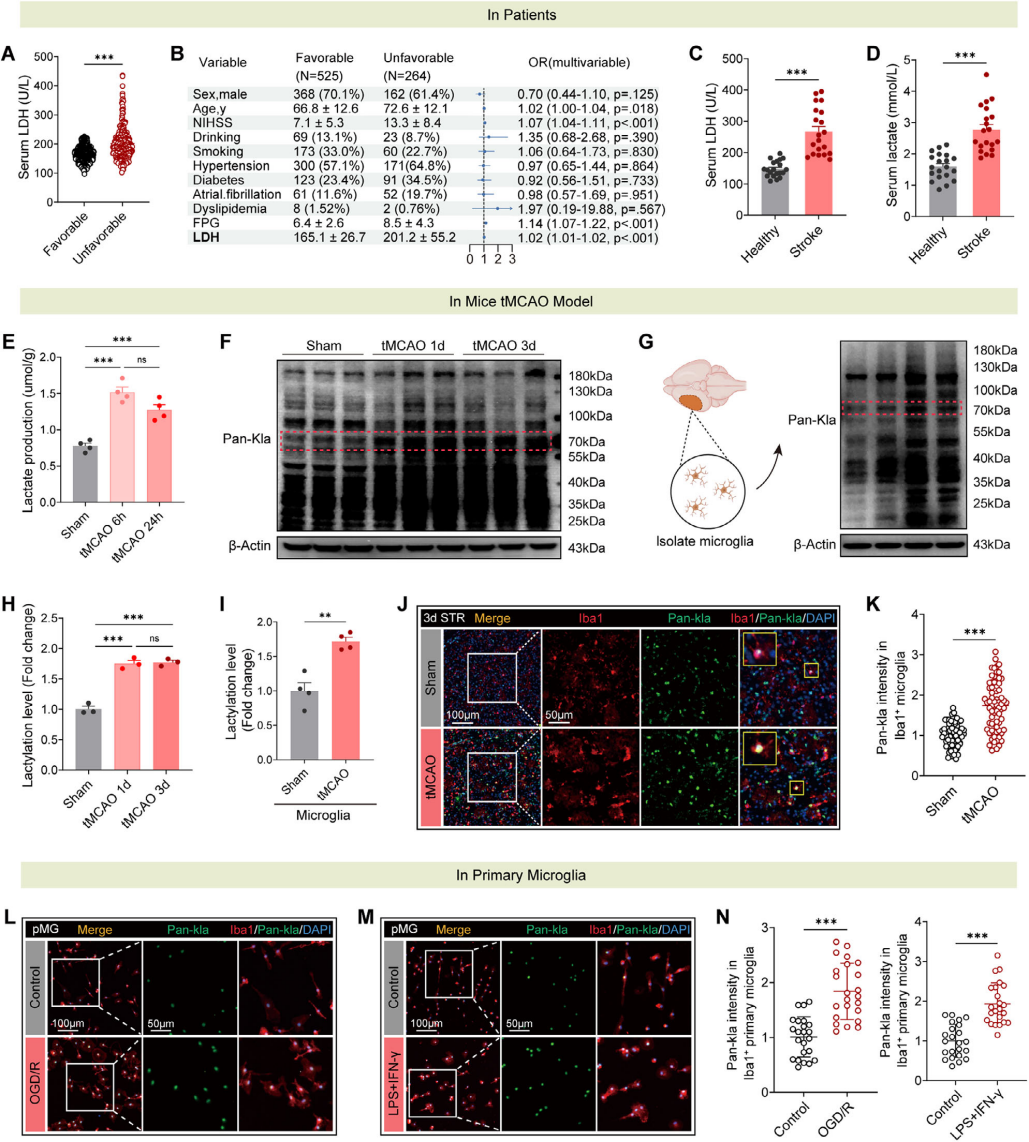
Increased lactate accumulation and protein lactylation correlate with microglial activation in ischemic stroke. A) Serum LDH levels in patients with favorable (*n* = 525) and unfavorable (*n* = 264) outcomes. B) Multivariable analysis of clinical variables associated with stroke prognosis, including LDH levels. OR, odds ratio; FPG, fasting plasma glucose. C,D) Comparison of serum LDH C) and lactate D) levels between healthy individuals and stroke patients. *n* = 20 per group. E) Lactate production in the brain tissue of sham‐operated and tMCAO mice. *n* = 4 per group. F,H) Western blot analysis of Pan‐Kla expression in brain tissue from sham, 1‐day tMCAO, and 3‐day tMCAO mice. Quantification of Pan‐Kla levels is performed. *n* = 3 per group. G,I) Western blot analysis of Pan‐Kla expression in isolated microglia from sham and tMCAO mice. Quantification is performed. *n* = 4 per group. J,K) Immunofluorescence staining of Pan‐Kla and Iba1 in the peri‐infarct striatum from sham and tMCAO mice (3 days post‐tMCAO). Quantification of Pan‐Kla intensity in Iba1^+^ microglia is conducted (*n* > 50 cells per group). L–N) Immunofluorescence staining of Pan‐Kla and Iba1 in primary microglia (pMG) under different conditions (*n* > 20 cells per group). OGD/R, oxygen‐glucose deprivation/reoxygenation. Data from animal experiments are means ± SEM; those from cell line experiments are means ± SD. Statistical analysis was performed using *t*‐test A,C,D,I,K,N), or one‐way ANOVA with Tukey's post hoc test E,H). ns = not significant, **p < 0.01, ***p < 0.001.

Ischemic stroke triggers a cascade of metabolic and inflammatory reprogramming, with lactate accumulation emerging as a central driver of microglial dysfunction. To investigate the underlying mechanisms, we first analyzed clinical data from a cohort of 789 ischemic stroke patients (Tables S1 and S2, Supporting Information). Notably, patients with poor functional outcomes (modified Rankin Scale [mRS] ≥3) exhibited significantly elevated serum lactate dehydrogenase (LDH) levels compared to those with favorable outcomes (mRS ≤2) (**Figure** **1**A). Multivariate logistic regression further confirmed serum LDH as an independent predictor of adverse outcomes (Figure 1B), while a separate cohort revealed systemic increases in lactate and LDH levels in stroke patients versus healthy controls (Figure 1C,D), indicating dysregulated lactate metabolism in stroke pathophysiology.

In a mouse model of transient middle cerebral artery occlusion (tMCAO), we observed marked lactate accumulation in the ipsilateral hemisphere relative to sham controls (Figure 1E), accompanied by time‐dependent increases in global protein lactylation (Pan‐Kla) in ischemic brain tissue, with prominent enrichment in the 70‐kDa protein range (Figure 1F,H). Isolation of CD11b⁺ microglia revealed robust upregulation of Pan‐Kla in these cells post‐tMCAO (Figure 1G,I; and Figure S1A, Supporting Information), confirmed by immunofluorescence co‐labeling showing preferential lactylation in microglia rather than neurons or astrocytes (Figure 1J,K; and Figure S1B–D, Supporting Information). To directly evaluate microglial lactate uptake (a key missing link between extracellular lactate accumulation and intracellular lactylation), we performed an in vitro assay using FITC‐labeled lactic acid. Primary mouse microglia were incubated with FITC‐lactate for the indicated time points, and intracellular fluorescence intensity was quantified via immunofluorescence microscopy at 30, 60, and 120 min. As shown in Figure S1E (Supporting Information), intracellular FITC‐lactate fluorescence signal increased in a time‐dependent manner—with significant elevation at 60 and 120 min relative to 30 min—demonstrating that microglia actively take up extracellular lactate. Subsequently, primary microglia exposed to oxygen‐glucose deprivation/reoxygenation (OGD/R) or proinflammatory stimuli (LPS/IFN‐γ) exhibited enhanced Pan‐Kla expression (Figure 1L–N), linking both metabolic stress and inflammatory activation to lactylation. Overall, these findings establish that ischemic stroke induces lactate‐driven lactylation in microglia, with systemic LDH levels correlating with functional impairment.

### 2.2 Pharmacological Modulation of Protein Lactylation Orchestrates Poststroke Metabolic Homeostasis and Functional Rehabilitation

**Figure 2:**
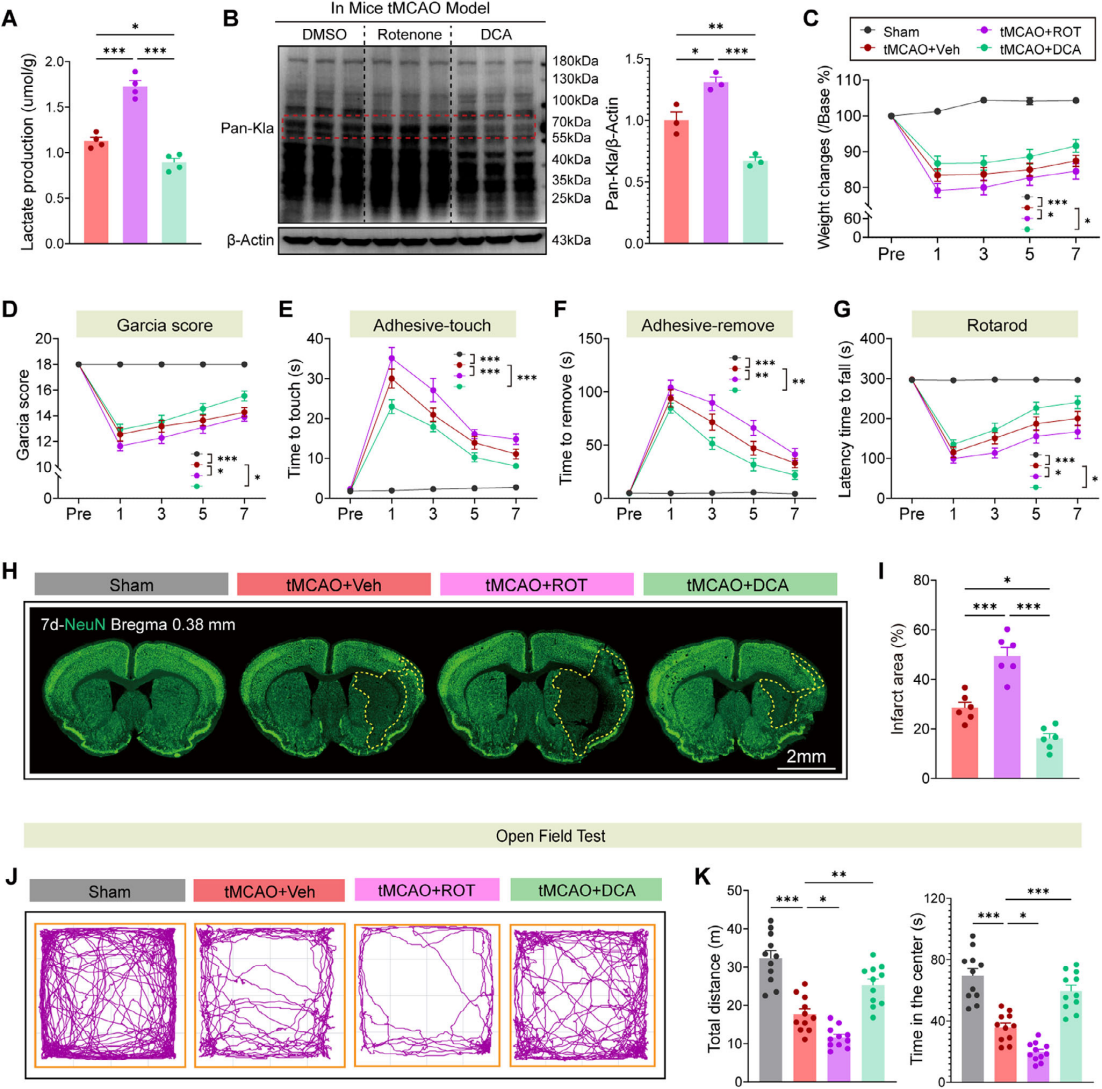
Intervention in lactylation modulates metabolism, neurological function, and neuronal apoptosis following ischemic brain injury. A) Lactate production in tMCAO mice treated with Rotenone (ROT) or Dichloroacetate (DCA). *n* = 4 per group. B) Western blot analysis of Pan‐Kla in ischemic brain tissue. *n* = 3 per group. C) Body weight changes in tMCAO mice treated with vehicle, ROT, or DCA over 7 days. D) Garcia score comparison for sensory and motor function among treatment groups. E,F) Adhesive‐touch and adhesive‐removal tests assessing sensory and fine motor function. G) Rotarod test evaluating motor coordination and endurance. *n* = 11 per group. H,I) Representative coronal brain sections (7 days post‐tMCAO) showing infarct area and corresponding quantification. *n* = 6 per group. J) Representative open field test tracking plots from sham, tMCAO, tMCAO+ROT, and tMCAO+DCA groups poststroke. K) Quantification of locomotor activity and anxiety‐like behavior in the test. Left: Total distance traveled (meters), reflecting general motor activity. Right: Time spent in the central zone, indicative of anxiety‐like behavior. *n* = 11 per group. Data are presented as mean ± SEM. Statistical analysis was performed using one‐way A,B,I,K) or two‐way ANOVA C–G) with Tukey's post hoc test. *p < 0.05, **p < 0.01, ***p < 0.001.

Building on the established role of lactate‐driven lactylation in ischemic brain injury, we investigated whether pharmacologically manipulating protein lactylation could modulate poststroke metabolic stress and neurorehabilitation. Using rotenone (ROT, a mitochondrial complex I inhibitor that promotes lactate accumulation) and dichloroacetate (DCA, a pyruvate dehydrogenase activator that enhances lactate clearance), we probed the causal link between lactylation and stroke outcomes. In sham‐operated mice, neither ROT nor DCA altered basal Pan‐Kla levels (Figure S2A, Supporting Information), confirming that lactylation remains unperturbed under physiological conditions. Following tMCAO, ROT administration exacerbated lactate accumulation and pan‐protein lactylation in the ischemic hemisphere, whereas DCA treatment suppressed both parameters (**Figure** **2**A,B), establishing that pharmacological modulation directly influences lactylation dynamics. Behaviorally, ROT‐treated mice exhibited delayed body weight recovery and deteriorated neurological function, as evidenced by lower Garcia scores, prolonged sensorimotor response latencies, and impaired rotarod performance (Figure 2C–G). Conversely, DCA intervention accelerated functional recovery, improving all behavioral metrics. Histopathological analysis revealed that ROT exacerbated infarct volume in cortical and subcortical regions, while DCA significantly mitigated ischemic injury (Figure 2H,I). Additionally, ROT induced locomotor deficits and anxiety‐like behavior, whereas DCA restored normal activity patterns and reduced anxiety‐related responses (Figure 2J,K).

These findings establish protein lactylation as a critical mediator of poststroke metabolic dysregulation and neuroinflammation. ROT‐induced hyperlactylation exacerbates energy stress and promotes detrimental neurobehavioral outcomes, while DCA‐mediated lactylation suppression affords neuroprotection and accelerates functional recovery. Collectively, these data highlight lactylation as a therapeutic target for modulating metabolic reprogramming after ischemic stroke.

### 2.3 Lactylation Modulation Orchestrates Microglial Phenotypic Switching and Proliferative Restraint after Ischemic Stroke

**Figure 3:**
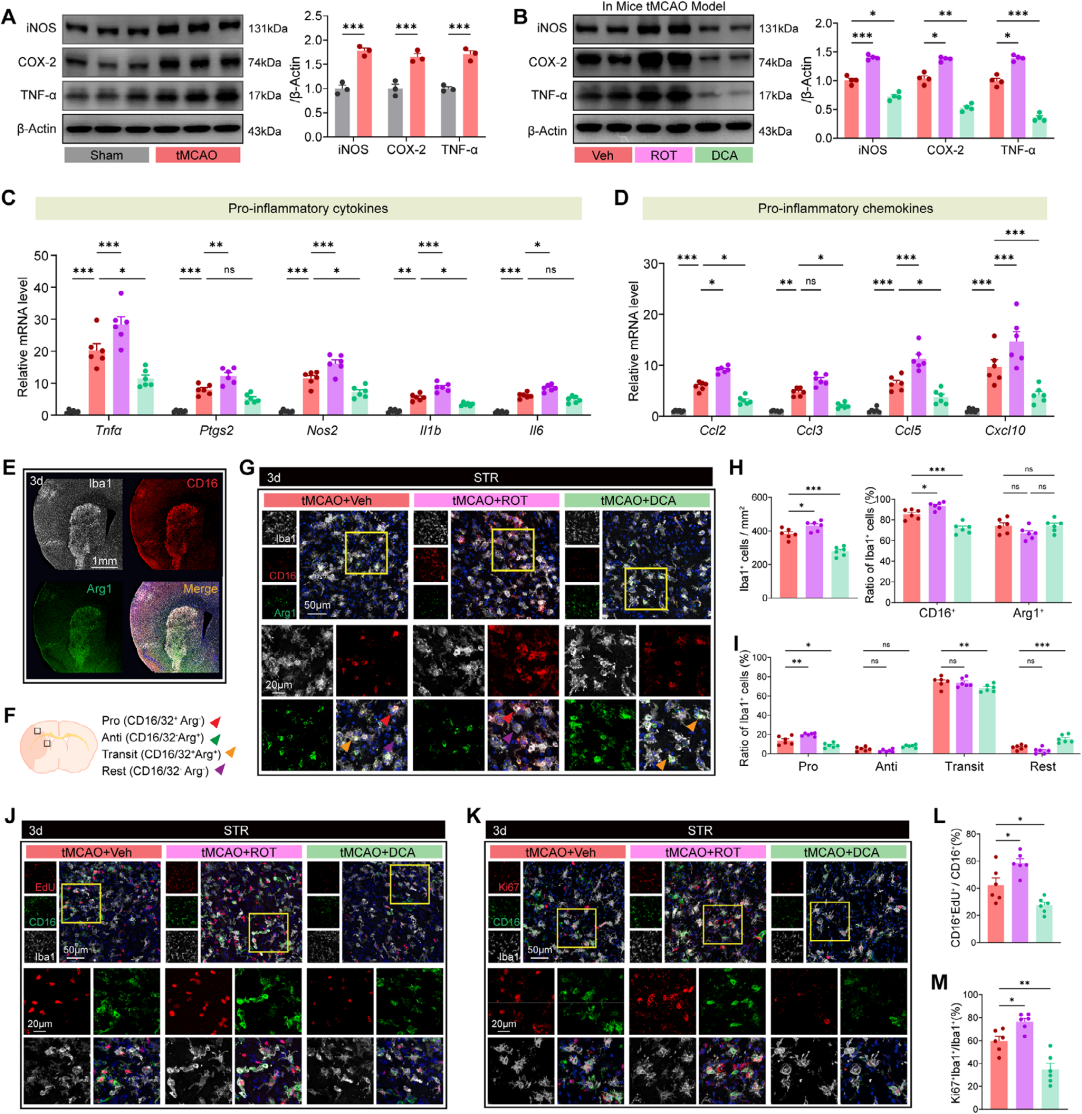
Pharmacological inhibition of protein lactylation mitigates microglial activation and proliferation following cerebral ischemia. A,B) Western blot analysis of inflammatory markers in the ischemic brain of tMCAO mice at 3 days poststroke. *n* = 3 per group. B) Western blot analysis of inflammatory markers in tMCAO mice treated with DMSO (vehicle), Rotenone (ROT), or Dichloroacetate (DCA). *n* = 4 per group. C,D) qPCR analysis of proinflammatory cytokines (*Tnf*, *Ptgs2*, *Nos2*, *Il1b*, *Il6*) and chemokines (*Ccl2*, *Ccl3*, *Ccl5*, *Cxcl10*) in the ischemic brain (*n* = 6 per group). E) Whole‐brain overview of Iba1, CD16, and Arg1 expression at 3 days post‐tMCAO. F) Schematic of microglial polarization classification: proinflammatory (Pro, Iba1^+^/CD16/32^+^Arg1^−^), anti‐inflammatory (Anti, Iba1^+^/CD16/32^−^Arg1^+^), transitional (Transit, Iba1^+^/CD16/32^+^/Arg1^+^), and rest (Rest, Iba1^+^/CD16/32^−^/Arg1^−^). G) Representative immunofluorescence images showing microglial subtypes in the peri‐infarct striatum under different treatment conditions. H) Quantification of Iba1⁺ microglial density and percentages of CD16⁺ and Arg1⁺ microglia. I) Distribution of microglial subtypes based on CD16 and Arg1 expression. J,K) Immunofluorescence staining shows proliferating microglia/CD16⁺ microglia in the striatum. L,M) Quantification of EdU⁺CD16⁺ and Ki67⁺Iba1⁺ cells. Data are presented as mean ± SEM. Statistical analysis was performed using *t*‐test A), or one‐way ANOVA with Tukey's post hoc test B–D,H,I,L,M). ns = not significant, *p < 0.05, **p < 0.01, ***p < 0.001.

In sham‐operated mice, Western blot analysis showed that ROT and DCA did not alter basal levels of proinflammatory mediators (iNOS, COX‐2, TNF‐α), with no significant differences observed among DMSO, ROT, and DCA treatment groups (Figure S2B, Supporting Information). Building on lactylation's established role in metabolic dysregulation following stroke, we next probed its impact on microglial inflammatory polarization and proliferative dynamics. Western blot and qPCR analyses revealed that tMCAO induced robust upregulation of proinflammatory mediators and corresponding mRNAs, with ROT exacerbating and DCA attenuating these responses (**Figure** **3**A–D, Supporting Information). Immunofluorescence imaging showed a marked increase in CD16⁺Iba1⁺ proinflammatory microglia in the striatum and cortex post‐tMCAO, an effect reversed by DCA, which promoted a shift toward a resting (CD16^−^Arg1^−^) phenotype (Figure 3E–I; and Figure S3, Supporting Information). Concomitantly, DCA reduced microglial accumulation in ischemic regions, underscoring its anti‐inflammatory efficacy.

To assess lactylation's role in microglial expansion, we evaluated EdU incorporation (a marker of cell proliferation) and cell‐cycle regulators. Results showed that tMCAO triggered a robust increase in EdU⁺Iba1⁺ microglia, predominantly within the CD16⁺ (proinflammatory) subset. Treatment with DCA significantly dampened EdU signal intensity in both striatum and cortex, reducing the proportion of CD16⁺EdU⁺ cells while sparing Arg1⁺EdU⁺ (anti‐inflammatory) populations (Figure 3J,L; and Figure S4, Supporting Information). Consistent with this, DCA decreased Ki67⁺Iba1⁺ cell numbers, confirming suppression of microglial proliferative activity (Figure 3K,M). These findings establish lactylation as a central regulator of microglial functional plasticity after stroke. Pharmacological inhibition of lactylation with DCA reprograms microglia toward an anti‐inflammatory state, curbing excessive proliferation without compromising reparative phenotypes. This dual regulation of inflammatory and proliferative responses highlights lactylation as a therapeutic node for resolving neuroinflammation and promoting poststroke neurorehabilitation.

### 2.4 MeCP2 Lactylation Regulates Microglial Functional Plasticity via Post‐Translational Reprogramming in Ischemic Brain Injury

**Figure 4:**
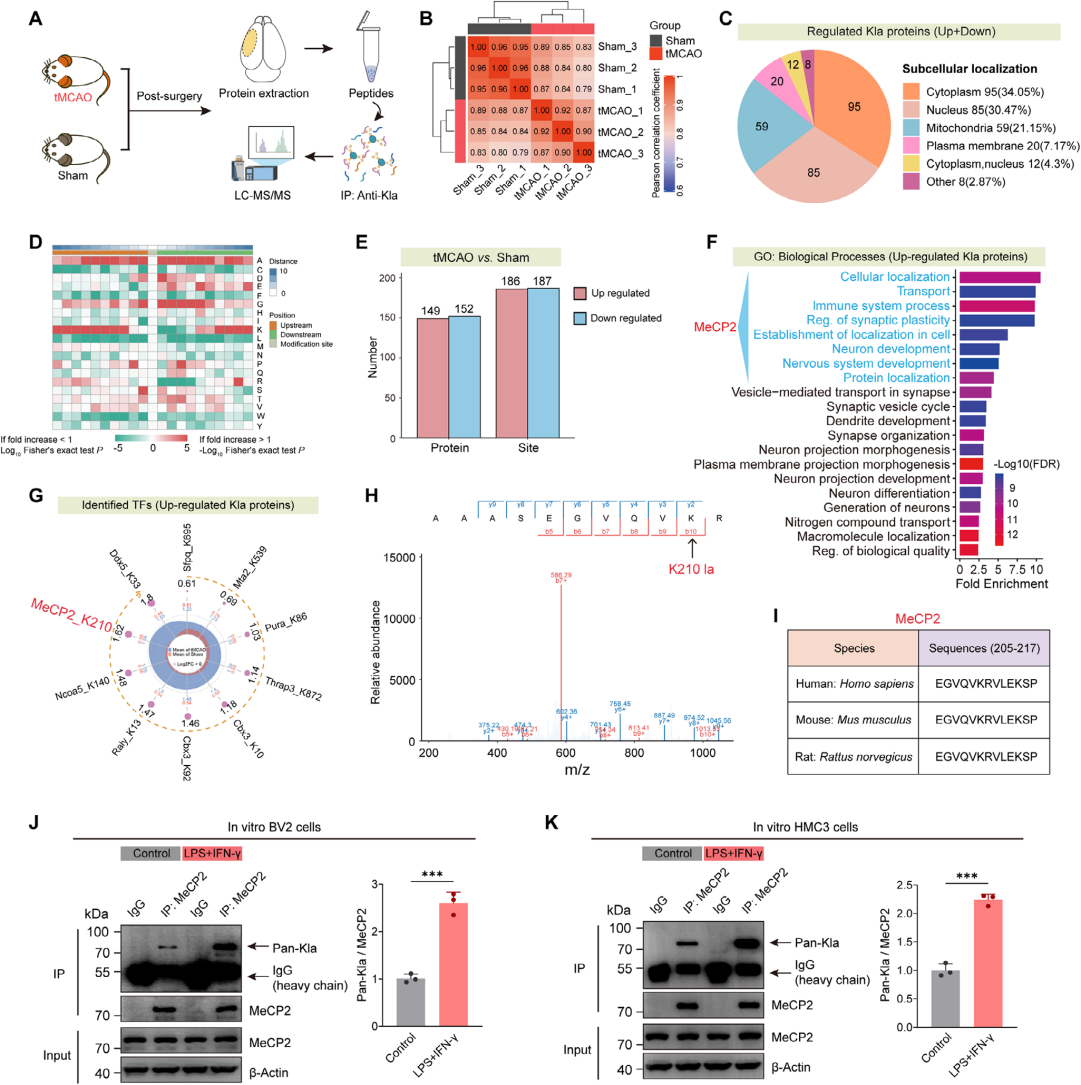
Global proteomic profiling reveals altered lactylation landscape and MeCP2 K210 modification in ischemic brain. A) Schematic workflow of anti‐Kla immunoprecipitation and LC‐MS/MS analysis of brain tissue from sham and tMCAO mice. B) Pearson correlation matrix showing high intragroup reproducibility and distinct clustering between sham and tMCAO groups. C) Pie chart summarizing subcellular localization of differentially lactylated proteins. D) Motif enrichment heatmap indicating sequence preferences flanking lactylation sites. E) Bar graphs summarizing the number of differentially lactylated proteins and sites in tMCAO versus Sham brains. F) Gene Ontology (GO) enrichment analysis of upregulated Kla proteins. G) Transcription factors with increased lactylation in tMCAO, highlighting MeCP2 K210 as a top hit. H) MS/MS spectrum confirming lactylation at lysine 210 of MeCP2. I) Sequence alignment showing conservation of the MeCP2 K210 lactylation site across human, mouse, and rat. J,K) MeCP2 immunoprecipitation (IP) followed by immunoblotting with a pan‐Kla antibody in murine BV2 and human HMC3 cells. *n* = 3 per group. Data are presented as mean ± SD. Statistical analysis was performed using *t*‐test J,K). ***p < 0.001.

Considering the lactylation‐mediated metabolic regulation of microglial responses, we employed Kla‐enriched LC‐MS/MS proteomics to identify key lactylated effectors in ischemic brain tissue. Proteomic analysis of sham and tMCAO mice revealed 373 differentially lactylated sites (fold change ≥2), including 186 upregulated and 187 downregulated sites post‐ischemia (**Figure** **4**A–E). Pearson correlation analysis and subcellular mapping confirmed robust separation of lactylation profiles and enrichment of Kla‐modified proteins in cytoplasmic and nuclear compartments (Figure 4B,C), implicating lactylation in both metabolic signaling and transcriptional regulation. Motif analysis uncovered conserved sequence preferences surrounding lactylated lysines (Figure 4D), while GO enrichment highlighted involvement in intracellular transport, immune signaling, and neuronal development (Figure 4F). Notably, the transcription factor MeCP2 emerged as a prominent lactylated protein, with lysine 210 (K210) identified as a high‐confidence, evolutionarily conserved lactylation site across human, mouse, and rat (Figure 4G–I).

Functional validation in microglia revealed that LPS+IFN‐γ or OGD elevated intracellular lactate, Pan‐Kla levels, and proinflammatory markers (iNOS, COX‐2, TNF‐α) in murine BV2 and human HMC3 cells (Figure S5A–J, Supporting Information). Concomitantly, these conditions enhanced microglial migration and proliferation (Figure S5K–N, Supporting Information). Pharmacological modulation confirmed that lactate accumulation via ROT exacerbated these responses, whereas lactate clearance via DCA suppressed Pan‐Kla, blunted cytokine expression, and reduced microglial motility (Figure S6A–J, Supporting Information).

Despite its functional role, MeCP2 expression remained unaltered across experimental conditions. Single‐cell RNA‐seq, immunofluorescence, and Western blotting consistently showed stable MeCP2 mRNA and protein levels in microglia following tMCAO or inflammatory stimulation (LPS/IFN‐γ) (Figure S7A–I, Supporting Information). However, detailed analyses revealed specific and robust elevation of MeCP2 K210 lactylation: in vitro, LPS/IFN‐γ‐stimulated BV2 microglia exhibited a significant increase in the MeCP2 lacty‐K210/total MeCP2 ratio compared to controls (Figure S7J, Supporting Information); in vivo, western blot of tMCAO mouse brain tissue confirmed a similar elevation in this ratio relative to sham mice (Figure S7K, Supporting Information). Immunofluorescence further demonstrated that this lactylation increase was microglia‐specific—with enhanced MeCP2 lacty‐K210 fluorescence intensity in Iba1⁺ microglia at 3 days post‐tMCAO (Figure S7L, Supporting Information)—and not observed in NeuN⁺ neurons (Figure S7M, Supporting Information).

These findings, together with MeCP2 immunoprecipitation results showing elevated lactylation under inflammatory stress in both murine BV2 and human HMC3 microglial cells (Figure 4J,K), demonstrate that MeCP2 lactylation regulates microglial functional plasticity via post‐translational reprogramming in the context of neuroinflammation.

### 2.5 MeCP2 K210R Mutation Alters Microglial Metabolic Homeostasis and Suppresses Inflammatory Activation

**Figure 5:**
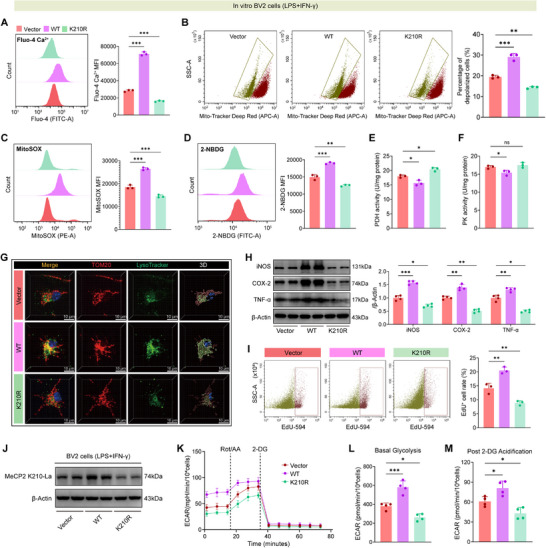
MeCP2 K210 lactylation drives microglial metabolic reprogramming and inflammatory activation, suppressed by K210R mutation. A) Flow cytometry of intracellular calcium levels (Fluo‐4) in murine BV2 cells expressing vector, wild‐type (WT), or K210R‐mutant MeCP2. B) MitoTracker Deep Red staining and quantification of mitochondrial membrane depolarization. C) MitoSOX‐based measurement of mitochondrial ROS. D) Glucose uptake assessed via 2‐NBDG fluorescence. E,F) Enzymatic activity of pyruvate dehydrogenase (PDH, E) and pyruvate kinase (PK, F) in microglial lysates. G) 3D confocal imaging of TOM20⁺ mitochondria and LysoTracker⁺ lysosomes showing mitochondrial‐lysosomal interactions. H) Western blot and quantification of iNOS, COX‐2, and TNF‐α in BV2 cells. I) Flow cytometry plots and quantification of EdU⁺ proliferating BV2 cells. J) Western blot analysis of MeCP2 K210 lactylation in BV2 cells. K) Extracellular acidification rate (ECAR) tracing in BV2 cells following Rot/AA (inhibit mitochondrial respiration) and 2‐DG (inhibit glycolysis) treatment. L,M) Quantification of basal glycolysis L) and glycolytic reserve (post‐2‐DG acidification, M) from ECAR assays. *n* = 3 or 4 per group. Data are presented as mean ± SD. Statistical analysis was performed using one‐way A–F,H,I,L,M) or two‐way ANOVA K) with Tukey's post hoc test. ns = not significant, *p < 0.05, **p < 0.01, ***p < 0.001.

Building on the mechanistic role of MeCP2 K210 lactylation in microglial function, we investigated the nonlactylatable K210R mutant's impact on metabolic reprogramming and neuroinflammation. BV2 cells expressing empty vector control, wild‐type (WT) MeCP2, or Flag‐tagged K210R mutant showed comparable MeCP2 expression (Figure S8A, Supporting Information), excluding confounding effects from protein expression‐level differences. WT MeCP2 disrupted intracellular calcium homeostasis (elevated Fluo‐4 fluorescence signals), induced mitochondrial membrane depolarization, and increased mitochondrial ROS (**Figure** **5**A–C). Concomitantly, WT MeCP2 drove aerobic glycolysis—enhancing 2‐NBDG uptake and suppressing pyruvate dehydrogenase (PDH) activity—whereas K210R restored PDH function, normalized glucose flux, and preserved mitochondrial integrity without altering pyruvate kinase (PK) activity (Figure 5D–F).

Mechanistically, MeCP2 K210 lactylation facilitated mitophagy in microglia, as indicated by increased colocalization efficiency of TOM20⁺ mitochondria with LysoTracker⁺ lysosomes (Figure 5G). This mitophagic response was blunted in K210R‐expressing cells, suggesting preserved mitochondrial function under inflammatory stress. Beyond mitochondrial dynamics, we next examined how MeCP2 K210 lactylation impacts microglial functional phenotypes: WT MeCP2 significantly enhanced expression of proinflammatory mediators (iNOS, COX‐2, TNF‐α; Figure 5H), promoted microglial proliferation (Figure 5I), and augmented transwell migration (Figure S8B, Supporting Information). These proinflammatory and promigratory/proliferative activated microglial phenotypes were markedly attenuated in K210R‐expressing microglia.

Given that WT MeCP2 had already been shown to drive aerobic glycolytic shifts, we next sought to directly assess glycolytic dynamics using extracellular acidification rate (ECAR) assays. Western blotting confirmed specific MeCP2 K210 lactylation in cells expressing WT MeCP2 (and its abolition in BV2 cells expressing the K210R mutant) prior to ECAR analysis (Figure 5J)—ensuring that any observed metabolic differences were attributable to MeCP2 lactylation status rather than variations in protein expression. ECAR tracing showed that WT MeCP2 enhanced glycolytic capacity—defined by the cellular response to perturbation with Rot/AA (rotenone/antimycin A, mitochondrial respiration inhibitors) and 2‐DG (a glycolysis inhibitor)—whereas the K210R mutant blunted this metabolic response (Figure 5K). Quantification of basal glycolysis (Figure 5L) and post‐2‐DG glycolytic reserve (Figure 5M) further demonstrated that the K210R mutation specifically suppressed the glycolytic reprogramming induced by WT MeCP2.

Taken together, these data demonstrate that MeCP2 K210 lactylation drives microglial metabolic reprogramming and inflammatory activation. The K210R mutation rescues oxidative metabolism, suppresses excessive microglial proliferation/migration, and blunts proinflammatory responses—establishing MeCP2 lactylation as a key post‐translational switch in neuroinflammatory pathogenesis.

### 2.6 P300‐Mediated MeCP2 Lactylation Governs Microglial Inflammatory Programming and Impairs Poststroke Neurorehabilitation

**Figure 6:**
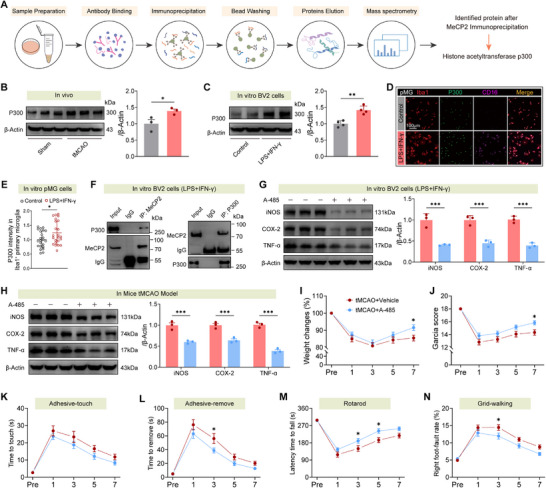
P300 interacts with MeCP2 and drives lactylation‐mediated microglial inflammation and motor dysfunction. A) Schematic workflow of immunoprecipitation‐mass spectrometry (IP‐MS) identifying histone acetyltransferase p300 as a MeCP2‐interacting protein in BV2 cells. B,C) Western blot analysis of p300 in brain tissue post‐tMCAO B) and murine BV2 cells stimulated with LPS+IFN‐γ C). *n* = 3 or 4 per group. D) Immunofluorescence showing increased nuclear p300 in CD16⁺Iba1⁺ primary microglia upon LPS+IFN‐γ stimulation. E) Quantification of p300 intensity in Iba1⁺ microglia (*n* > 20 cells per group). F) Co‐immunoprecipitation (co‐IP) validates direct interaction between MeCP2 and p300 in murine BV2 cells. G) The p300 inhibitor A‐485 suppresses LPS+IFN‐γ‐induced iNOS, COX‐2, and TNF‐α in murine BV2 cells. *n* = 3 per group. H) A‐485 reduces proinflammatory marker expression in the ischemic brain in vivo. *n* = 3 per group. I,J) A‐485 improves body weight recovery and neurological scores post‐tMCAO. *n* = 12 per group. K–N) Behavioral tests demonstrate enhanced sensorimotor function with A‐485: adhesive‐touch K), adhesive‐remove L), rotarod latency M), and grid‐walking error rate N). *n* = 12 per group. Data from animal experiments are means ± SEM; those from cell line experiments are means ± SD. Statistical analysis was performed using *t*‐test B,C,E,G,H), or two‐way ANOVA with Tukey's post hoc test I–N). *p < 0.05, **p < 0.01, ***p < 0.001.

Building on the mechanistic link between MeCP2 lactylation and microglial dysfunction, we sought to identify upstream regulators of this post‐translational modification. To date, specific regulators responsible for lactylation have not been fully explored, though “writers” (e.g., p300) and “erasers” (e.g., SIRT1) of related acetyl/acyl modification systems are well acknowledged.^[^ ^24^ ^]^ To identify lactyltransferases interacting with MeCP2, we performed MeCP2 immunoprecipitation (IP) followed by label‐free quantitative mass spectrometry (MS). This screen identified the histone acetyltransferase p300 (Ep300) as a strong candidate, based on the abundance of specific peptides (Figure S9A, Supporting Information). We subsequently validated p300 as a direct binding partner of MeCP2 through independent coimmunoprecipitation assays followed by western blotting (**Figure** **6**A,F).

Next, we assessed p300 expression and localization: p300 was significantly upregulated in ischemic brain tissue and in LPS+IFN‐γ‐stimulated BV2 microglia, with preferential nuclear localization in CD16⁺Iba1⁺ proinflammatory microglia (Figure 6B–E). These findings implicated p300 as a potential “lactylation writer” for MeCP2. To validate p300's role in lactylation regulation, we used the selective p300 inhibitor A‐485. Pharmacological inhibition of p300 with A‐485 selectively suppressed Pan‐Kla levels: in brain tissue from tMCAO mice, A‐485 reduced Pan‐Kla without altering global protein acetylation (Figure S9B,C, Supporting Information); in LPS/IFN‐γ‐stimulated BV2 microglial cells, A‐485 also decreased Pan‐Kla (Figure S9D, Supporting Information), confirming p300's specificity for regulating lactylation in neuroinflammatory contexts. Functionally, A‐485 treatment blunted expression of proinflammatory markers in both LPS‐stimulated microglia and ischemic mouse brains (Figure 6G,H). Immunofluorescence analysis revealed a marked reduction in iNOS⁺, COX‐2⁺, and TNF‐α⁺ microglia in both cortical and striatal regions post‐tMCAO (Figures S10A–F and S11A–F, Supporting Information), demonstrating regional suppression of microglial activation.

Importantly, p300 inhibition with A‐485 promoted functional recovery after stroke: this was evidenced by attenuated weight loss (Figure 6I), improved Garcia neurological scores (Figure 6J), and enhanced performance in sensorimotor tasks (adhesive‐touch, adhesive‐remove, rotarod, grid‐walking; Figure 6K–N). Altogether, these findings establish p300 as an epigenetic regulator of MeCP2 lactylation, orchestrating microglial proinflammatory phenotypes and impeding poststroke recovery.

### 2.7 MeCP2 Lactylation Coordinates Microglial Inflammatory and Glycolytic Reprogramming via HK2‐Mediated Mitochondrial Stress Axis

**Figure 7:**
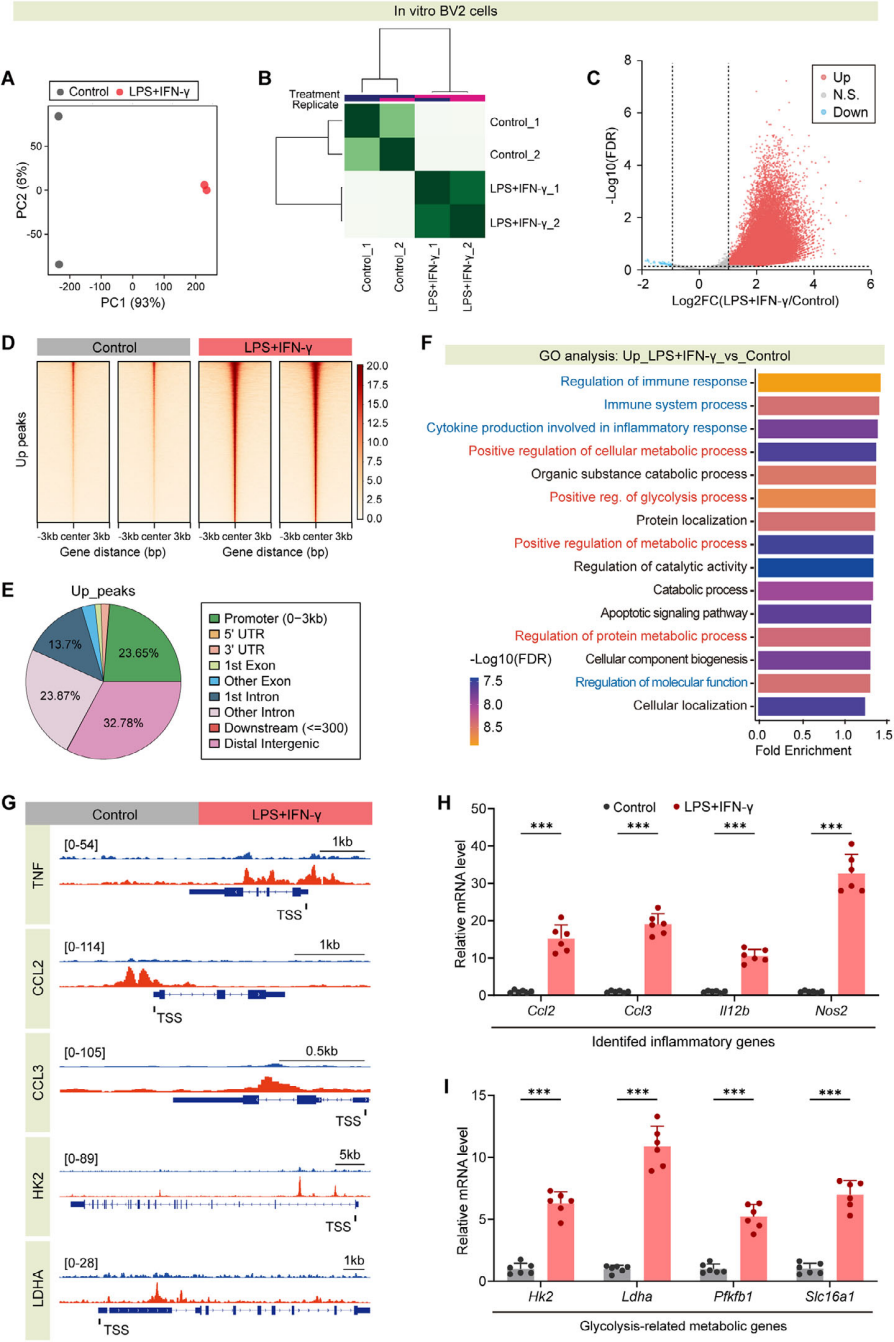
MeCP2 lactylation promotes transcription of inflammatory and glycolysis‐related genes during microglial activation. A) Principal component analysis (PCA) of Cut&Tag profiles shows clear separation between control and LPS/IFN‐γ‐treated groups in murine BV2 cells. B) Hierarchical clustering heatmap of MeCP2‐binding signal intensities demonstrates high intragroup reproducibility. C) Volcano plot highlighting differentially enriched MeCP2‐binding peaks, with a predominance of upregulated sites. D) Heatmap of MeCP2‐binding intensity centered around transcription start sites (TSS), showing global peak enrichment in the LPS+IFN‐γ group. E) Genomic distribution of MeCP2 up‐peaks across promoter, intronic, and intergenic regions. F) Gene Ontology (GO) analysis of MeCP2‐bound genes reveals enrichment in immune response and metabolic processes. G) Genome browser views of MeCP2 occupancy at representative inflammatory (*Tnf*, *Ccl2*, *Ccl3*) and glycolytic (*Hk2*, *Ldha*) gene loci. H,I) qPCR validation of increased expression of proinflammatory H) and glycolytic I) genes following LPS+IFN‐γ stimulation. *n* = 6 per group. Data are shown as mean ± SD. Statistical analysis was performed using *t*‐test H,I). ***p < 0.001.

Leveraging the established role of MeCP2 lactylation in microglial inflammation, we explored its impact on metabolic reprogramming by examining glycolytic and mitochondrial stress pathways. Cut&Tag profiling coupled with high‐throughput sequencing in LPS+IFN‐γ‐stimulated microglia revealed a robust increase in MeCP2 chromatin occupancy at inflammation‐ and glycolysis‐related gene loci, with preferential enrichment near transcription start sites (TSSs) (**Figure** **7**A–D). Principal component analysis (PCA) and hierarchical clustering confirmed distinct chromatin‐binding patterns between control and LPS+IFN‐γ‐treated groups (Figure 7A,B). Peak annotation indicated prominent localization at promoters (Figure 7E), while Gene Ontology (GO) analysis highlighted significant enrichment for immune response (e.g., “immune system process,” “cytokine production involved in inflammatory response”) and glycolytic pathways (e.g., “positive regulation of glycolysis process,” “positive regulation of metabolic process”) (Figure 7F). Integrative Genomics Viewer (IGV) visualization and qRT‐PCR validated MeCP2 binding and upregulation of *Tnf*, *Ccl2*, *Hk2*, and *Ldha* (Figure 7G–I), establishing MeCP2 lactylation as an epigenetic switch linking inflammatory and metabolic activation.

**Figure 8:**
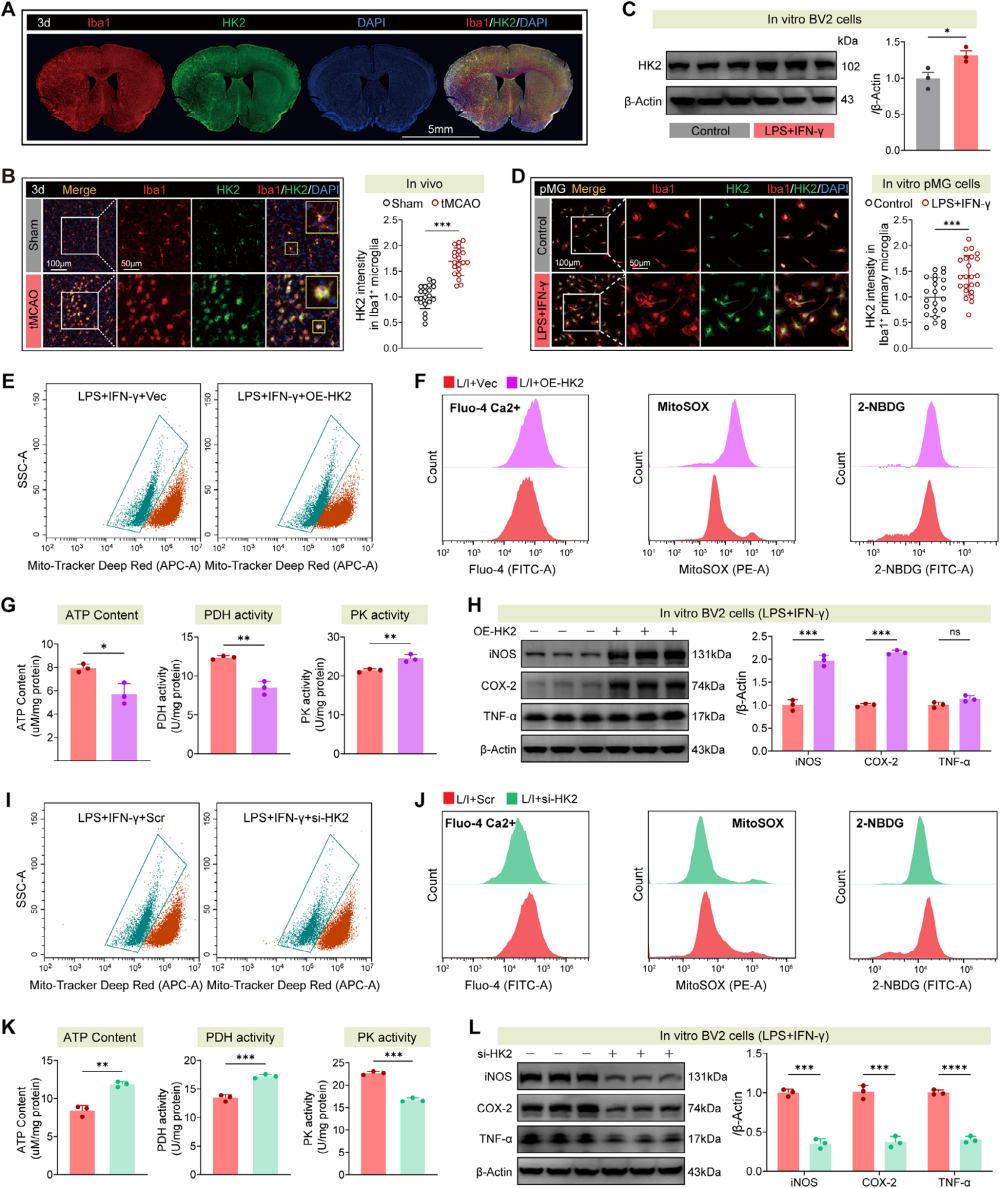
MeCP2–HK2 axis mediates mitochondrial dysregulation and proinflammatory metabolism in microglia under inflammatory stress. A,B) Representative whole‐brain A) and cortical B) immunofluorescence images showing elevated HK2 expression in Iba1⁺ microglia at 3 days post‐tMCAO; quantification of HK2 intensity in Iba1⁺ microglia (B, right). *n* ≥ 20 cells per group. C) Western blot showing increased HK2 protein levels in murine BV2 cells following LPS+IFN‐γ treatment. *n* = 3 per group. D) Primary microglia exhibit increased HK2 expression upon LPS+IFN‐γ stimulation; quantification of HK2 intensity in Iba1⁺ primary microglia (D, right). *n* ≥ 20 cells per group. E,F) HK2 overexpression (OE‐HK2) increases mitochondrial depolarization (MitoTracker), calcium influx (Fluo‐4), mitochondrial ROS (MitoSOX), and glucose uptake (2‐NBDG). G) OE‐HK2 decreases ATP content and pyruvate dehydrogenase (PDH) activity while increasing pyruvate kinase (PK) activity. *n* = 3 per group. H) Western blot shows that HK2 promotes iNOS, COX‐2, and TNF‐α expression. *n* = 3 per group. I,J) HK2 knockdown (si‐HK2) reverses mitochondrial depolarization, Ca^2^⁺ overload, ROS generation, and glucose uptake. K) si‐HK2 increases ATP and PDH activity while suppressing PK activity. *n* = 3 per group. L) si‐HK2 significantly reduces iNOS, COX‐2, and TNF‐α levels in murine BV2 cells. *n* = 3 per group. Data from animal experiments are means ± SEM; those from cell line experiments are means ± SD. Statistical analysis was performed using *t*‐test B–D,G,H,K,L). ns = not significant, *p < 0.05, **p < 0.01, ***p < 0.001.

Subsequent in vivo and in vitro studies identified hexokinase 2 (HK2) as a critical downstream effector of MeCP2. Chromatin immunoprecipitation (ChIP)‐qPCR (Figure S12, Supporting Information) confirmed enhanced MeCP2 binding to the HK2 promoter in LPS+IFN‐γ‐stimulated BV2 cells, validating direct transcriptional regulation. Immunofluorescence analysis further confirmed selective HK2 upregulation in Iba1⁺ microglia following tMCAO, with minimal expression in NeuN⁺ neurons or GFAP⁺ astrocytes (Figure S13, Supporting Information; and **Figure** **8**A–D). In BV2 microglia, HK2 overexpression triggered mitochondrial membrane depolarization, calcium influx, mitochondrial ROS production, and enhanced glycolytic flux (assessed by 2‐NBDG uptake) (Figure 8E,F; and Figure S14A,C, Supporting Information), leading to reduced ATP content, suppressed PDH activity, and elevated PK activity (Figure 8G). These metabolic alterations were accompanied by increased expression of proinflammatory markers (iNOS, COX‐2, TNF‐α) and enhanced microglial proliferation (EdU⁺ cells) (Figure 8H; and Figure S14E, Supporting Information). Conversely, HK2 knockdown restored mitochondrial homeostasis, reduced glycolytic stress, increased ATP levels, and blunted inflammatory gene expression (Figure 8I–L; and Figure S14B,D,F, Supporting Information).

Overall, these findings define a functional MeCP2/HK2 signaling axis in which MeCP2 lactylation promotes microglial activation by regulating HK2‐mediated mitochondrial stress and glycolytic enhancement. This mechanism underscores the metabolic‐epigenetic crosstalk driving neuroinflammatory reprogramming in ischemic stroke.

### 2.8 Targeting HK2 with Lonidamine Rewires mTOR/AMPK Signaling to Suppress Microglial Inflammation and Promote Poststroke Neurorehabilitation

**Figure 9:**
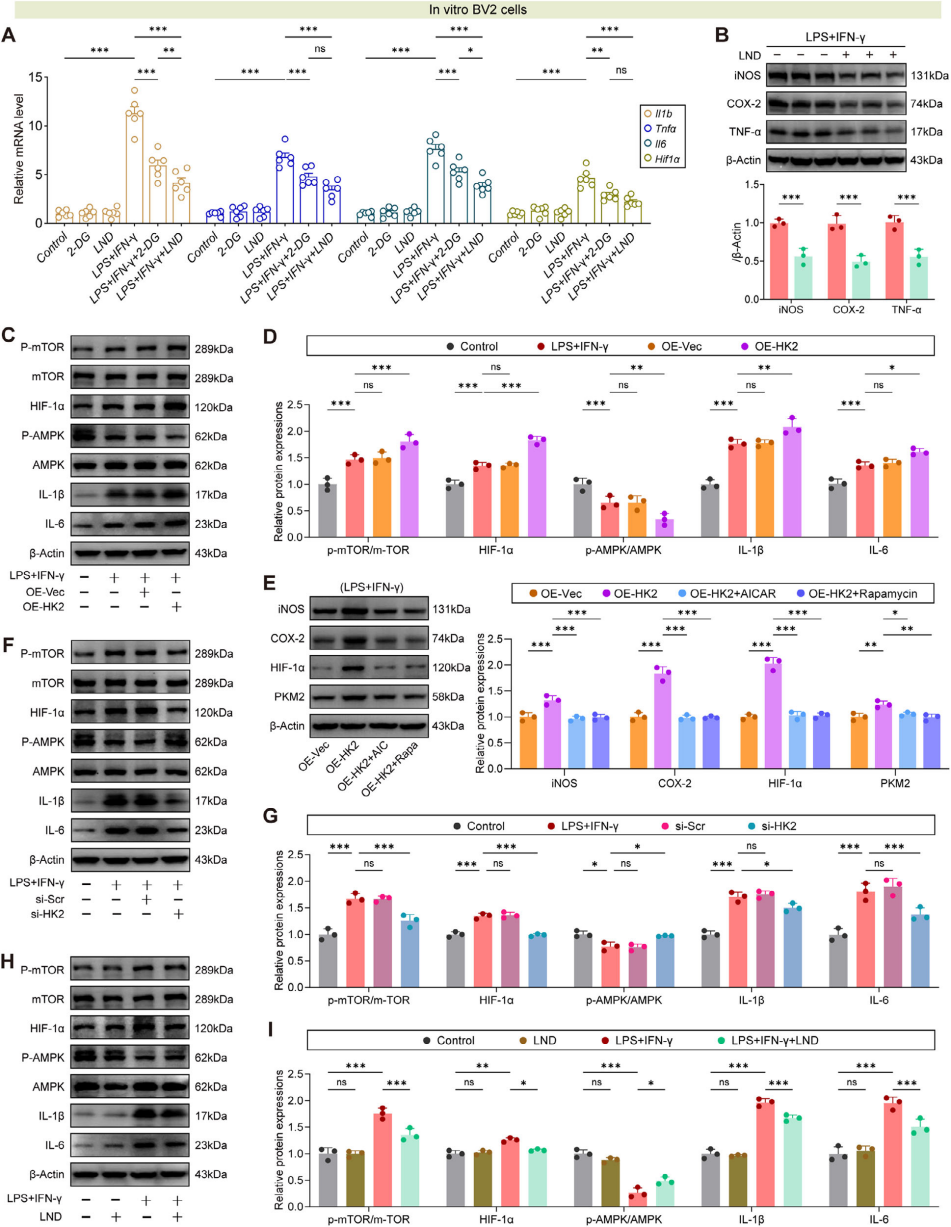
HK2 modulates microglial inflammation via mTOR/AMPK signaling, with functional rescue by AICAR/Rapamycin. A) qPCR analysis of *Il1b*, *Tnf*, *Il6*, and *Hif1α* mRNA in murine BV2 cells treated with control, LPS+IFN‐γ, 2‐DG (glycolytic blocker), or LND (HK2 inhibitor). *n* = 6 per group. B) Western blot analysis of iNOS, COX‐2, and TNF‐α expression with or without LND under LPS+IFN‐γ conditions. *n* = 3 per group. C,D) Western blot and quantification of mTOR/AMPK signaling, HIF‐1α and IL‐1β/IL‐6 in BV2 cells overexpressing empty vector (OE‐Vec) or HK2 (OE‐HK2) under LPS+IFN‐γ stimulation. *n* = 3 per group. E) Western blot and quantification of proinflammatory markers (iNOS, COX‐2, HIF‐1α) and glycolytic regulator PKM2 in BV2 cells overexpressing HK2 (OE‐HK2) with/without treatment of the AMPK agonist AICAR or mTOR inhibitor Rapamycin. *n* = 3 per group. F–G) Western blot and quantification of mTOR/AMPK signaling, HIF‐1α and IL‐1β/IL‐6 in BV2 cells transfected with control siRNA (si‐Scr) or HK2 siRNA (si‐HK2) under LPS+IFN‐γ stimulation. *n* = 3 per group. H,I) Western blot and quantification of mTOR/AMPK signaling, HIF‐1α and IL‐1β/IL‐6 in LPS+IFN‐γ‐stimulated BV2 cells with/without LND treatment. *n* = 3 per group. Data are presented as mean ± SD. Statistical analysis was performed using *t*‐test (B), or one‐way ANOVA with Tukey's post hoc test A,D,E,G,I). ns = not significant, *p < 0.05, **p < 0.01, ***p < 0.001.

Given the critical role of HK2 in mediating MeCP2 lactylation‐driven metabolic reprogramming, we evaluated whether selective HK2 inhibition could modulate microglial function and poststroke recovery. Using 2‐deoxy‐D‐glucose (2‐DG, a general glycolytic inhibitor) and lonidamine (LND, a selective HK2 antagonist), we probed the impact of glycolytic blockade on inflammatory signaling. In LPS+IFN‐γ–stimulated microglia, LND potently suppressed transcription of *Il1b*, *Tnf*, *Il6*, and *Hif1α* compared to 2‐DG (**Figure** **9**A), with corresponding reductions in iNOS, COX‐2, and TNF‐α protein levels (Figure 9B).

Mechanistically, HK2 overexpression drove the upregulation of glycolysis‐driving (HIF‐1α) and proinflammatory (IL‐1β, IL‐6) molecules via enhancing mTOR phosphorylation and repressing AMPK activation (Figure 9C,D). To establish causal involvement of the mTOR/AMPK axis in HK2‐mediated phenotypic reprogramming, we performed functional rescue experiments. Specifically, we treated HK2‐overexpressing BV2 cells with the AMPK agonist AICAR or the mTOR inhibitor rapamycin—this significantly attenuated the upregulation of proinflammatory mediators (iNOS and COX‐2), the master transcriptional regulator of glycolysis, HIF‐1α, and its downstream glycolytic effector PKM2 (Figure 9E). Conversely, HK2 silencing or LND treatment restored p‐AMPK levels and blunted proinflammatory cytokine production (Figure 9F–I). These data establish HK2 as a metabolic checkpoint linking glycolytic flux to mTOR/AMPK‐mediated cytokine production, where AICAR/rapamycin rescued the inflammatory phenotype induced by HK2 overexpression.

**Figure 10:**
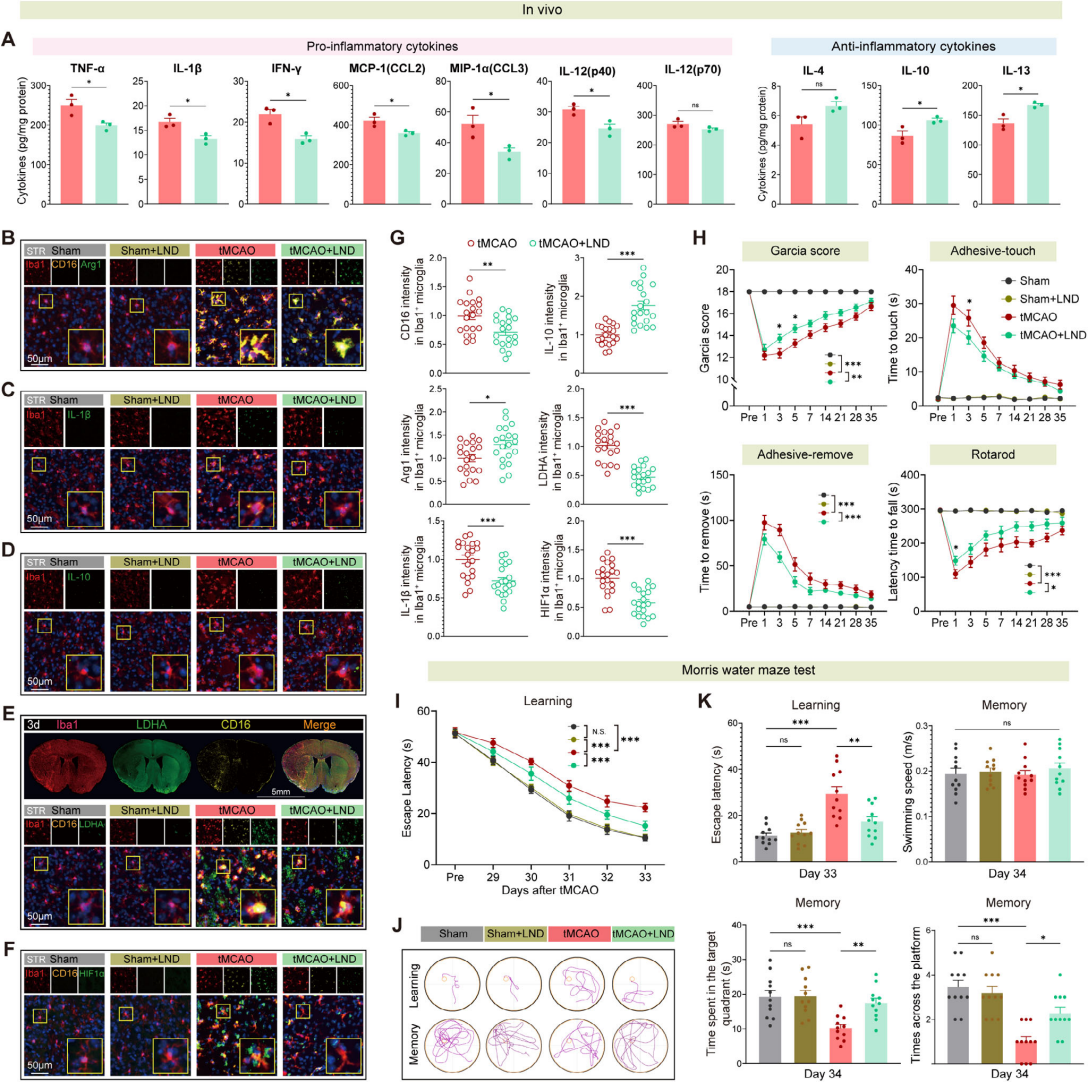
Inhibition of HK2 by lonidamine reprograms microglial phenotype, reduces neuroinflammation, and improves long‐term functional recovery after ischemic stroke. A) ELISA quantification of proinflammatory cytokines (TNF‐α, IL‐1β, IFN‐γ, MCP‐1/CCL2, MIP‐1α/CCL3), proinflammatory IL‐12 subunits (IL‐12p40, IL‐12p70), and anti‐inflammatory cytokines (IL‐4, IL‐10, IL‐13) in brain tissue at day 3 post‐tMCAO (*n* = 3 per group). B–D) Representative immunofluorescence images showing Iba1 costained with CD16 and Arg1 B), IL‐1β C), IL‐10 D) in the peri‐infarct striatum at 3 days post‐tMCAO. E,F) Immunofluorescence analysis of LDHA and HIF‐1α expression in Iba1⁺ microglia at 3 days postinjury. G) Quantification of fluorescence intensity of LDHA, CD16, Arg1, IL‐1β, IL‐10, and HIF‐1α in Iba1⁺ microglia (*n* ≥ 20 cells per group). H) Longitudinal behavioral tests: Garcia neurological score, adhesive‐touch, adhesive‐remove, and rotarod tests demonstrate improved motor recovery in LND‐treated mice. *n* = 11 per group. I,K) Morris water maze performance: reduced escape latency I) and increased time spent in the target quadrant K) reflect improved spatial learning and memory in the LND‐treated group. *n* = 11 per group. J) Representative swim paths during training (top) and probe trials (bottom) across groups. Data are presented as mean ± SEM. Statistical analysis was performed using *t*‐test A,G), one‐way K) or two‐way ANOVA H,I) with Tukey's post hoc test. ns = not significant, *p < 0.05, **p < 0.01, ***p < 0.001.

In vivo, LND treatment at 3 days post‐tMCAO significantly reduced Ki67⁺Iba1⁺ and pH3⁺Iba1⁺ microglia in the striatum and cortex, indicating suppressed proliferation (Figure S15A–J, Supporting Information). ELISA revealed that LND suppressed brain levels of TNF‐α, IL‐1β, IFN‐γ, and chemokines while elevating anti‐inflammatory cytokines IL‐4, IL‐10, and IL‐13 (**Figure** **10**A). Immunofluorescence confirmed LND‐induced microglial phenotypic switching, reducing CD16⁺, IL‐1β⁺, LDHA⁺, and HIF‐1α⁺ microglial cells while increasing Arg1⁺ and IL‐10⁺ populations (Figure 10B–G; and Figure S16A–E, Supporting Information). Behaviorally, LND‐treated mice showed improved Garcia scores, reduced sensorimotor latencies, and enhanced rotarod performance up to 35 days poststroke (Figure 10H). Morris water maze testing revealed faster escape latency, increased target zone dwell time, and more platform crossings (Figure 10I–K), confirming restored spatial cognition.

In summary, these findings establish HK2 as a central regulator of microglial metabolic‐inflammatory crosstalk. LND‐mediated HK2 inhibition rewires mTOR/AMPK signaling, restores mitochondrial homeostasis, suppresses pathological microglial activation, and promotes long‐term functional recovery—highlighting HK2 as a therapeutic target for ischemic stroke.

## 3 Discussion

Our study identifies lysine lactylation as a pivotal epigenetic mechanism governing microglial activation and dysfunction after ischemic stroke. Notably, stroke‐induced lactate surges selectively trigger lactylation of MeCP2 at lysine 210 (K210) in microglia—a post‐translational modification that potentiates the activation of glycolytic (e.g., HK2) and proinflammatory target genes. This epigenetic reprogramming initiates a self‐reinforcing metabolic‐inflammatory cascade, characterized by mitochondrial dysfunction and dysregulation of the mTOR/AMPK signaling axis. Genetic ablation of the MeCP2 K210 lactylation site (via K210R mutation) or pharmacological inhibition of upstream lactyltransferase p300 reverses this pathological cascade, establishing MeCP2 lactylation as a druggable metabolic‐epigenetic node in ischemic stroke pathogenesis.

The MeCP2/HK2 axis serves as a critical molecular link between metabolic and inflammatory reprogramming: lactylation‐induced HK2 upregulation promotes glycolysis, fueling microglial proliferation and proinflammatory polarization. This finding uncovers a functional metabolic‐inflammatory crosstalk mechanism that connects metabolic stress to epigenetic regulation in brain immunity, explaining how ischemic injury primes microglial dysfunction. Functionally, targeting this axis with the HK2 inhibitor lonidamine restores mitochondrial function, suppresses neuroinflammation, and improves long‐term neurological outcomes in preclinical stroke models, underscoring its translational potential.

The regulatory role of MeCP2 in microglial metabolic and inflammatory networks is consistent with prior investigations in neurodevelopmental and neuroinflammatory pathologies.^[^ ^25^ ^]^ In MeCP2‐null microglia, metabolic homeostasis is profoundly disrupted, characterized by increased oxygen consumption, elevated reactive oxygen species (ROS), and compromised ATP synthesis—hallmarks of energetic dyshomeostasis.^[^ ^26^ ^]^ Mechanistically, this phenotype is driven in part by upregulation of the glutamine transporter SLC38A1 (SNAT1), which perturbs glutamine metabolism and triggers mitochondrial dysfunction. Notably, targeted antioxidant therapies that restore mitochondrial integrity alleviate these metabolic abnormalities, highlighting the therapeutic relevance of redox homeostasis in MeCP2‐associated microglial dysfunction.^[^ ^27^ ^]^

Concomitantly, metabolic derangements in MeCP2‐deficient microglia are accompanied by pronounced neuroinflammatory activation. Transcriptomic analyses reveal sustained upregulation of hypoxia‐ and glucocorticoid‐responsive genes, indicative of a chronic proinflammatory state.^[^ ^23^ ^]^ Enhanced receptor‐interacting serine/threonine‐protein kinase 1 (RIPK1) signaling drives cytokine overproduction and oxidative stress in these cells, while also promoting expression of glutamate transporters (e.g., SLC7A11, GLS).^[^ ^28^ ^]^ Pharmacological inhibition of RIPK1 not only suppresses inflammatory cascades but also restores neurotransmitter balance and improves motor function, positioning RIPK1 as a validated therapeutic target. Additionally, MeCP2 directly modulates transcriptional responses to tumor necrosis factor (TNF) signaling, further reinforcing its role as a central immunometabolic checkpoint.^[^ ^23^ ^]^ Collectively, these findings establish MeCP2 as a molecular nexus integrating microglial bioenergetics and immune responsiveness. Beyond this, excitotoxicity is another critical consequence of MeCP2 loss in microglia. Enhanced activity of the Xc^−^ antiporter drives excessive extracellular glutamate release, exacerbating neuronal damage.^[^ ^29^ ^]^ Targeted nanotherapies, such as dendrimer‐conjugated N‐acetyl cysteine (D‐NAC), bypass this transporter to mitigate oxidative stress, yielding behavioral improvements in MeCP2‐null models. Complementary strategies, including dendrimer‐conjugated glutaminase inhibitors (e.g., D‐JHU29), normalize glutamate metabolism, exemplifying the potential of precision nanomedicine to correct MeCP2‐associated microglial dysfunction.^[^ ^30^ ^]^

Beyond its role in energy and neurotransmitter homeostasis, MeCP2 orchestrates microglial modulation of synaptic architecture and function. MeCP2‐deficient microglia exhibit excessive synaptic engulfment, driving progressive synaptic loss and neural circuit dysfunction.^[^ ^31^, ^32^ ^]^ Notably, exclusive restoration of MeCP2 in microglia only partially rescues these deficits, underscoring the complexity of neuron–microglia crosstalk in MeCP2‐associated pathologies.^[^ ^33^ ^]^ In preclinical models of addiction and depression, MeCP2 phosphorylation and nuclear translocation regulate microglial brain‐derived neurotrophic factor (BDNF) expression, thereby modulating synaptic plasticity and behavioral adaptation.^[^ ^34^, ^35^ ^]^ Pharmacological activation of this MeCP2 phosphorylation‐nuclear translocation axis with sulforaphane (SFN) enhances stress resilience and dendritic remodeling, offering a novel therapeutic paradigm for mood disorders.

MeCP2 also drives epigenetic reprogramming of microglia in neurodegenerative and infectious diseases. In Alzheimer's disease, MeCP2 cooperates with chromatin modifiers to repress proinflammatory transcription,^[^ ^25^ ^]^ while HIV‐1 Tat‐induced MeCP2 upregulation amplifies inflammation via suppression of microRNA‐124 (miR‐124),^[^ ^36^ ^]^ underscoring conserved epigenetic regulatory mechanisms across pathological contexts. These findings establish MeCP2 as a multifunctional regulator of microglial metabolic reprogramming, immune homeostasis, and neurotoxicity. Therapeutic strategies targeting glutamate metabolism, mitochondrial bioenergetics, or transcriptional repression have shown promise in MeCP2‐associated neuroinflammatory disorders (e.g., Rett syndrome).^[^ ^30^, ^37^ ^]^ Building on this, our study extends this paradigm by identifying lactylation as a gain‐of‐function post‐translational modification that reprograms MeCP2 toward a proinflammatory metabolic phenotype specifically in ischemic stroke.

Notably, MeCP2 lactylation exhibits context‐dependent functional dichotomy across cell types. While our study defines a proinflammatory role for MeCP2 K210 lactylation in poststroke microglia, another recent work demonstrates that neuronal MeCP2 lactylation at K210/K249 exerts neuroprotection via the transcriptional repression of apoptotic genes.^[^ ^14^ ^]^ This cell‐type specificity underscores the nuanced regulation of lactylation‐driven transcription, likely shaped by cell‐specific chromatin landscapes and cofactor availability. Peripherally, MeCP2 K271 lactylation modulates macrophage polarization and vascular inflammation,^[^ ^38^, ^39^ ^]^ yet these functions differ from its proinflammatory role in ischemic microglia. These findings highlight how lysine site specificity and chromatin context dictate the multifaceted roles of MeCP2 lactylation. Our study further defines a pathological MeCP2 K210/HK2 axis in stroke, suggesting cell‐type‐specific modulation of lactylation as a precision strategy for neuroinflammatory and cardiovascular diseases.

HK2 emerges as a critical metabolic transducer of MeCP2 lactylation in microglia, linking epigenetic control to glycolytic reprogramming. In AD, microglial HK2 upregulation induces hyperglycolytic states that sustain chronic inflammation, while HK2 inhibition redirects metabolism toward lipid oxidation, enhancing β‐amyloid clearance.^[^ ^40^ ^]^ This contextual duality is mirrored in stroke: hypoxic HK2 hyperactivation amplifies glycolysis‐IL‐1β crosstalk, whereas selective inhibition (e.g., with LND) suppresses microglial activation without compromising global bioenergetics.^[^ ^41^ ^]^

However, the role of HK2 in neuroinflammation is complex. Global HK2 deficiency exacerbates mitochondrial dysfunction and neuronal injury in stroke and intracerebral hemorrhage models,^[^ ^42^ ^]^ while microglia‐specific HK2 deletion confers limited benefit in neuropathic pain, implicating peripheral myeloid cells in HK2‐mediated pathology.^[^ ^43^ ^]^ In AD, global HK2 ablation exacerbates neuroinflammation, whereas partial inhibition with LND preserves mitochondrial function while modulating nuclear factor‐κB (NF‐κB) signaling.^[^ ^44^ ^]^ These data reinforce the need for context‐ and cell‐type‐specific HK2 targeting. Our findings align with this paradigm: LND selectively reduces microglial proliferation and inflammation poststroke, driving functional recovery by balancing metabolic integrity and immune homeostasis.

Prior studies highlight HK2 as a critical regulator of microglial proliferative capacity and immune surveillance. Following colony‐stimulating factor 1 receptor (CSF1R) inhibition, HK2‐deficient microglia exhibit impaired CNS repopulation, characterized by reduced 5‐ethynyl‐2′‐deoxyuridine (EdU) incorporation and mitotic gene expression, independent of cell survival, or migration.^[^ ^45^ ^]^ In photothrombotic stroke models, HK2‐null mice exhibit exacerbated infarct volume, neuronal loss, and sensorimotor deficits; single‐cell profiling reveals upregulated proinflammatory genes (e.g., *Il1b*, *Tnf*) and reduced phagocytic markers.^[^ ^45^ ^]^ These findings contrast with our tMCAO model, potentially due to differences in vascular injury patterns (superficial cortical vs large‐vessel occlusion) and microglial activation state stratification. Future studies using filament‐based occlusion models will clarify how HK2 deletion impacts the expansion of CD16⁺IBA1⁺ proinflammatory microglia.

Our findings identify MeCP2 K210 lactylation as a critical upstream regulator of mTOR activation in ischemic microglia, extending previous reports implicating MeCP2 in mTOR signaling modulation. For instance, MeCP2 deficiency enhances induced pluripotent stem cell (iPSC) reprogramming efficiency via augmentation of insulin‐like growth factor 1 (IGF1)/AKT/mTOR signaling, which increases ribosomal biogenesis and cell cycle gene translation.^[^ ^46^ ^]^ Together with our results, these observations suggest a conserved role for MeCP2 in tonically suppressing mTOR activity. Loss of MeCP2 expression (as in the iPSC study) or post‐translational modification via lactylation (as shown here) relieves this repression, leading to mTOR pathway activation.

Our work aligns with emerging evidence of lactylation as a conserved metabolic–epigenetic mechanism across pathological contexts. In AD, histone H4 lysine 12 (H4K12) lactylation forms a proglycolytic feedback loop with pyruvate kinase M2 (PKM2).^[^ ^47^ ^]^ In aging, histone H3 lysine 18 (H3K18) lactylation activates NF‐κB.^[^ ^48^ ^]^ In glioblastoma, tumor‐derived lactate enhances macrophage IL‐10 transcription via histone lactylation.^[^ ^49^ ^]^ In diabetic retinopathy, lactylation cooperates with fat mass and obesity‐associated protein (FTO)‐mediated m6A RNA modification to drive endothelial inflammation and vascular leakage.^[^ ^50^ ^]^ These findings, together with our prior work on HDAC3/PU.1‐mediated epigenetic reprogramming,^[^ ^51^, ^52^ ^]^ collectively suggest a unified paradigm: metabolic stressors drive inflammatory transcriptional outputs via chromatin modification.

The MeCP2/HK2/mTOR axis uncovered in this study reveals a novel metabolic–epigenetic circuit driving postischemic microglial activation. Ischemia‐induced lactate promotes MeCP2 K210 lactylation, sustaining transcription of glycolytic and proinflammatory genes. The pathophysiological role of HK2 in this axis is supported by Codocedo et al.,^[^ ^44^ ^]^ who demonstrated that microglial HK2 upregulation drives neuroinflammation in AD, consistent with our observation of HK2 elevation in ischemic microglia. Importantly, Codocedo et al. also reported a dosage‐dependent effect of HK2 inhibition: partial suppression reduced neuroinflammation without compromising mitochondrial function, whereas complete ablation was harmful. This underscores the therapeutic potential of moderate HK2 inhibition, corroborated by our data showing that partial HK2 inhibition with LND is safe and effective in ischemic stroke models.

Notably, LND may offer benefits beyond HK2 inhibition. Chen et al.^[^ ^53^ ^]^ identified LND as a broad‐spectrum inflammasome inhibitor that directly binds apoptosis‐associated speck‐like protein containing a CARD (ASC) to block inflammasome assembly, independent of HK2. In our study, LND attenuated HK2‐mediated glycolysis and ASC‐dependent caspase‐1 activation/IL‐1β maturation. This dual targeting is especially promising in stroke, where metabolic dysfunction and inflammasome activation coexist, potentially offering superior efficacy to single‐target agents. Critically, LND's existing clinical approval for oncology^[^ ^54^ ^]^ may expedite its repurposing for neuroinflammatory conditions.

Prior work provides critical precedent for the brain delivery of LND, both in its native form and via engineered delivery systems. For example, LND conjugated to a blood–brain barrier (BBB)‐penetrating T7 peptide and loaded into hollow mesoporous Prussian blue nanoparticles (LND@HMPB‐T7) accumulates efficiently in the brain, modulating microglial metabolism and enhancing amyloid‐β clearance in an AD model.^[^ ^55^ ^]^ Ye et al.^[^ ^56^ ^]^ further demonstrated that mitochondria‐targeted LND can be functionalized onto black phosphorus nanosheets and encapsulated in macrophage membranes, which crosses the BBB to induce pyroptosis in glioblastoma. Additionally, Liu et al.^[^ ^57^ ^]^ developed pH‐sensitive liposomes coloaded with LND and doxorubicin that traverse the BBB via p‐hydroxybenzoic acid/dopamine receptor‐mediated targeting, yielding synergistic antiglioma effects. These studies confirm that LND is amenable to advanced formulation strategies that enhance its brain bioavailability—a pivotal consideration for clinical translation of our stroke findings.

In the translational context, p300 inhibitors have advanced from preclinical optimization to early clinical evaluation. Dysregulated activity of KAT3 family histone acetyltransferases (CBP/p300) drives oncogenic transcriptional programs and maladaptive stress responses, prompting the development of small‐molecule inhibitors spanning late preclinical to early clinical stages.^[^ ^58^ ^]^ First‐generation catalytic inhibitors (e.g., A‐485), which target the CBP/p300 histone acetyltransferase (HAT) domain, validated this target's druggability in cancer via lineage‐specific transcriptional suppression.^[^ ^58^ ^]^ Subsequent preclinical agents (spirohydantoin‐based inhibitors, covalent acrylamide derivatives) exhibit durable target occupancy but require optimization to mitigate systemic toxicity.^[^ ^59^, ^60^, ^61^ ^]^ Clinically advanced agents target the CBP/p300 bromodomain: CCS1477 (inobrodib), currently in phase I/IIa trials for malignancies, disrupts enhancer binding and exhibits preliminary antitumor activity.^[^ ^62^, ^63^ ^]^ Dual‐targeting agents (e.g., NEO2734, a dual BET/CBP/p300 inhibitor) are in early trials for solid tumors and leukemia.^[^ ^64^, ^65^ ^]^ Beyond oncology, A‐485 modulates leukocyte mobilization and cytokine responses, thereby highlighting its potential for inflammatory indications while underscoring the need to monitor host defense.^[^ ^66^ ^]^ These advances confirm p300 inhibitors as promising translational candidates for targeting MeCP2 lactylation in ischemic stroke.

Despite these insights, several study limitations merit consideration. First, while p300 was identified as the primary enzyme mediating MeCP2 lactylation, the potential involvement of other histone acetyltransferases (e.g., CBP) remains uninvestigated. Second, our findings were derived from the tMCAO model; validation in photothrombotic or endothelin‐1‐induced ischemia models would enhance translational relevance by accounting for differences in vascular injury patterns. Third, the focus on HK2‐mediated glycolysis may have overlooked roles for oxidative phosphorylation and lipid metabolism in lactylation‐driven microglial dysfunction; future multiomics studies are needed to define the broader metabolic consequences of this epigenetic modification. To strengthen the translational rationale for targeting the MeCP2/HK2 axis, further work should prioritize: 1) delineation of LND's pharmacokinetic profile and therapeutic window in cerebral ischemia models; 2) exploration of delivery technologies (e.g., peptide conjugation, nanocarriers) to optimize BBB penetration and microglial targeting; and 3) investigation of cell‐type‐specific lactylation modulators to minimize off‐target effects. Addressing these gaps will be critical for advancing our mechanistic findings toward clinically actionable strategies for mitigating postischemic neuroinflammation.

## 4 Conclusions

Our study identifies MeCP2 K210 lactylation as a novel epigenetic rheostat that bridges ischemic metabolic stress to pathological microglial activation. Mechanistically, this lactylation drives sustained neuroinflammation and mitochondrial impairment by upregulating HK2 and disrupting the mTOR/AMPK signaling axis, establishing a clear causal chain between epigenetic modification, metabolic reprogramming, and inflammatory pathology. Targeting the MeCP2/HK2 axis exhibits strong translational potential for ischemic stroke and broader neuroinflammatory disorders, with unique clinical feasibility: the HK2 inhibitor lonidamine, which improved outcomes in our preclinical stroke models, already has clinical research support for metabolic conditions; meanwhile, emerging p300‐targeted lactylation modulators (targeting MeCP2's upstream lactyltransferase) provide novel, pathway‐specific intervention points. This dual potential—combining well‐characterized agents and innovative regulators—thus reinforces the translation of our mechanistic findings into clinically actionable strategies for mitigating postischemic neuroinflammation.

## 5 Experimental Section

### Study Participants

The participants were drawn from the Minhang Stroke Cohort, a cohort established at Minhang Hospital (Shanghai, China) that focuses on patients with ischemic stroke. Briefly, between January 2019 and December 2023, patients aged ≥18 years diagnosed with ischemic stroke were recruited from the Department of Neurology at Minhang Hospital. Patients were excluded if the interval from symptom onset to hospital admission exceeded 7 days or if they had a confirmed tumor diagnosis. A total of 789 participants were included in the analysis, and these were divided into two outcome groups based on 3‐month modified Rankin Scale (mRS) scores: the favorable outcome group (mRS score 0–2, *n* = 525) and the unfavorable outcome group (mRS score >2, *n* = 264). Details of these groups are provided in Tables S1 and S2 (Supporting Information). Informed consent was obtained from all participants, and the study was approved by the Institutional Ethics Committee of Minhang Hospital, Fudan University (Approval No. 2021‐008‐01K). All procedures were conducted in accordance with the Declaration of Helsinki.

### Measurement of Serum LDH and Lactate Levels

Fasting venous blood samples were collected within 24 h of hospital admission. Serum lactate dehydrogenase (LDH) levels were measured using a Cobas 8000 automatic analyzer (Roche Diagnostics, Indianapolis, IN). Laboratory personnel conducting these assays were blinded to participants’ clinical characteristics and outcomes. Serum lactate levels were assessed in 20 healthy controls and 20 ischemic stroke patients.

### Animals

This study used male C57BL/6 mice aged 10–12 weeks, weighing 23–28 g. Mice were obtained from SiPeiFu Animal Center (Beijing, China) and housed under controlled environmental conditions, with a temperature of 22 ± 1 °C and a relative humidity of 65%–70%. They were maintained on a 12‐h light/dark cycle with ad libitum access to standard laboratory chow and water. All experimental procedures complied with the National Institutes of Health (NIH) Guide for the Care and Use of Laboratory Animals and were approved by the Department of Laboratory Animal Science, Fudan University (Approval No. 2023‐MHFY‐23JZS). Measures were taken to minimize discomfort and distress throughout the study.

### tMCAO Model

A tMCAO model was established to induce transient focal cerebral ischemia in male C57BL/6 mice. To maintain experimental rigor, animals were randomized, and all procedures were performed under blinded conditions, following a modified protocol based on established methodologies.^[^ ^51^ ^]^ Anesthesia was induced using 4% isoflurane and maintained at 2% isoflurane in a mixture of oxygen and air. Body temperature was regulated at 37 ± 0.5 °C with a heating pad during surgery. A midline cervical incision was made to expose the left common carotid artery, internal carotid artery (ICA), and external carotid artery (ECA). After ligating the ECA, a silicon‐coated intraluminal filament was inserted through the ECA stump into the ICA to occlude the middle cerebral artery (MCA). Successful occlusion was confirmed when mild resistance was felt. The filament remained in place for 60 min to induce ischemia, after which it was carefully withdrawn to allow reperfusion. Sham‐operated mice underwent identical procedures but without MCA occlusion. Following surgery, all animals were monitored under controlled conditions during recovery.

### EdU Labeling

To track proliferating cells, EdU (50 mg kg^−1^, ST067, Beyotime, China) was intraperitoneally administered every 12 h for 1–3 days post‐tMCAO. Microglial proliferation was evaluated using the BeyoClick EdU Cell Proliferation Kit (Alexa Fluor 594, C0078, Beyotime, China).

### Immunofluorescence Staining

On postoperative day 3, mice were anesthetized with 2% isoflurane and euthanized by decapitation. The brains were carefully removed, fixed overnight at 4 °C in 4% paraformaldehyde in PBS, and then transferred to 30% sucrose solution for cryoprotection until fully equilibrated. Once cryoprotected, coronal brain sections (25 µm thick) were obtained using a freezing microtome (CM 1900, Leica, Germany). Staining procedure: Free‐floating sections were washed with PBS to remove any remaining fixative and then incubated in blocking buffer (5% normal goat serum, 1% Triton X‐100 in PBS) at room temperature for 1 h to prevent nonspecific binding. Sections were incubated overnight at 4 °C with primary antibodies. The following primary antibodies were used: Goat anti‐Iba1 (Abcam, ab5076), Rabbit anti‐Iba1 (Abcam, ab178846), Chicken anti‐GFAP (Abcam, ab4674), Mouse anti‐NeuN (Sigma, MAB377A5), Rabbit anti‐NeuN (Abcam, ab177487), Rat anti‐CD16 (BD Biosciences, 553 142), Rabbit anti‐iNOS (CST, 13120S), Rabbit anti‐COX‐2 (CST, 12282S), Rabbit anti‐TNF‐α (CST, 11948S), Rabbit anti‐HK2 (Abcam, ab209847), Rabbit anti‐Ki67 (Abcam, ab15580), Rabbit anti‐pH3 (Sigma, 0 6570), Rabbit anti‐MeCP2 (CST, 3456S), Rabbit anti‐Pan‐Kla (PTM, 1401RM), Mouse anti‐Arg1 (Santa Cruz Biotechnology, SC‐271430), Rabbit anti‐HIF‐1α (Abcam, ab179483), Rabbit anti‐LDHA (Abcam, ab52488), Rabbit anti‐IL‐10 (Abcam, ab9969), and Rabbit anti‐IL‐1 beta (Abcam, ab254360). The next day, sections were washed with PBS and incubated with fluorescently labeled secondary antibodies for 2 h at room temperature: Donkey anti‐rabbit Alexa Fluor 488 (Jackson), Donkey anti‐goat Alexa Fluor 594 (Jackson), Goat anti‐rabbit Alexa Fluor 647 (Jackson), Goat anti‐rat Alexa Fluor 594 (Jackson), Goat anti‐mouse Alexa Fluor 488 (Jackson), Goat anti‐chicken Alexa Fluor 594 (Jackson), Goat anti‐rabbit Alexa Fluor 488 (Jackson). Following secondary antibody incubation, DAPI (20 minutes) was used to counterstain nuclei. Stained sections were mounted with antifade mounting medium and visualized using an Olympus slide scanner (VS120‐L100) for whole‐brain imaging. High‐resolution images were captured using a Nikon confocal microscope (AX Ti2E, Japan) for detailed analysis. ImageJ software was used for image processing and quantification.

### Cell Culture and Treatment

BV2 murine microglial cells and HMC3 human microglial cells were obtained from Shanghai Fuheng Biotechnology Co. BV2 cells were maintained in DMEM (Dulbecco's Modified Eagle Medium), while HMC3 cells were cultured in MEM, both supplemented with 10% heat‐inactivated FBS, 100 U mL^−1^ penicillin, and 100 µg mL^−1^ streptomycin. Cells were passaged at 70%–80% confluence, with medium changed every 2–3 days. To induce neuroinflammatory activation, BV2 cells were treated with lipopolysaccharide (LPS, 100 ng mL^−1^) and interferon‐γ (IFN‐γ, 20 ng mL^−1^) for 24 h under standard culture conditions. HMC3 cells were exposed to a higher concentration of LPS (1 µg mL^−1^) and IFN‐γ (500 ng mL^−1^) for 24 h under the same conditions.

### Pharmacological Treatments

In vivo administration: Following tMCAO, mice received intraperitoneal injections of pharmacological agents once daily for three consecutive days. The treatment regimens included: Dichloroacetate (DCA), 200 mg kg^−1^ day^−1^ (Aladdin, China); Rotenone, 1.5 mg kg^−1^ day^−1^ (Aladdin, China); A‐485, 100 mg kg^−1^ day^−1^ (MedChemExpress, USA); Lonidamine (LND), 60 mg kg^−1^ day^−1^ (Selleck Chemicals, USA). In vitro administration: Rapamycin and AICAR were purchased from MedChem Express (MCE, USA). For BV2 and HMC3 cells, DCA (5 mm for BV2; 20 mm for HMC3) and Rotenone (10 mm for BV2; 50 mm for HMC3) were administered separately for 24 h. A‐485 (10 µm) and LND (20 µm) were applied to BV2 cells for 24 h. Rapamycin was administered at a final concentration of 37.5 nm, 12 h prior to LPS and IFN‐γ treatment; AICAR was administered at a final concentration of 100 µm, 12 h prior to LPS and IFN‐γ treatment.^[^ ^67^, ^68^ ^]^ At the end of the respective incubation period, cells were harvested for downstream molecular and cellular analyses.

### Preparation of Single‐Cell Suspension and Microglia Isolation

Single‐cell suspensions were generated from brain tissue through enzymatic dissociation and density gradient centrifugation. Animals were anesthetized with isoflurane and perfused with ice‐cold HBSS. Whole brains were dissected, minced, and enzymatically digested at 37 °C using a mechanical dissociator. The resulting homogenate was filtered through a 70‐µm mesh, centrifuged, and subjected to debris removal by density gradient centrifugation. The cell pellet was resuspended in HBSS with 1% FBS. For microglia isolation, the single‐cell suspension was incubated with FcR blocking buffer to reduce nonspecific activation, then labeled with anti‐CD11b microbeads and separated via magnetic sorting using an LS column. Purified microglia were stained with an anti‐CD11b‐APC antibody and analyzed by flow cytometry to determine purity.

### Quantitative Real‐Time PCR (qRT‐PCR)

Total RNA was extracted from cells and brain tissues using Trizol reagent (Life Technologies), following the manufacturer's instructions. RNA concentration and purity were assessed with a NanoDrop spectrophotometer, ensuring an A260/A280 ratio between 1.8 and 2.0. Complementary DNA (cDNA) synthesis was carried out using the HiScript III All‐in‐One RT SuperMix (Vazyme, R333). qRT‐PCR was performed on a QuantStudio 5 system (Applied Biosystems, Thermo Fisher Scientific) using ChamQ Universal SYBR qPCR Master Mix (Vazyme, Q711). Primer sequences are provided in Table S3 (Supporting Information). Gene expression was normalized to Gapdh, and relative expression levels were calculated using the 2^^−ΔΔCt^ method.

### Isolation of Primary Microglia

Primary microglia were isolated from postnatal (1–3 days old) C57BL/6 mice. After euthanasia, brains were dissected, minced, and digested with 0.25% trypsin at 37 °C for 30 min. The cell suspension was filtered through 70‐µm strainers to remove debris, then resuspended in F12/DMEM medium containing 20% FBS, 1% penicillin‐streptomycin, and 10 ng mL^−1^ recombinant macrophage colony‐stimulating factor (M‐CSF). Cells were seeded in T75 culture flasks and maintained for 14 days. Microglia were detached using a sequential trypsinization protocol: 0.0625% trypsin for 30 min, followed by 0.25% trypsin for 10 min at 37 °C. The identity and purity of isolated microglia were confirmed by immunostaining for Iba1 and TMEM119, two microglia‐specific markers.

### In Vitro Oxygen‐Glucose Deprivation Assay

To model ischemic injury in vitro, oxygen‐glucose deprivation (OGD) was performed on primary microglia, BV2, and HMC3 cells. Cells were seeded at appropriate densities and cultured in complete medium until reaching 70%–80% confluence. For OGD induction, the medium was replaced with glucose‐free DMEM (Gibco), and cells were placed in a hypoxia chamber maintained at 1% O_2_, 5% CO_2_, and 94% N_2_ at 37 °C. OGD was applied for 4 h. Control cells were incubated in glucose‐containing medium under normoxic conditions. After OGD, cultures were reoxygenated by returning them to normoxic conditions and replacing the medium with standard glucose‐containing DMEM to simulate reperfusion. Cells were collected at defined time points for subsequent analyses, including western blotting, and immunofluorescence staining. All experiments were conducted in parallel across cell types to ensure procedural consistency and data comparability.

### Western Blot Analysis

Proteins were extracted from tissues, cultured cells using RIPA lysis buffer. Protein concentrations were measured using the Bradford Protein Assay Kit. Equal amounts of denatured protein lysates were resolved by SDS‐PAGE and transferred onto PVDF membranes (Millipore). Membranes were blocked with appropriate blocking buffer and incubated overnight at 4 °C with the following primary antibodies: iNOS (CST, 13120S), COX‐2 (CST, 12282S), TNF‐α (CST, 11948S), HK2 (Abcam, ab209847), HIF‐1α (CST, 36169T), PKM2 (Proteintech, 15822‐1‐AP), MeCP2 (CST, 3456S), Pan‐Kla (PTM, 1401RM), P300 (CST, 57625S). After washing with TBST, membranes were incubated with HRP‐conjugated secondary antibodies for 2 h at room temperature. Protein bands were detected using an enhanced chemiluminescence (ECL) detection kit (Millipore, MA) and imaged using a chemiluminescence imaging system. Band intensities were quantified using ImageJ software. All western blot experiments were performed in triplicate to ensure reproducibility.

### Flow Cytometry Analysis

Protein expression analysis: Protein expression was assessed using flow cytometry. Cells were fixed in 4% paraformaldehyde (PFA) for 15 min at room temperature, permeabilized with 0.5% Triton X‐100 for 20 min, and blocked with 1% BSA in PBS for 10 min. After blocking, cells were incubated with primary antibodies for 1 h, followed by fluorophore‐conjugated secondary antibodies for 30 min at room temperature. Fluorescence data were acquired using a flow cytometer and analyzed with FlowJo software (v10.8.1). BV2 and HMC3 cell proliferation was measured using the BeyoClick EdU‐594 Cell Proliferation Kit (Beyotime, China). Cells were incubated with 10 µm EdU for 2 h, detached with 0.25% trypsin, washed twice with PBS, and resuspended in PBS before flow cytometry analysis. A negative control was used to establish the fluorescence threshold, and the percentage of EdU‐positive cells was quantified to determine proliferation rates. BV2 microglial cells were analyzed for intracellular calcium concentration, caspase‐3 activation, mitochondrial membrane potential (MMP), reactive oxygen species (ROS) levels, and glucose uptake using flow cytometry. Fluo‐4 AM (Beyotime, S1061) was used to assess intracellular calcium levels following a 30‐min incubation (*E*_x_/*E*_m_ = 490/525 nm). Caspase‐3 activation and MMP changes were detected using the Caspase‐3 Activity and MMP Detection Kit (Beyotime, C1073), where GreenNuc Caspase‐3 Substrate (*E*_x_/*E*_m_ = 500/530 nm) indicated caspase‐3 activation, and Mito‐Tracker Deep Red 633 (*E*_x_/*E*_m_ = 622/648 nm) measured MMP changes. Mitochondrial ROS levels were determined using MitoSOX Red (Beyotime, S0061), a mitochondrial superoxide indicator emitting red fluorescence upon oxidation (*E*_x_/*E*_m_ = 510/580 nm). Glucose uptake was measured using 2‐NBDG (Elabscience, E‐CK‐A441), a fluorescent glucose analog (*E*_x_/*E*_m_ = 475/550 nm). Cells were incubated with the respective probes under optimized conditions and analyzed using flow cytometry. Fluorescence intensity was quantified to compare differences between treatment and control groups.

### Transwell Migration Assay

BV2 or HMC3 cell migration was evaluated using Transwell inserts (8.0‐µm pore size, Corning, NY) in 24‐well plates, following the manufacturer's protocol. Cells were suspended in serum‐free medium and seeded into the upper chamber, while 500 µL of complete medium was added to the lower chamber. After 24 h of incubation, the lower chamber was supplemented with different treatments. Following an additional 24‐h incubation, nonmigrated cells were removed from the upper surface, while migrated cells on the lower membrane surface were fixed with 4% PFA (30 min) and stained with 0.25% crystal violet (Beyotime). Migrated cells were imaged and quantified.

### Lactate Quantification

Lactate levels in serum, brain tissue and cells were measured using the LA Content Assay Kit (Solarbio, BC2235). Equal amounts of samples from each experimental group were homogenized in extraction solutions A and B, followed by centrifugation to obtain the supernatant. The reaction solution and color‐developing solution were added to the collected supernatant, and absorbance was measured at 570 nm using a Varioskan LUX microplate reader (Bio‐Rad) to determine lactate concentrations.

### Lactate Uptake Assay

FITC‐labeled lactic acid (FITC‐Lactic acid), a fluorescent probe generated by conjugating lactic acid with fluorescein isothiocyanate (FITC), was purchased from Xi'an Qiyue Biology (China). To assess lactate uptake, primary microglia were incubated with FITC‐Lactic acid for 30, 60, or 120 min. After incubation, cells were gently rinsed with PBS to remove uninternalized probe. Intracellular fluorescence intensity—correlating with internalized FITC‐Lactic acid—was analyzed via immunofluorescence microscopy.

### Energy Metabolism Assays in BV2 Microglia

ATP levels, pyruvate dehydrogenase (PDH) activity, and pyruvate kinase (PK) activity were quantified in BV2 microglial cells using colorimetric assays. ATP content was measured with the ATP Assay Kit (Solarbio, BC0300), which detects NADPH absorption at 340 nm. PDH activity was assessed using the PDH Activity Assay Kit (Solarbio, BC0380) by monitoring 2,6‐DCPIP reduction at 605 nm. PK activity was evaluated with the PK Activity Assay Kit (Solarbio, BC0540) based on NADH oxidation at 340 nm. Cells were incubated with the respective reaction mixtures at 37 °C, and absorbance was recorded at specific time points. Enzyme activities were calculated from standard curves.

### Seahorse Assays

BV2 microglial cells were initially cultured at a density of 2 × 10⁵ cells mL^−1^ for 48 h, followed by reseeding at 3 × 10⁴ cells per well in a 24‐well Seahorse microplate. Cellular metabolic profiles were assessed using the Seahorse XF Glycolytic Rate Assay Kit (Cat. No. 103344‐100; Agilent Technologies, USA) and a Seahorse XF24 Extracellular Flux Analyzer. For measurement of the extracellular acidification rate (ECAR), rotenone/antimycin A (0.5 µm) and 2‐deoxy‐D‐glucose (2‐DG, 50 mm) were added to each well of the microplate. Acquired data were analyzed with Wave software (Agilent Technologies, USA).

### Immunoprecipitation (IP)

Cells samples were lysed in Lysis/Wash Buffer and centrifuged at 12 000 rpm, 4 °C for 5–10 min to collect the supernatant. 500 µg of total protein was incubated with 5 µg of primary antibody in a final volume of 500 µL at room temperature overnight at 4 °C to form the antigen‐antibody complex. Protein A/G magnetic beads (50 µL) were washed twice with Lysis/Wash Buffer, resuspended in 100 µL of Lysis/Wash Buffer, and added to the immune complex. The mixture was rotated at room temperature for 30 min. Beads were then separated using a magnetic rack, the supernatant was removed, and beads were washed three times with Lysis/Wash Buffer. Bound proteins were eluted by adding 1× SDS‐PAGE Sample Loading Buffer and subjected to denaturation at 95 °C for 5 min. The eluates were used for Western blot analysis.

### Lentiviral Infection and Plasmid Transfection

BV2 cells were seeded in 6‐well plates and cultured to ≈55% confluency prior to transduction or transfection. Lentiviral particles encoding FLAG‐tagged wild‐type MeCP2 (MeCP2 WT) or its K210R mutant (MeCP2 K210R) were obtained from Shanghai Genechem Co., Ltd. Cells were infected at a multiplicity of infection (MOI) of 30 for 24 h, following the manufacturer's instructions. After infection, the medium was replaced with fresh complete medium, and cells were maintained for subsequent assays. To overexpress HK2, BV2 cells were transfected with a plasmid encoding full‐length mouse HK2 (OE‐HK2) or an empty vector control (Vec) using Lipofectamine 3000 (Thermo Fisher Scientific). Transfections were performed at ≈70% confluency with 2.5 µg plasmid DNA per well. Cells were harvested 48 h post‐transfection, and HK2 expression levels were confirmed by Western blotting. For gene silencing, BV2 cells were transfected with siRNA targeting HK2 (si‐HK2) or a scrambled control (Scr) using Lipofectamine RNAiMAX (Thermo Fisher Scientific). siRNA was applied at a final concentration of 50 nm. Cells were collected 48 h later, and knockdown efficiency was validated by immunoblotting.

### Pan‐Kla‐Based Post‐Translational Modification (PTM) Enrichment

Cells were lysed and sonicated in lysis buffer using an ultrasonic processor. Proteins were digested twice with trypsin to generate peptides. The resulting tryptic peptides were incubated overnight at 4 °C with Pan‐Kla antibodies (PTM‐1404, PTM Bio) conjugated to prewashed beads, facilitating the enrichment of Kla‐modified peptides. The bound peptides were eluted with 0.1% trifluoroacetic acid (TFA), vacuum‐dried, and desalted using C18 ZipTips (Millipore) before subsequent analysis.

### LC‐MS/MS Analysis and Database Search

LC‐MS/MS analysis was performed by Jingjie PTM Biolabs (Hangzhou, China). Peptides were separated using a nanoElute HPLC system (Bruker Daltonics) and introduced into a timsTOF Pro mass spectrometer (Bruker Daltonics) via a capillary source. The MaxQuant search engine (v1.6.15.0) was used for MS/MS data analysis. Peptide relative quantification was determined by normalizing signal intensities across samples. Quantified Kla peptide ratios were further adjusted based on their respective protein expression levels.

### Luminex‐Based Cytokine Profiling in Mouse Brain Tissue

Cytokine levels in mouse brain homogenates were measured using a Luminex multiplex assay before and after 2‐DG treatment. Proinflammatory cytokines (TNF‐α, IL‐1β, IFN‐γ, CCL2, CCL3, IL‐12) and anti‐inflammatory cytokines (IL‐4, IL‐10, IL‐13) were quantified to evaluate the inflammatory response. Protein samples were centrifuged to remove particulates. The supernatant (50 µL) was incubated with fluorescently labeled magnetic beads conjugated to capture antibodies. Biotinylated detection antibodies were then added, followed by PE‐labeled streptavidin. Fluorescence signals were recorded using a Luminex 200 system, and cytokine concentrations were calculated based on standard curves.

### Cut&tag Assay

The cut&tag assay was conducted using the Hyperactive Universal CUT&Tag Assay Kit for Illumina (Vazyme, TD903‐01) according to the manufacturer's instructions. BV2 cells were collected and immobilized onto concanavalin A‐coated beads. Cells were permeabilized using digitonin and incubated with MeCP2 antibodies. After incubation, pA‐Tn5 transposase was added for target fragmentation. DNA was extracted, amplified, and purified following transposase activation and tagmentation to generate sequencing libraries. Libraries were analyzed using an Illumina NovaSeq platform (150PE mode).

### Single‐Cell RNA‐seq Data Reanalysis

Single‐cell RNA sequencing (scRNA‐seq) data from the GEO database (GSE197731) were reanalyzed to examine MeCP2 expression in microglia following ischemic stroke. Raw expression matrices were processed using Seurat for quality control. After normalization and identification of highly variable genes, PCA was performed for dimensionality reduction, followed by t‐SNE clustering to visualize distinct cell populations. Cell‐type distribution was compared between the tMCAO and sham groups and UMAP analysis was used to map MeCP2 expression across different cell types, confirming its predominant enrichment in microglia and monocytes. Finally, violin plot analysis revealed no significant difference in MeCP2 expression levels between the groups.

### Behavioral Assessments–Garcia Score

The Garcia score (maximum 18 points) was used to evaluate sensorimotor function and neurological deficits in tMCAO mice. To ensure unbiased assessments, evaluations were performed under blinded conditions. The scoring system comprised six functional domains: spontaneous activity, limb symmetry, climbing ability, body proprioception, vibrissae response, and lateral turning. Each parameter was rated on a 0–3 scale, with 0 indicating severe impairment and 3 representing normal function. Mice were acclimated to the testing environment before evaluation. Assessments were conducted at predefined time points postsurgery.

### Rotarod Test

Motor coordination and endurance were assessed using the Rotarod test. Mice were placed on a rotating drum set at an initial speed of 5 rpm, gradually accelerating to 40 rpm over 300 s. Latency to fall was recorded as a measure of motor function. Baseline data were collected one day before surgery, followed by postoperative assessments on days 1, 3, 5, and 7. Each time point consisted of three trials per mouse, and the mean latency across trials was used for analysis.

### Adhesive Touch and Removal Test

This test assessed sensory and motor impairments in the forepaw following tMCAO. A 3 × 4 mm adhesive tape was applied to either the ipsilateral or contralateral forepaw, depending on the area under evaluation. Two latency measures were recorded: 1) Time to detect and touch the adhesive. 2) Time to completely remove the tape. A 120‐s cutoff was set per trial. Baseline values were recorded 1 day before tMCAO, with postoperative measurements taken on days 1, 3, 5, and 7. Each session comprised three independent trials, and the mean latency for touch and removal was used for analysis.

### Grid‐Walking Test

The grid‐walking test was used to assess sensorimotor coordination and limb placement accuracy. Mice were placed on an elevated wire grid, and spontaneous movement was recorded for 5 min. A blinded investigator analyzed video recordings offline, quantifying the total number of steps taken and the number of foot faults, defined as paw slips through the grid. The foot‐fault rate was calculated as the percentage of foot faults relative to total steps and used as an indicator of motor impairment.

### Open Field Test

To evaluate spontaneous locomotor activity and anxiety‐like behavior following surgical intervention, mice were subjected to the open field test in a square arena (40 × 40 × 40 cm^3^). After a brief acclimatization period, each mouse was placed individually into the center of the arena and allowed to explore freely for 10 min. Behavioral tracking was conducted using an automated video‐based analysis system. Parameters quantified included total distance traveled, time spent in the center zone, and average movement velocity. The apparatus was sanitized with 70% ethanol between trials to eliminate olfactory cues.

### Statistical Analysis

Statistical analyses were performed using GraphPad Prism 10 (GraphPad Software, Inc., La Jolla, CA). For experiments using cell lines and technical replicates, data are presented as mean ± SD. For in vivo experiments, data are presented as mean ± SEM. Prior to parametric analysis, data distribution was evaluated using the Shapiro–Wilk test to confirm normality. For normally distributed data: Comparisons between two groups with homogeneous variances (confirmed via Levene's test) were performed using an unpaired two‐tailed Student's *t*‐test. Comparisons involving three or more groups with homogeneous variances (confirmed via Bartlett's test) were analyzed using one‐way or two‐way analysis of variance (ANOVA), followed by Tukey's post hoc test. If variance inequality was detected, Welch's *t*‐test (for two groups) or Welch's ANOVA (for three or more groups) was used instead. To minimize observer bias, experimenters were blinded to group assignments during sample processing, data acquisition, and quantification. Statistical significance was defined as *p* < 0.05, with significance levels denoted as follows: *p < 0.05, **p < 0.01, ***p < 0.001. Nonsignificant results are labeled “ns” where applicable.

### Ethical Statement

Animal experiments were conducted in accordance with the principles of the Declaration of Helsinki and approved by the Department of Laboratory Animal Science, Fudan University (Approval No. 2023‐MHFY‐23JZS). The study involving human participants was approved by the Ethics Committee of Minhang Hospital (Approval No. 2021‐008‐01K).

## Conflict of Interest

The authors declare no conflict of interest.

## Author Contributions

Z.Z., S.H., and Y.W. contributed equally to this work. Z.Z., S.H., and Y.W. made equal contributions to this work. They jointly designed and performed most of the experiments. Z.J. and Z.L. assisted in conducting in vitro experiments and contributed to data analysis. C.W. assisted in performing in vivo experiments. R.J. and Y.Y. assisted in analyzing CUT&Tag data. X.M., K.Y., and H.N. assisted with the analysis of immunoblotting and qPCR data. J.Z. and Y.G. conceptualized the study, supervised the experimental work, acquired funding. Y.G. and Z.Z. critically edited the manuscript. All authors have read and approved the final version of the manuscript.

## Supporting information



Supporting Information

Supplemental Table 1

Supporting Information

## Data Availability

The data that support the findings of this study are available from the corresponding author upon reasonable request.

## References

[1] X. Fan , J. Cao , M. Li , D. Zhang , I. El‐Battrawy , G. Chen , X. Zhou , G. Yang , I. Akin , Adv. Sci. 2024, 11, 2307698. 10.1002/advs.202307698 PMC11005719 38308187

[2] M. Liu , X. Zhou , Y. Li , S. Ma , L. Pan , X. Zhang , W. Zheng , Z. Wu , K. Wang , A. Ahsan , J. Wu , L. Jiang , Y. Lu , W. Hu , Z. Qin , Z. Chen , X. Zhang , Redox Biol. 2022, 53, 102323. 35576689 10.1016/j.redox.2022.102323 PMC9118922

[3] H. Jin , Z. Li , S. Tan , Q. Xiao , Q. Li , J. Ye , Y. Zhou , Y. Wan , Q. Liu , B. K. Menon , B. Hu , Adv. Sci. 2025, 12, 03722. 10.1002/advs.202503722 PMC12462934 40557450

[4] Z. Zhang , R. Ji , Z. Liu , Z. Jiang , M. Chu , Y. Wang , J. Zhao , J. Nanobiotechnol. 2025, 23, 572. 10.1186/s12951-025-03652-z PMC12363081 40830888

[5] J. Lv , Y. Jiao , X. Zhao , X. Kong , Y. Chen , L. Li , X. Chen , X. Tao , D. Dong , CNS Neurosci. Ther. 2025, 31, 70229. 10.1111/cns.70229 PMC11822359 39945118

[6] Z. Zhu , Z. Li , K. L. Lambertsen , Z. Cao , M. Chen , Y. Xu , B. H. Clausen , K. Jin , Y. Lu , F. Mei , X. Du , K. Chen , H. Bai , X. Su , B. Zhou , B. Liu , R. Li , C. Wang , Y. Li , X. Cai , D. Schlüter , W. Song , X. Wang , Adv. Sci. 2025, 12, 03972. 10.1002/advs.202503972 PMC12561430 40755418

[7] X. Deng , Z. Hu , S. Zhou , Y. Wu , M. Fu , C. Zhou , J. Sun , X. Gao , Y. Huang , CNS Neurosci. Ther. 2023, 30, 14510. 10.1111/cns.14510 PMC10805403 37905592

[8] D. Zhang , Z. Tang , H. Huang , G. Zhou , C. Cui , Y. Weng , W. Liu , S. Kim , S. Lee , M. Perez‐Neut , J. Ding , D. Czyz , R. Hu , Z. Ye , M. He , Y. G. Zheng , H. A. Shuman , L. Dai , B. Ren , R. G. Roeder , L. Becker , Y. Zhao , Nature 2019, 574, 575. 31645732 10.1038/s41586-019-1678-1 PMC6818755

[9] J. Zhou , L. Zhang , J. Peng , X. Zhang , F. Zhang , Y. Wu , A. Huang , F. Du , Y. Liao , Y. He , Y. Xie , L. Gu , C. Kuang , W. Ou , M. Xie , T. Tu , J. Pang , D. Zhang , K. Guo , Y. Feng , S. Yin , Y. Cao , T. Li , Y. Jiang , Cell Metab. 2024, 36, 2054. 38906140 10.1016/j.cmet.2024.05.016

[10] B. Chen , K. Jin , J. Dong , S. Cheng , L. Kong , S. Hu , Z. Chen , J. Lu , Adv. Sci. 2024, 11, 2405354. 10.1002/advs.202405354 PMC11481194 39119889

[11] Y. Wang , P. Li , Y. Xu , L. Feng , Y. Fang , G. Song , L. Xu , Z. Zhu , W. Wang , Q. Mei , M. Xie , J. Neuroinflammation 2024, 21, 308. 39609834 10.1186/s12974-024-03303-4 PMC11605911

[12] J. Wang , Z. Wang , Q. Wang , X. Li , Y. Guo , Cell. Mol. Biol. Lett. 2024, 29, 23. 38317138 10.1186/s11658-024-00541-5 PMC10845568

[13] F. Meng , J. He , X. Zhang , W. Lyu , R. Wei , S. Wang , Z. Du , H. Wang , J. Bi , X. Hua , C. Zhang , Y. Guan , G. Lyu , X.‐L. Tian , L. Zhang , W. Xie , W. Tao , Adv. Sci. 2025, 12, 2412747. 10.1002/advs.202412747 PMC12165025 40388671

[14] M. Sun , Y. Zhang , R. Mao , Y. Chen , P. Liu , L. Ye , S. Xu , J. Jia , S. Shu , H. Li , Y. Yin , S. Xia , Y. Chen , Y. Xu , Adv. Sci. 2025, 12, 2415309, 10.1002/advs.202415309. PMC12140320 40271828

[15] W.‐Y. Si , C.‐L. Yang , S.‐L. Wei , T. Du , L.‐K. Li , J. Dong , Y. Zhou , H. Li , P. Zhang , Q.‐J. Liu , R.‐S. Duan , R.‐N. Duan , Commun. Biol. 2024, 7, 1701. 39725685 10.1038/s42003-024-07425-6 PMC11671539

[16] W. Zhang , L. Xu , Z. Yu , M. Zhang , J. Liu , J. Zhou , Mol. Biotechnol. 2022, 65, 1336. 36574182 10.1007/s12033-022-00643-5 PMC10352161

[17] J. Li , C. Tang , X. Zhang , R. Xing , Q. Guo , Adv. Sci. 2025, 12, 03897, 10.1002/advs.202503897. PMC12667545 40990975

[18] Y. Zhang , H. Jiang , M. Dong , J. Min , X. He , Y. Tan , F. Liu , M. Chen , X. Chen , Q. Yin , L. Zheng , Y. Shao , X. Li , H. Chen , Cell Rep. 2024, 43, 114180. 38733581 10.1016/j.celrep.2024.114180

[19] J. Cai , P. Zhang , Y. Cai , G. Zhu , S. Chen , L. Song , J. Du , B. Wang , W. Dai , J. Zhou , J. Fan , Y. Yu , Z. Dai , Adv. Sci. 2025, 12, 2413095. 10.1002/advs.202413095 PMC12120759 40305758

[20] G. Wu , H. Cheng , J. Yin , Y. Zheng , H. Shi , B. Pan , M. Li , M. Zhao , J. Liang , Y. Bian , G. Shan , G. Bi , W. Guo , L. Wang , Y. Huang , Adv. Sci. 2025, 12, 01238. 10.1002/advs.202501238 PMC12412559 40539245

[21] G. Zhang , A. Zhao , X. Zhang , M. Zeng , H. Wei , X. Yan , J. Wang , X. Jiang , Y. Dai , Cell. Signalling 2024, 124, 111466. 39419195 10.1016/j.cellsig.2024.111466

[22] G. Yang , L. Zhang , Y. Yuan , M. Mazhar , D. Zhang , Y. Liu , G. Chen , X. Fan , CNS Neurosci. Ther. 2025, 31, 70567. 10.1111/cns.70567 PMC12365391 40831324

[23] J. C. Cronk , N. C. Derecki , E. Ji , Y. Xu , A. E. Lampano , I. Smirnov , W. Baker , G. T. Norris , I. Marin , N. Coddington , Y. Wolf , S. D. Turner , A. Aderem , A. L. Klibanov , T. H. Harris , S. Jung , V. Litvak , J. Kipnis , Immunity 2015, 42, 679. 25902482 10.1016/j.immuni.2015.03.013 PMC4407145

[24] R. Du , Y. Gao , C. Yan , X. Ren , S. Qi , G. Liu , X. Guo , X. Song , H. Wang , J. Rao , Y. Zang , M. Zheng , J. Li , H. Huang , IScience 2024, 27, 110911. 39351192 10.1016/j.isci.2024.110911 PMC11440250

[25] W. T. Ralvenius , A. E. Mungenast , H. Woolf , M. M. Huston , T. Z. Gillingham , S. K. Godin , J. Penney , H. P. Cam , F. Gao , C. G. Fernandez , B. Czako , Y. Lightfoot , W. J. Ray , A. Beckmann , A. M. Goate , E. Marcora , C. Romero‐Molina , P. Ayata , A. Schaefer , E. Gjoneska , L.‐H. Tsai , J. Exp. Med. 2023, 220, 20222105. 10.1084/jem.20222105 PMC10465325 37642942

[26] C. M. O'Driscoll , W. E. Kaufmann , J. P. Bressler , J. Neuroimmunol. 2013, 265, 61. 24268627 10.1016/j.jneuroim.2013.09.002

[27] L.‐W. Jin , M. Horiuchi , H. Wulff , X.‐B. Liu , G. A. Cortopassi , J. D. Erickson , I. Maezawa , J. Neurosci. 2015, 35, 2516. 25673846 10.1523/JNEUROSCI.2778-14.2015 PMC4323531

[28] Z. Cao , X. Min , X. Xie , M. Huang , Y. Liu , W. Sun , G. Xu , M. He , K. He , Y. Li , J. Yuan , Proc. Natl. Acad. Sci. USA 2024, 121, 2320383121. 10.1073/pnas.2320383121 PMC10861890 38289948

[29] E. Nance , S. P. Kambhampati , E. S. Smith , Z. Zhang , F. Zhang , S. Singh , M. V. Johnston , R. M. Kannan , M. E. Blue , S. Kannan , J. Neuroinflammation 2017, 14, 252. 29258545 10.1186/s12974-017-1004-5 PMC5735803

[30] E. S. Khoury , A. Sharma , R. R. Ramireddy , A. G. Thomas , J. Alt , A. Fowler , R. Rais , T. Tsukamoto , M. E. Blue , B. Slusher , S. Kannan , R. M. Kannan , Theranostics 2020, 10, 5736. 32483415 10.7150/thno.41714 PMC7254984

[31] D. P. Schafer , C. T. Heller , G. Gunner , M. Heller , C. Gordon , T. Hammond , Y. Wolf , S. Jung , B. Stevens , ELife 2016, 5, 15224, 10.7554/eLife.15224. PMC4961457 27458802

[32] U. Kahanovitch , K. C. Patterson , R. Hernandez , M. L. Olsen , Int. J. Mol. Sci. 2019, 20, 3813, 10.3390/ijms20153813. 31387202 PMC6696322

[33] P. Mesci , C. N. LaRock , J. J. Jeziorski , H. Nakashima , N. Chermont , A. Ferrasa , R. H. Herai , T. Ozaki , A. Saleh , C. E. Snethlage , S. Sanchez , G. Goldberg , C. A. Trujillo , K. Nakashima , V. Nizet , A. R. Muotri , Stem Cell Rep. 2024, 19, 1074. 10.1016/j.stemcr.2024.06.013 PMC11368698 39059378

[34] B. Cotto , H. Li , R. F. Tuma , S. J. Ward , D. Langford , Neurobiol. Dis. 2018, 117, 28. 29859319 10.1016/j.nbd.2018.05.017 PMC6051925

[35] R. Tang , Q.‐Q. Cao , S.‐W. Hu , L.‐J. He , P.‐F. Du , G. Chen , R. Fu , F. Xiao , Y.‐R. Sun , J.‐C. Zhang , Q. Qi , Acta Pharmacol. Sin. 2022, 43, 829. 34272506 10.1038/s41401-021-00727-z PMC8976037

[36] P. Periyasamy , A. Thangaraj , M.‐L. Guo , G. Hu , S. Callen , S. Buch , J. Neurosci. 2018, 38, 5367. 29760177 10.1523/JNEUROSCI.3474-17.2018 PMC5990983

[37] E. S. Khoury , R. V. Patel , C. O'Ferrall , A. Fowler , N. Sah , A. Sharma , S. Gupta , S. Scafidi , J. S. Kurtz , S. J. Olmstead , S. R. Kudchadkar , R. M. Kannan , M. E. Blue , S. Kannan , J. Neurochem. 2024, 168, 841. 37777475 10.1111/jnc.15960 PMC11002961

[38] L. Chen , M. Zhang , X. Yang , Y. Wang , T. Huang , X. Li , Y. Ban , Q. Li , Q. Yang , Y. Zhang , Y. Zheng , D. Wang , X. Wang , X. Shi , M. Zhang , Y. Sun , J. Wu , Theranostics 2024, 14, 4256. 39113793 10.7150/thno.94738 PMC11303070

[39] Y. Wang , L. Chen , M. Zhang , X. Li , X. Yang , T. Huang , Y. Ban , Y. Li , Q. Li , Y. Zheng , Y. Sun , J. Wu , B. Yu , Atherosclerosis 2023, 375, 45. 37245426 10.1016/j.atherosclerosis.2023.05.009

[40] L. Leng , Z. Yuan , R. Pan , X. Su , H. Wang , J. Xue , K. Zhuang , J. Gao , Z. Chen , H. Lin , W. Xie , H. Li , Z. Chen , K. Ren , X. Zhang , W. Wang , Z.‐B. Jin , S. Wu , X. Wang , Z. Yuan , H. Xu , H.‐M. Chow , J. Zhang , Nat. Metab. 2022, 4, 1287. 36203054 10.1038/s42255-022-00643-4

[41] Y. Li , B. Lu , L. Sheng , Z. Zhu , H. Sun , Y. Zhou , Y. Yang , D. Xue , W. Chen , X. Tian , Y. Du , M. Yan , W. Zhu , F. Xing , K. Li , S. Lin , P. Qiu , X. Su , Y. Huang , G. Yan , W. Yin , J. Neurochem. 2018, 144, 186. 29205357 10.1111/jnc.14267

[42] Y. Li , H. Zhou , X. He , L. Jin , Y. Zhu , L. Hu , M. Feng , J. Zhu , L. Wang , Y. Zheng , S. Li , Z. Yan , P. Cen , J. Hu , Z. Chen , X. Yu , X. Fu , C. Xu , S. Cao , Y. Cao , G. Chen , L. Wang , J. Adv. Res. 2024, 73, 575. 39142439 10.1016/j.jare.2024.08.016 PMC12225926

[43] S. Wang , C. Jiang , K. Cao , R. Li , Z. Gao , Y. Wang , Glia 2024, 72, 396. 37909251 10.1002/glia.24482

[44] J. F. Codocedo , C. Mera‐Reina , P. Bor‐Chian Lin , P. B. Fallen , S. S. Puntambekar , B. T. Casali , N. Jury‐Garfe , P. Martinez , C. A. Lasagna‐Reeves , G. E. Landreth , Cell Rep. 2024, 43, 114488. 39002124 10.1016/j.celrep.2024.114488 PMC11398604

[45] Y. Hu , K. Cao , F. Wang , W. Wu , W. Mai , L. Qiu , Y. Luo , W.‐P. Ge , B. Sun , L. Shi , J. Zhu , J. Zhang , Z. Wu , Y. Xie , S. Duan , Z. Gao , Nat. Metab. 2022, 4, 1756. 36536134 10.1038/s42255-022-00707-5

[46] W. Zhang , G. Feng , L. Wang , F. Teng , L. Wang , W. Li , Y. Zhang , Q. Zhou , J. Mol. Cell Biol. 2018, 10, 515. 29562294 10.1093/jmcb/mjy018

[47] R.‐Y. Pan , L. He , J. Zhang , X. Liu , Y. Liao , J. Gao , Y. Liao , Y. Yan , Q. Li , X. Zhou , J. Cheng , Q. Xing , F. Guan , J. Zhang , L. Sun , Z. Yuan , Cell Metab. 2022, 34, 634. 35303422 10.1016/j.cmet.2022.02.013

[48] L. Wei , X. Yang , J. Wang , Z. Wang , Q. Wang , Y. Ding , A. Yu , J. Neuroinflammation 2023, 20, 208. 37697347 10.1186/s12974-023-02879-7 PMC10494370

[49] A. De Leo , A. Ugolini , X. Yu , F. Scirocchi , D. Scocozza , B. Peixoto , A. Pace , L. D'Angelo , J. K. C. Liu , A. B. Etame , A. Rughetti , M. Nuti , A. Santoro , M. A. Vogelbaum , J. R. Conejo‐Garcia , P. C. Rodriguez , F. Veglia , Immunity 2024, 57, 1105. 38703775 10.1016/j.immuni.2024.04.006 PMC11114377

[50] X. Chen , Y. Wang , J.‐N. Wang , Y.‐C. Zhang , Y.‐R. Zhang , R.‐X. Sun , B. Qin , Y.‐X. Dai , H.‐J. Zhu , J.‐X. Zhao , W.‐W. Zhang , J.‐D. Ji , S.‐T. Yuan , Q.‐D. Shen , Q.‐H. Liu , EMBO Mol. Med. 2024, 16, 294. 38297099 10.1038/s44321-024-00025-1 PMC10897304

[51] Y. Zhang , J. Li , Y. Zhao , Y. Huang , Z. Shi , H. Wang , H. Cao , C. Wang , Y. Wang , D. Chen , S. Chen , S. Meng , Y. Wang , Y. Zhu , Y. Jiang , Y. Gong , Y. Gao , Sci. Adv. 2024, 10, ade6900. 10.1126/sciadv.ade6900 PMC10917353 38446877

[52] Z. Zhang , D. Ru , Z. Liu , Z. Guo , L. Zhu , Y. Zhang , M. Chu , Y. Wang , J. Zhao , MedComm 2025, 6, 70157. 10.1002/mco2.70157 PMC11999893 40242160

[53] C. Chen , Y. Zhou , X. Ning , S. Li , D. Xue , C. Wei , Z. Zhu , L. Sheng , B. Lu , Y. Li , X. Ye , Y. Fu , C. Bai , W. Cai , Y. Ding , S. Lin , G. Yan , Y. Huang , W. Yin , J. Neuroinflammation 2022, 19, 315. 36577999 10.1186/s12974-022-02682-w PMC9798610

[54] Y. Huang , G. Sun , X. Sun , F. Li , L. Zhao , R. Zhong , Y. Peng , Cancers 2020, 12, 3332, 10.3390/cancers12113332. 33187214 PMC7696079

[55] M. Ma , J. Wang , K. Guo , W. Zhong , Y. Cheng , L. Lin , Y. Zhao , A. Self‐Reinforced , Angew. Chem. Int. Ed 2025, 64, 202420547. 10.1002/anie.202420547 39714451

[56] Y. Ye , K. Ren , Y. Dong , L. Yang , D. Zhang , Z. Yuan , N. Ma , Y. Song , X. Huang , H. Qiao , ACS Appl. Mater. Interfaces 2023, 15, 26285. 37220137 10.1021/acsami.3c01559

[57] J. Lu , R. Li , B. Mu , Y. Peng , Y. Zhao , Y. Shi , L. Guo , L. Hai , Y. Wu , Eur. J. Med. Chem. 2021, 230, 114093. 35007860 10.1016/j.ejmech.2021.114093

[58] L. M. Lasko , C. G. Jakob , R. P. Edalji , W. Qiu , D. Montgomery , E. L. Digiammarino , T. M. Hansen , R. M. Risi , R. Frey , V. Manaves , B. Shaw , M. Algire , P. Hessler , L. T. Lam , T. Uziel , E. Faivre , D. Ferguson , F. G. Buchanan , R. L. Martin , M. Torrent , G. G. Chiang , K. Karukurichi , J. W. Langston , B. T. Weinert , C. Choudhary , P. de Vries , A. F. Kluge , M. A. Patane , J. H. Van Drie , C. Wang , et al., Nature 2017, 550, 128. 28953875 10.1038/nature24028 PMC6050590

[59] A. Mastracchio , C. Lai , E. Digiammarino , D. B. Ready , L. M. Lasko , K. D. Bromberg , W. J. McClellan , D. Montgomery , V. Manaves , B. Shaw , M. Algire , M. J. Patterson , C. C. Sun , S. Rosenberg , A. Lai , M. R. Michaelides , ACS Med. Chem. Lett. 2021, 12, 726. 34055218 10.1021/acsmedchemlett.0c00654 PMC8155265

[60] P. Gou , W. Zhang , Biomed. Pharmacother. 2024, 171, 116130. 38215693 10.1016/j.biopha.2024.116130

[61] J. White , F. A. Derheimer , K. Jensen‐Pergakes , S. O'Connell , S. Sharma , N. Spiegel , T. A. Paul , Trends Pharmacol. Sci. 2024, 45, 243. 38383216 10.1016/j.tips.2024.01.010

[62] L. Nicosia , G. J. Spencer , N. Brooks , F. M. R. Amaral , N. J. Basma , J. A. Chadwick , B. Revell , B. Wingelhofer , A. Maiques‐Diaz , O. Sinclair , F. Camera , F. Ciceri , D. H. Wiseman , N. Pegg , W. West , T. Knurowski , K. Frese , K. Clegg , V. L Campbell , J. Cavet , M. Copland , E. Searle , T. C. P. Somervaille , Cancer Cell 2023, 41, 2126. 10.1016/j.ccell.2023.11.001 37995682

[63] L. K. Xu , P. Ntziachristos , Cell Chem. Biol. 2023, 30, 1505. 38134880 10.1016/j.chembiol.2023.11.014

[64] Y. Yan , J. Ma , D. Wang , D. Lin , X. Pang , S. Wang , Y. Zhao , L. Shi , H. Xue , Y. Pan , J. Zhang , C. Wahlestedt , F. J. Giles , Y. Chen , M. E. Gleave , C. C. Collins , D. Ye , Y. Wang , H. Huang , EMBO Mol. Med. 2019, 11, 10659. 10.15252/emmm.201910659 PMC6835201 31559706

[65] F. Spriano , E. Gaudio , L. Cascione , C. Tarantelli , F. Melle , G. Motta , V. Priebe , A. Rinaldi , G. Golino , A. A. Mensah , L. Aresu , E. Zucca , S. Pileri , M. Witcher , B. Brown , C. Wahlestedt , F. Giles , A. Stathis , F. Bertoni , Blood Adv. 2020, 4, 4124. 32882003 10.1182/bloodadvances.2020001879 PMC7479962

[66] N. P. Jaschke , D. Breining , M. Hofmann , S. Pählig , U. Baschant , R. Oertel , S. Traikov , T. Grinenko , F. Saettini , A. Biondi , M. Stylianou , H. Bringmann , C. Zhang , T. M. Yoshida , H. Weidner , W. C. Poller , F. K. Swirski , A. Göbel , L. C. Hofbauer , M. Rauner , C. Scheiermann , A. Wang , T. D. Rachner , Immunity 2024, 57, 364. 38301651 10.1016/j.immuni.2024.01.005 PMC10923082

[67] K. Yao , Q. Mou , X. Lou , M. Ye , B. Zhao , Y. Hu , J. Luo , H. Zhang , X. Li , Y. Zhao , J. Neuroinflammation 2023, 20, 202. 37670386 10.1186/s12974-023-02886-8 PMC10481494

[68] L. Wan , R.‐M. Jia , L.‐L. Ji , X.‐M. Qin , L. Hu , F. Hu , Y. Han , Y.‐B. Pan , C.‐Y. Jiang , W.‐T. Liu , J. Neuroinflammation 2022, 19, 25. 35093117 10.1186/s12974-022-02384-3 PMC8800317

