# Supplementary material for: Flipping the Switch: MeCP2‐Mediated Lactylation Rewires Microglial Metabolism and Inflammation via the HK2/mTOR Axis in Poststroke Neuroinflammation
Source: Adv Sci (Weinh). 2025 Dec 22;13(12):e13400. doi: 10.1002/advs.202513400 (PMC12948279; doi:10.1002/advs.202513400)
Supplement: Supplementary file 1 — Supporting Information [file ADVS-13-e13400-s003.pdf]

Supporting Information

Flipping the Switch: MeCP2-Mediated Lactylation Rewires Microglial Metabolism and Inflammation via the HK2/mTOR Axis in Post-Stroke Neuroinflammation

Zengyu Zhang[1#], Shanshan Huang[2#], Yong Wang[3#], Zhiwen Jiang[4], Zhuohang Liu[1], Chenran Wang[1], Rong Ji[1], Yiwen Yuan[1], Xueyu Mao[1], Kaicheng Yang[1], Huicong Niu[1], Yanqin Gao[1*], Jing Zhao[1,5*]

[1]Department of Neurology of Minhang Hospital, State Key Laboratory of Brain Function and Disorders, MOE Frontiers Center for Brain Science, and Institutes of Brain Science, Fudan University, Shanghai 200032, China;

[2]Department of Endocrinology, Affiliated Hospital of Jiangnan University, Jiangnan University, Wuxi 214122, China;

[3]Department of Neurology, Zhongshan Hospital, Fudan University, Shanghai 200032, China;

[4]Department of Neurosurgery of Huashan Hospital, State Key Laboratory of Brain Function and Disorders, MOE Frontiers Center for Brain Science, and Institutes of Brain Science, Fudan University, Shanghai 200032, China;

[5]Institute of Healthy Yangtze River Delta, Shanghai Jiao Tong University, Shanghai 200030, China

#The authors contributed equally to this study.

*Corresponding authors:

Jing Zhao (zhao_jing@fudan.edu.cn); Yanqin Gao (yqgao@shmu.edu.cn)

Table S1. (See Table S1 in Supporting Information: Baseline Characteristics of 789 participants.xlsx).

Table S2. Baseline characteristics and comparison between favorable and unfavorable outcome groups. Table S2 presents the baseline characteristics of 789 participants, categorized into favorable (n = 525) and unfavorable (n = 264) outcome groups. Statistical analysis included t-tests for continuous variables and chi-square tests for categorical variables. Significant differences were found in age, NIHSS, fasting plasma glucose (FPG), and lactate dehydrogenase (LDH) levels. Notably, LDH levels were higher in the unfavorable group, suggesting a potential association with poor outcomes.

Variables	Total (n = 789)	Favorable (n = 525)	Unfavorable (n = 264)	Statistic	*P*
Age, Mean ± SD	68.77 ± 12.72	66.83 ± 12.61	72.62 ± 12.06	t=-6.18	<.001
NIHSS, Mean ± SD	9.20 ± 7.11	7.15 ± 5.32	13.29 ± 8.35	t=-10.89	<.001
FPG, Mean ± SD	7.00 ± 3.34	6.39 ± 2.63	8.51 ± 4.29	t=-6.61	<.001
LDH, Mean ± SD	177.20 ± 42.19	165.14 ± 26.73	201.16 ± 55.17	t=-10.03	<.001
Sex, n(%)				χ^2=6.07	0.014
Yes	530 (67.17)	368 (70.10)	162 (61.36)		
No	259 (32.83)	157 (29.90)	102 (38.64)		
Drinking, n(%)				χ^2=3.35	0.067
Yes	92 (11.66)	69 (13.14)	23 (8.71)		
No	697 (88.34)	456 (86.86)	241 (91.29)		
Smoking, n(%)				χ^2=8.83	0.003
Yes	233 (29.53)	173 (32.95)	60 (22.73)		
No	556 (70.47)	352 (67.05)	204 (77.27)		
Hypertension, n(%)				χ^2=4.25	0.039
Yes	471 (59.70)	300 (57.14)	171 (64.77)		
No	318 (40.30)	225 (42.86)	93 (35.23)		
Diabetes, n(%)				χ^2=10.83	<.001
Yes	214 (27.12)	123 (23.43)	91 (34.47)		
No	575 (72.88)	402 (76.57)	173 (65.53)		
Dyslipidemia, n(%)				χ^2=0.33	0.568
Yes	10 (1.27)	8 (1.52)	2 (0.76)		
No	779 (98.73)	517 (98.48)	262 (99.24)		

Abbreviations: t, t-test; χ^2, Chi-square test; SD, standard deviation.

Table S3. List of primer sequences used for qRT-PCR analysis.

Gene	Forward Sequence	Reverse Sequence
Gapdh	5'-AATGTGTCCGTCGTGGATCTGA-3'	5'-GATGCCTGCTTCACCACCTTCT-3'
Tnf	5'-GGTGCCTATGTCTCAGCCTCTT-3'	5'-GCCATAGAACTGATGAGAGGGAG-3'
Ptgs2	5'-GCGACATACTCAAGCAGGAGCA-3'	5'-AGTGGTAACCGCTCAGGTGTTG-3'
Nos2	5'-GAGACAGGGAAGTCTGAAGCAC-3'	5'-CCAGCAGTAGTTGCTCCTCTTC-3'
Il1b	5'-TGGACCTTCCAGGATGAGGACA-3'	5'-GTTCATCTCGGAGCCTGTAGTG-3'
Il6	5'-TACCACTTCACAAGTCGGAGGC-3'	5'-CTGCAAGTGCATCATCGTTGTTC-3'
Ccl2	5'-GCTACAAGAGGATCACCAGCAG-3'	5'-GTCTGGACCCATTCCTTCTTGG-3'
Ccl3	5'-ACTGCCTGCTGCTTCTCCTACA-3'	5'-ATGACACCTGGCTGGGAGCAAA-3'
Ccl5	5'-CCTGCTGCTTTGCCTACCTCTC-3'	5'-ACACACTTGGCGGTTCCTTCGA-3'
Cxcl10	5'-ATCATCCCTGCGAGCCTATCCT-3'	5'-GACCTTTTTTGGCTAAACGCTTTC-3'
Il12b	5'-TTGAACTGGCGTTGGAAGCACG-3'	5'-CCACCTGTGAGTTCTTCAAAGGC-3'
Hk2	5'-CCCTGTGAAGATGTTGCCCACT-3'	5'-CCTTCGCTTGCCATTACGCACG-3'
Ldha	5'-ACGCAGACAAGGAGCAGTGGAA-3'	5'-ATGCTCTCAGCCAAGTCTGCCA-3'
Pfkfb1	5'-AGAGGCAGTGAGCTACAGGAAC-3'	5'-TGACCTTCCTCACGGCTGAGAT-3'
Hif1α	5'-CCTGCACTGAATCAAGAGGTTGC-3'	5'-CCATCAGAAGGACTTGCTGGCT-3'
Slc16a1	5'-GACCATTGTGGAATGCTGCCCT-3'	5'-CGATGATGAGGATCACGCCACA-3'

Supplementary Figures

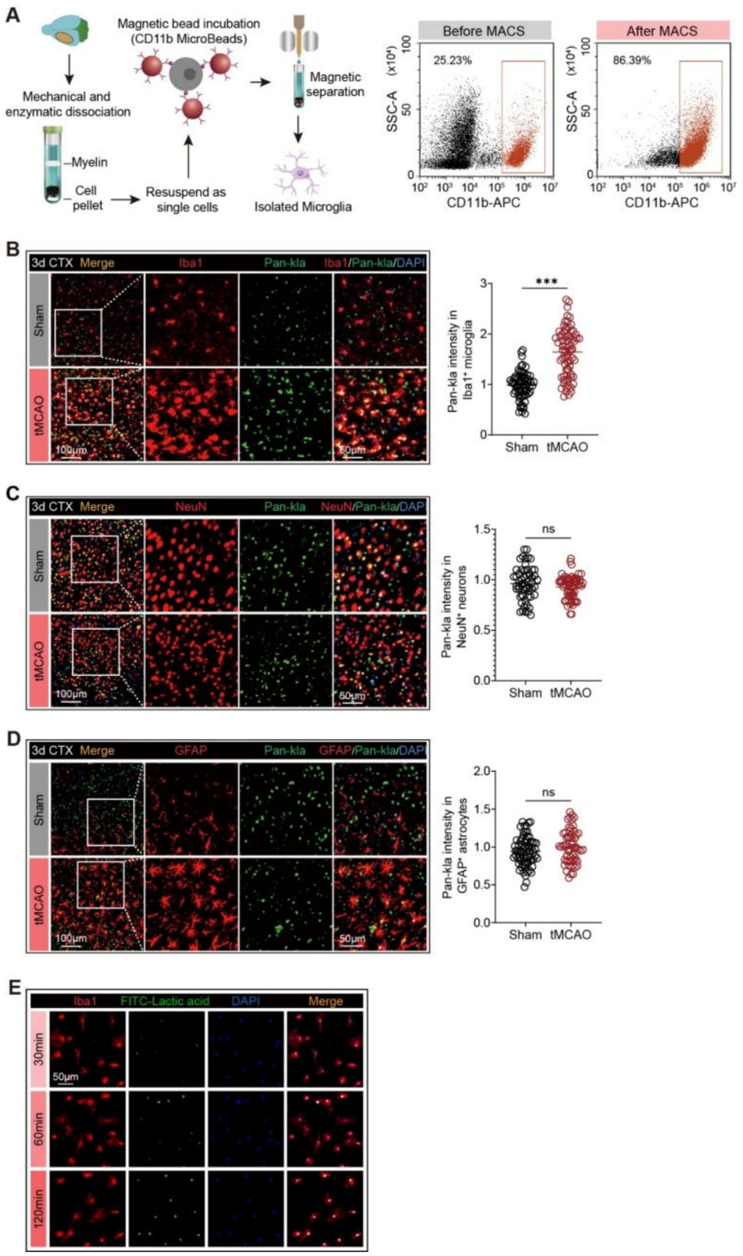

Fig. S1 Lactylation in brain cells and microglia isolation workflow in ischemic stroke. A Microglial isolation workflow. Purity check on flow cytometry using anti-CD11b-FITC on purified microglia. (Left) "Before MACS", single-cell suspension from brain homogenate; (Right) "After MACS", "CD11b (+)" and "CD11b (-)" cells after CD11b-MicroBeads selection. **B-D** Representative images of Pan-Kla co-staining with the microglial marker Iba1, the neuronal marker NeuN, or the astrocytic marker GFAP in the peri-infarct cortex. Pan-Kla intensity was compared between Sham and tMCAO groups. n > 50 cells per group. Data are presented as mean ± SEM. n.s.= not significant, ***$p < 0.001$. **E** Immunofluorescence staining of Iba1 and FITC-labeled lactic acid in primary microglia at 30 min, 60 min, and 120 min after FITC-lactic acid incubation.

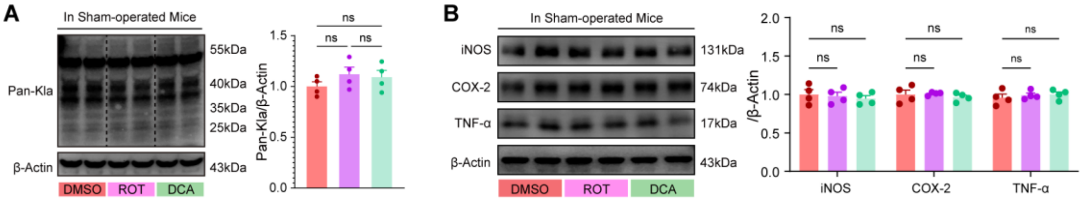

Fig. S2 Metabolic modulators do not affect basal protein lactylation or inflammatory marker expression in sham-operated mice. A Western blot analysis of Pan-Kla in the cortex of sham-operated mice treated with DMSO (vehicle), rotenone (ROT), or dichloroacetate (DCA). Quantification of Pan-Kla/β-Actin ratio showed no significant difference among treatment groups. n = 4 per group. **B** Immunoblot analysis of inflammatory markers in brain tissue of sham-operated mice following treatment with DMSO, ROT, or DCA. Quantification showed no significant changes in expression levels across groups. n = 4 per group. Data are presented as mean ± SEM. ns = not significant.

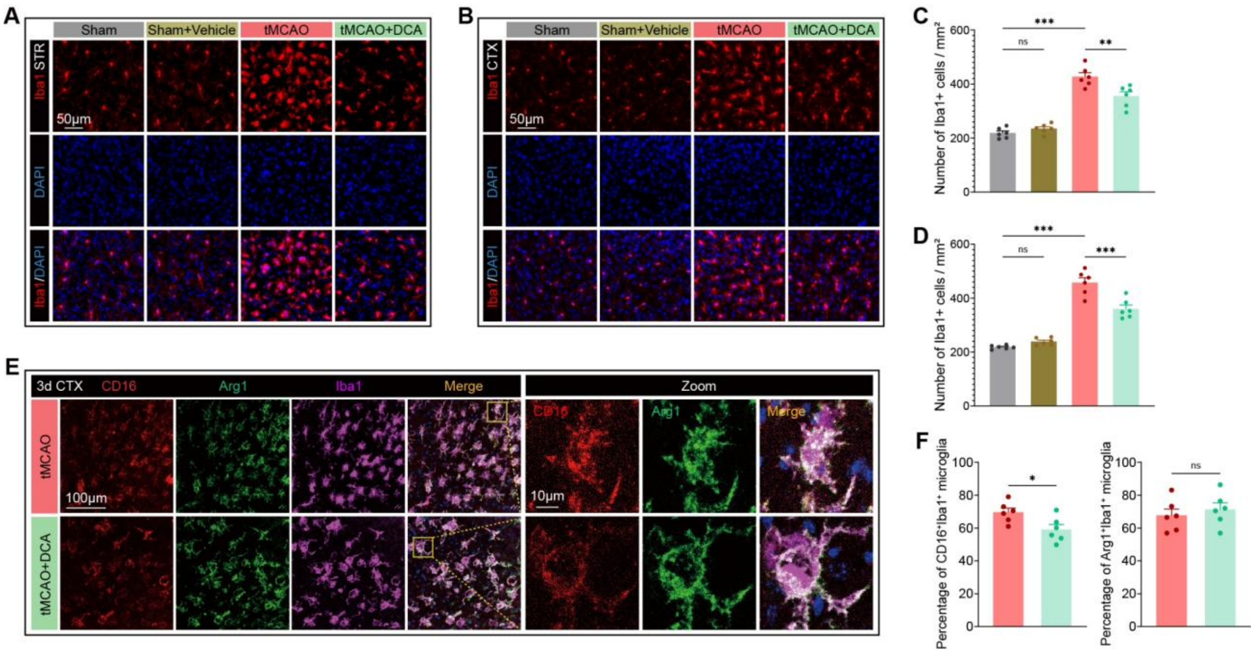

Fig. S3 DCA reduces microglial activation and suppresses pro-inflammatory marker expression after ischemic stroke. A, C Representative images and quantification of $Iba1^+$ microglia in the peri-infarct striatum (STR) at day 3 post-tMCAO. **B, D** Representative images and quantification of $Iba1^+$ microglia in the peri-infarct cortex (CTX) at day 3 post-stroke. **E** Immunofluorescent co-staining for CD16, Arg1, and Iba1 in the peri-infarct cortex reveals that DCA reduces CD16 expression in microglia, with minimal effect on Arg1. **F** Quantification of the ratio of $CD16^+Iba1^+$ and $Arg1^+Iba1^+$ cells. Data are presented as mean ± SEM, ns = not significant, *$p < 0.05$, **$p < 0.01$, ***$p < 0.001$.

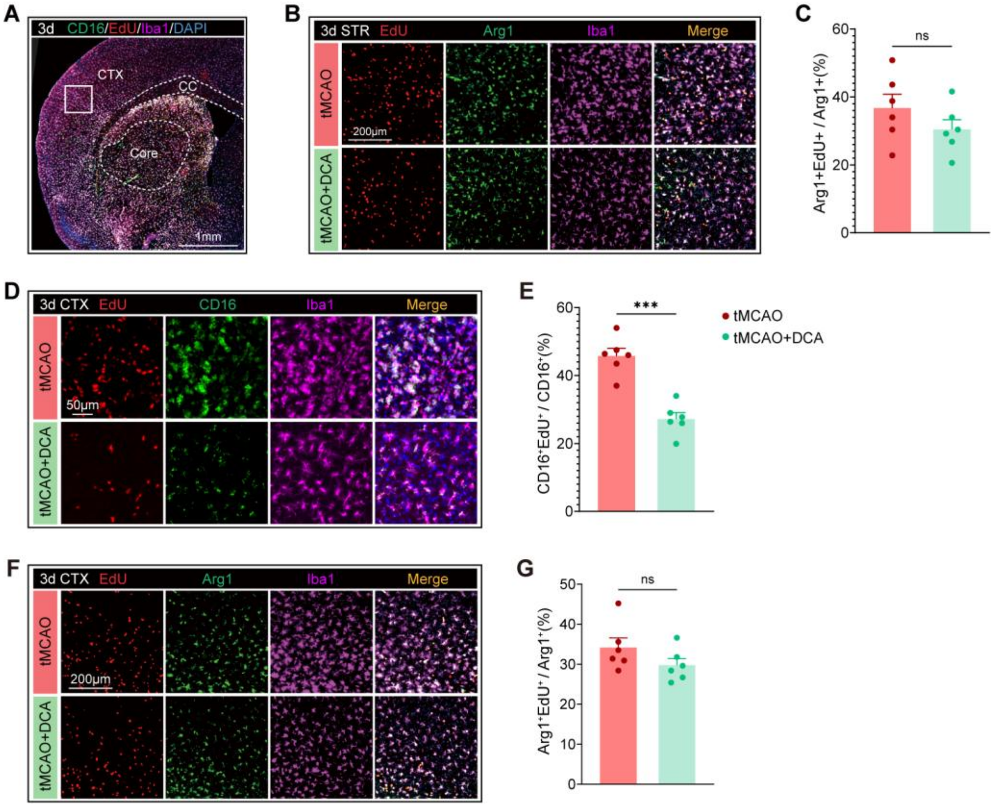

Fig. S4 DCA suppresses microglial proliferation and pro-inflammatory expansion in the ischemic brain. **A** Whole-coronal section at 3 days post-tMCAO showing EdU, CD16, Iba1, and DAPI in the peri-infarct cortex (CTX) and striatum (STR). **B–C** Representative images and quantification of EdU incorporation in Iba1$^+$ microglia and Arg1$^+$EdU$^+$ ratio in the STR. n = 6 per group. **D–E** Cortical microglia stained for EdU, CD16, and Iba1. DCA significantly decreased EdU incorporation and reduced the proportion of proliferating CD16$^+$ microglia. n = 6 per group. **F–G** Arg1$^+$ microglial proliferation in the CTX was unchanged by DCA. n = 6 per group. Data are presented as mean ± SEM, ns = not significant, ***$p < 0.001$.

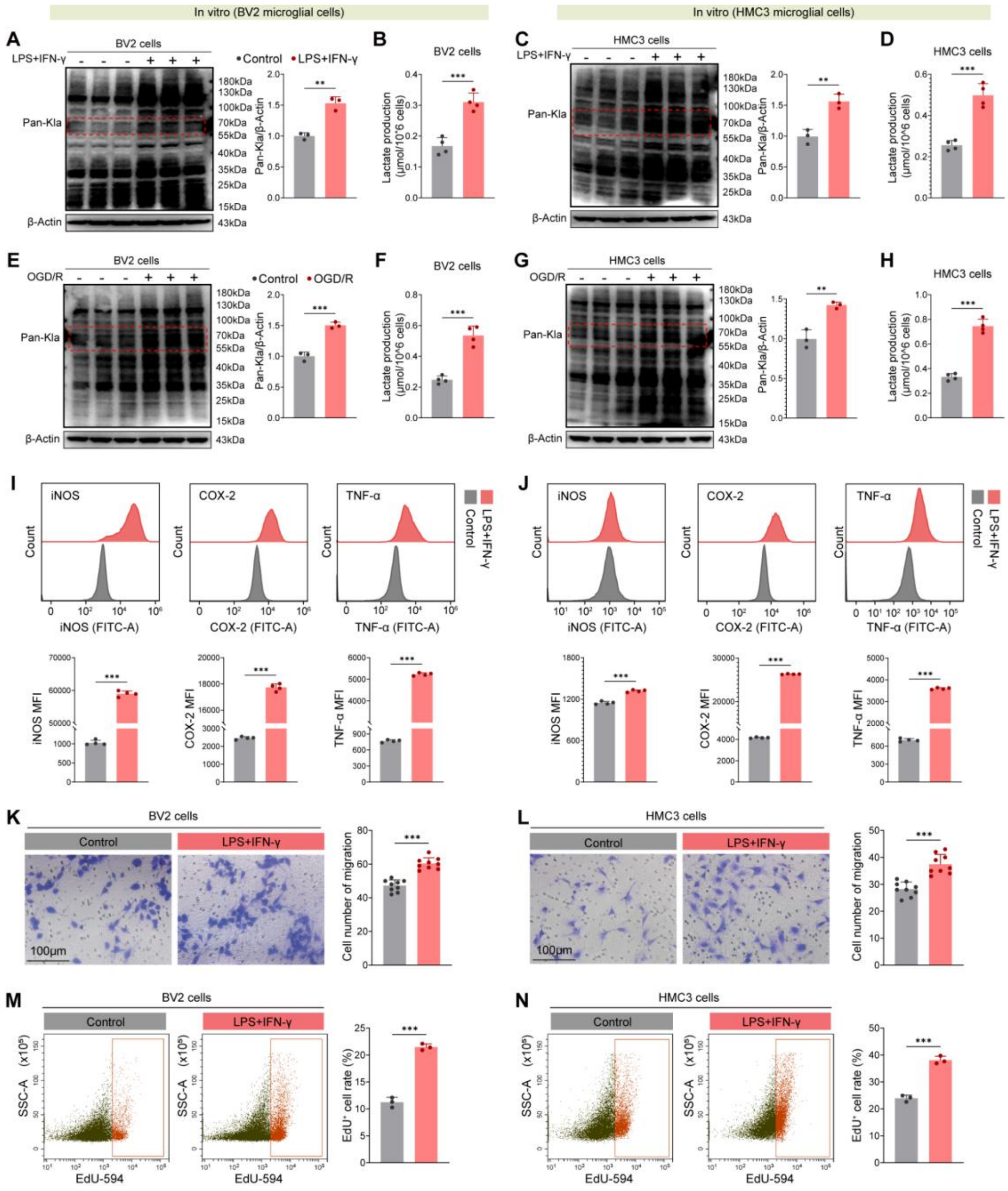

Fig. S5 Inflammatory and metabolic stress induce lactylation-dependent microglial activation, migration, and proliferation. A–B Western blot of Pan-Kla and quantification of lactate production in BV2 cells following LPS+IFN-γ stimulation. n = 3 or 4 per group. **C–D** Pan-Kla and lactate levels in HMC3 cells under LPS+IFN-γ stimulation. **E–F** Lactylation and lactate levels in BV2 cells under oxygen-glucose deprivation/reoxygenation (OGD/R). **G–H** Lactylation and lactate levels in HMC3 cells following OGD/R. **I–J** Flow cytometry analysis of iNOS, COX-2, and TNF-α expression in BV2 and HMC3 cells under LPS+IFN-γ stimulation. **K–L** Transwell migration assay of BV2 and HMC3 cells under inflammatory conditions. n = 9 per group. **M–N** EdU incorporation assays showing increased proliferation in BV2 and HMC3 cells following LPS+IFN-γ stimulation. n = 3 or 4 per group. Data are presented as mean ± SD. ***p*<0.01, ****p*<0.001.

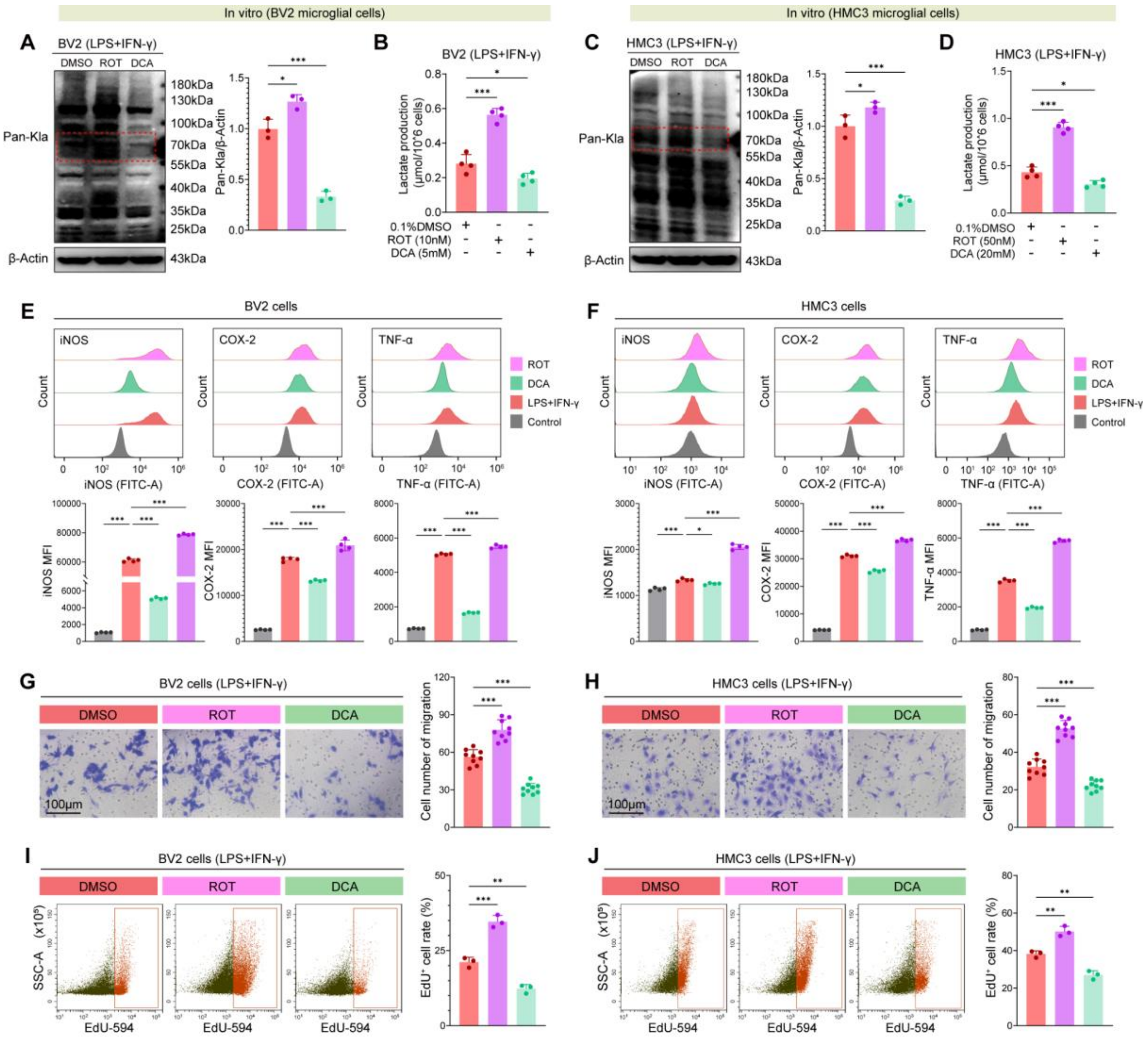

Fig. S6 Pharmacological modulation of lactylation regulates microglial inflammation, migration, and proliferation. A–B Western blot analysis of Pan-Kla and corresponding lactate production in BV2 cells treated with LPS+IFN-γ in the presence of DMSO, rotenone (ROT), or dichloroacetate (DCA). n = 3 or 4 per group. **C–D** Western blot analysis of Pan-Kla and lactate production in human HMC3 microglial cells. n = 3 or 4 per group. **E–F** Flow cytometry of iNOS, COX-2, and TNF-α in BV2 and HMC3 cells across treatment groups. n = 4 per group. **G–H** Transwell migration assays showing microglial motility under different lactylation states. n = 9 per group. **I–J** EdU incorporation assays quantifying microglial proliferation in BV2 and HMC3 cells. n = 3 per group. Data are presented as mean ± SD. **p*<0.05, ***p*<0.01, ****p*<0.001.

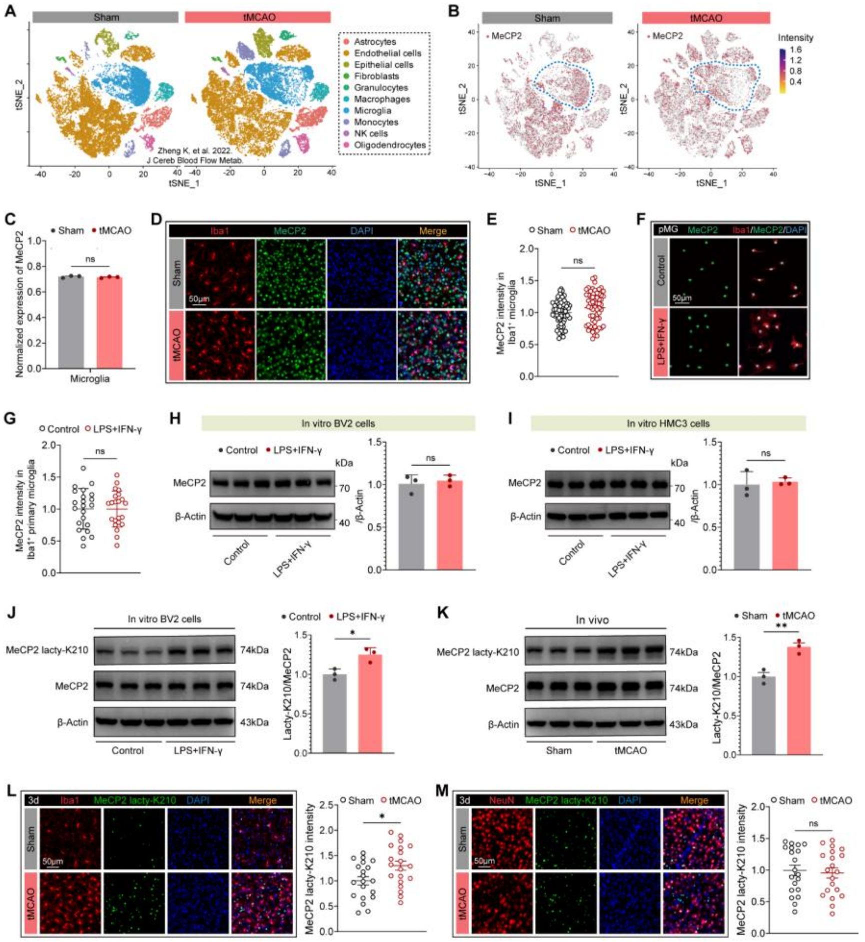

Fig. S7 MeCP2 levels remain unchanged in microglia, while its lactylation is enhanced under inflammatory conditions. A-B t-SNE plots from single-cell RNA sequencing datasets (Zheng K, et al., 2022) showing brain cell populations in Sham and tMCAO mice and spatial distribution of MeCP2 expression. **C** Quantification of *Mecp2* mRNA expression in microglia from Sham and tMCAO mice shows no significant change. **D–E** Immunofluorescence staining of Iba1^{+} microglia for MeCP2 in brain sections from sham and tMCAO mice, and quantification of MeCP2 signal intensity. n > 30 cells per group. **F–G** Immunofluorescence and quantification of MeCP2 intensity in primary microglia stimulated with LPS+IFN-γ. n > 20 cells per group. **H–I** Western blot analysis of MeCP2 protein levels in BV2 and HMC3 cells with or without inflammatory stimulation. n = 3 per group. **J** Western blot analysis of MeCP2 K210 lactylation levels in BV2 cells under control or LPS+IFN-γ stimulation. n = 3 per group. **K** Western blot analysis of MeCP2 K210 lactylation levels in brain tissue from sham and tMCAO mice. n = 3 per group. **L** Immunofluorescence staining of brain sections at 3 days post-tMCAO for Iba1 and MeCP2 lacty-K210, with quantification of MeCP2 lacty-K210 fluorescence intensity in Iba1^{+} microglia. n ≥ 20 cells per group. **M** Immunofluorescence staining of brain sections at 3 days post-tMCAO for NeuN and MeCP2 lacty-K210, with quantification of MeCP2 lacty-K210 fluorescence intensity in NeuN^{+} neurons. n ≥ 20 cells per group. Data from animal experiments are means ± SEM; those from cell line experiments are means ± SD, ns = not significant, $*p < 0.05$, $**p < 0.01$.

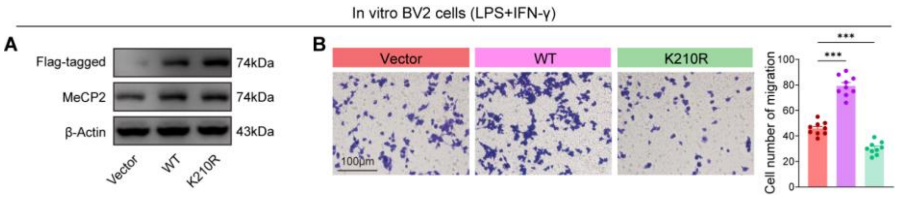

Fig. S8 MeCP2 K210R attenuates microglial migration. A Western blot analysis of Flag-tagged MeCP2 expression in BV2 cells transfected with vector, wild-type (WT), or K210R MeCP2 constructs. **B** Representative transwell migration images and quantification of migrated BV2 cells across vector, WT, and K210R groups. n = 9 per group. Data are shown as mean ± SD. ***p<0.001.

A

Identified proteins after MeCP2 Immunoprecipitation

Protein accession	Protein description	Gene name	Peptides	Razor + unique peptides	Unique peptides	Intensity
B2RWS6	Histone acetyltransferase p300	*Ep300*	3	3	3	1682.04
P70288	Histone deacetylase 2	*Hdac2*	5	2	2	1604.04
O09106	Histone deacetylase 1	*Hdac1*	6	6	6	6398.15
Q8BKJ9	NAD-dependent protein deacetylase sirtuin-7	*Sirt7*	3	3	3	8376.22

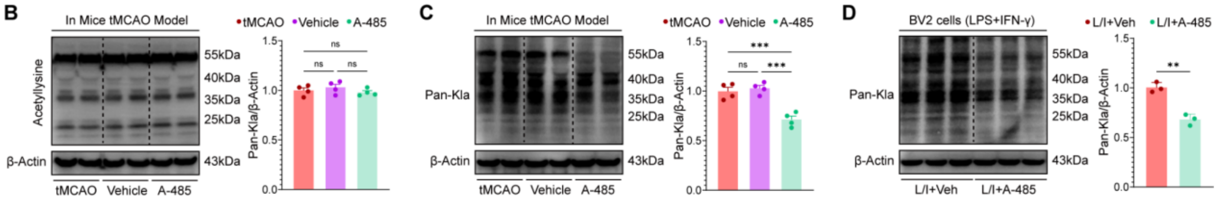

Fig. S9 Identification of p300 as a MeCP2-interacting protein and pharmacological inhibition of p300 suppresses lactylation. A Table summarizing proteins identified by mass spectrometry after MeCP2 immunoprecipitation, including histone acetyltransferase p300 (*Ep300*), histone deacetylases (*Hdac1*, *Hdac2*), and NAD-dependent deacetylase sirtuin-7 (*Sirt7*). **B** Western blot analysis of total lysine acetylation (Acetyllysine) in peri-infarct tissue from tMCAO mice treated with vehicle or A-485. n = 4 per group. **C** Western blot of global protein lactylation (Pan-Kla) in the tMCAO brain tissue reveals a significant reduction in the A-485-treated group. n = 4 per group. **D** Western blot of Pan-Kla in BV2 cells treated with LPS+IFN-γ and vehicle or A-485, showing reduced lactylation upon p300 inhibition. n = 3 per group. Data from animal experiments are means ± SEM; those from cell line experiments are means ± SD, ns = not significant, **p<0.01, ***p<0.001.

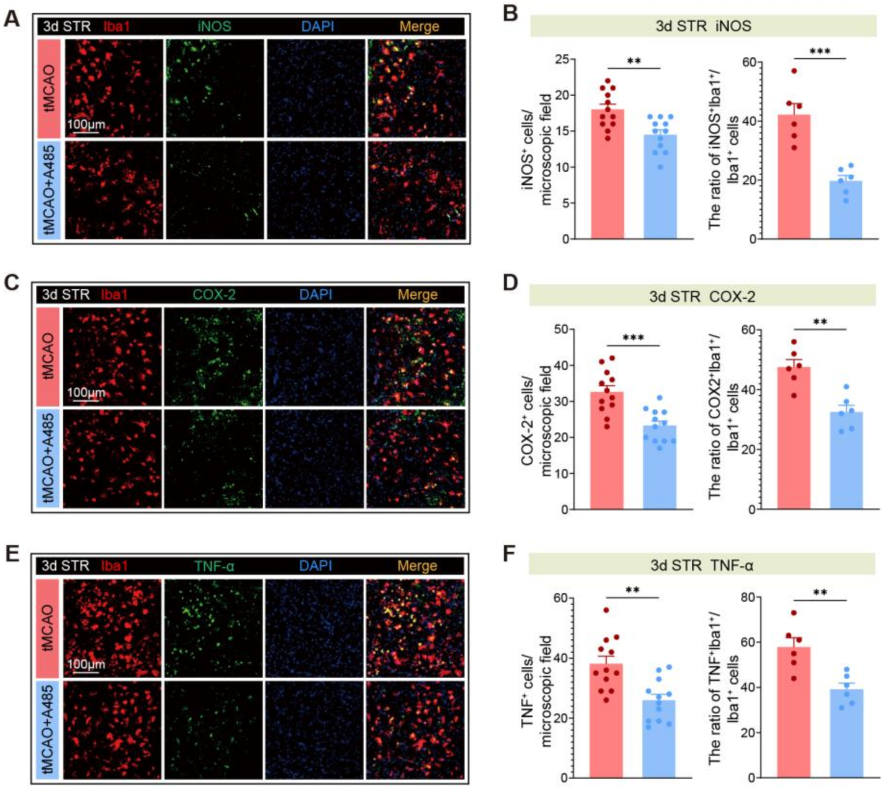

Fig. S10 P300 inhibition reduces iNOS, COX-2, and TNF-α expression in microglia of the ischemic striatum. A, C, E Representative immunofluorescence images in the peri-infarct striatum showing Iba1 co-stained with iNOS (A), COX-2 (C), and TNF-α (E) in the striatum at 3 days post-tMCAO, with or without A-485 treatment. **B, D, F** Quantification of total iNOS$^+$, COX-2$^+$, and TNF-α$^+$ cells per field and their proportions among Iba1$^+$ microglia. n = 12 fields per group from 6 mice. Data are presented as mean ± SEM. **p<0.01, ***p<0.001.

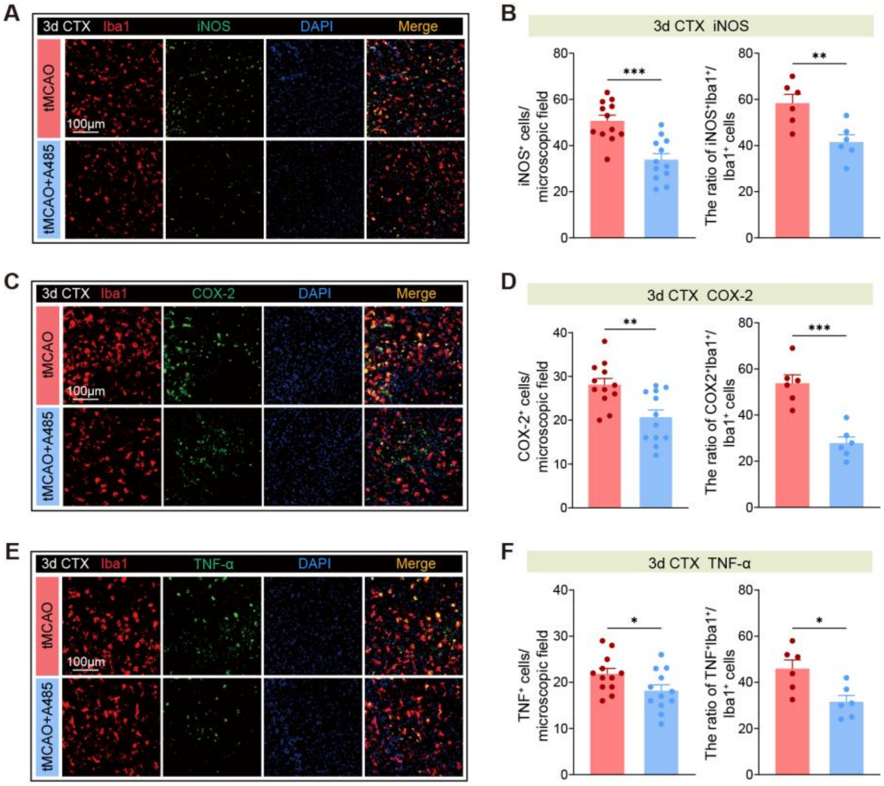

Fig. S11 A-485 attenuates iNOS, COX-2, and TNF-α expression in microglia of the ischemic cortex. A, C, E Representative immunofluorescence images in the peri-infarct cortex showing Iba1 co-stained with iNOS (A), COX-2 (C), and TNF-α (E) in the cortex at 3 days post-tMCAO, with or without A-485 treatment. **B, D, F** Quantification of total $iNOS^+$, $COX\text{-}2^+$, and $TNF\text{-}\alpha^+$ cells per field and their proportions among $Iba1^+$ microglia. n = 12 fields per group from 6 mice. Data are presented as mean ± SEM. **p*<0.05, ***p*<0.01, ****p*<0.001.

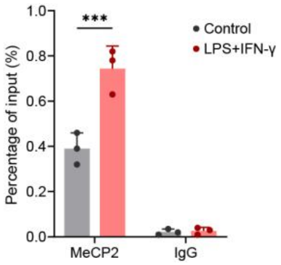

Fig. S12 MeCP2 directly binds to the *Hk2* promoter in microglia. Chromatin immunoprecipitation (ChIP)-qPCR analysis of MeCP2 binding to the *Hk2* promoter in BV2 microglial cells under control conditions or stimulated with LPS+IFN-γ. IgG served as a negative control. Data are presented as mean ± SD. n = 3 per group. ***p*<0.001.

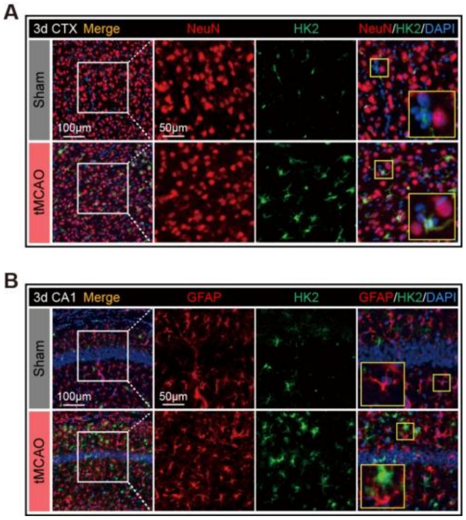

Fig. S13 HK2 is selectively upregulated in microglia following tMCAO. A Representative image in the peri-infarct cortex showing HK2 does not co-localize with NeuN+ neurons at 3 days post-tMCAO. **B** Representative images of the hippocampal CA1 region demonstrating the absence of HK2 expression in GFAP+ astrocytes.

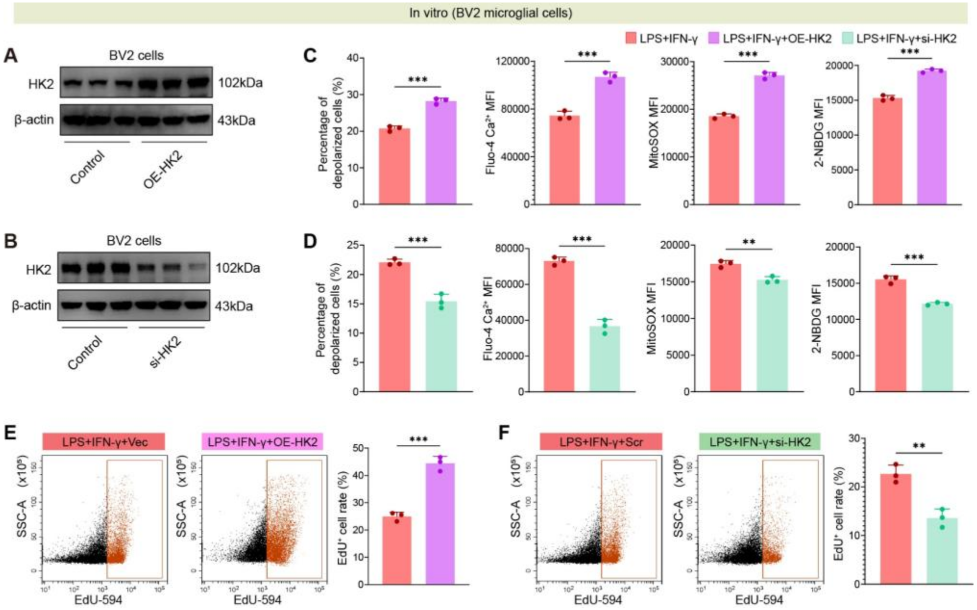

Fig. S14 HK2 regulates mitochondrial homeostasis, glycolysis, and cell proliferation in BV2 microglia. A–B Western blots confirming successful HK2 overexpression (OE-HK2) and knockdown (si-HK2) in BV2 cells. **C–D** Quantification of mitochondrial depolarization, calcium influx (Fluo-4), ROS levels (MitoSOX), and glucose uptake (2-NBDG) following HK2 overexpression (C) or knockdown (D) under LPS+IFN-γ stimulation. **E–F** Flow cytometry analysis of EdU incorporation shows enhanced proliferation in OE-HK2 cells and decreased proliferation in si-HK2–treated cells. n = 3 per group. Data are presented as mean ± SD. $^{**}p < 0.01$, $^{***}p < 0.001$.

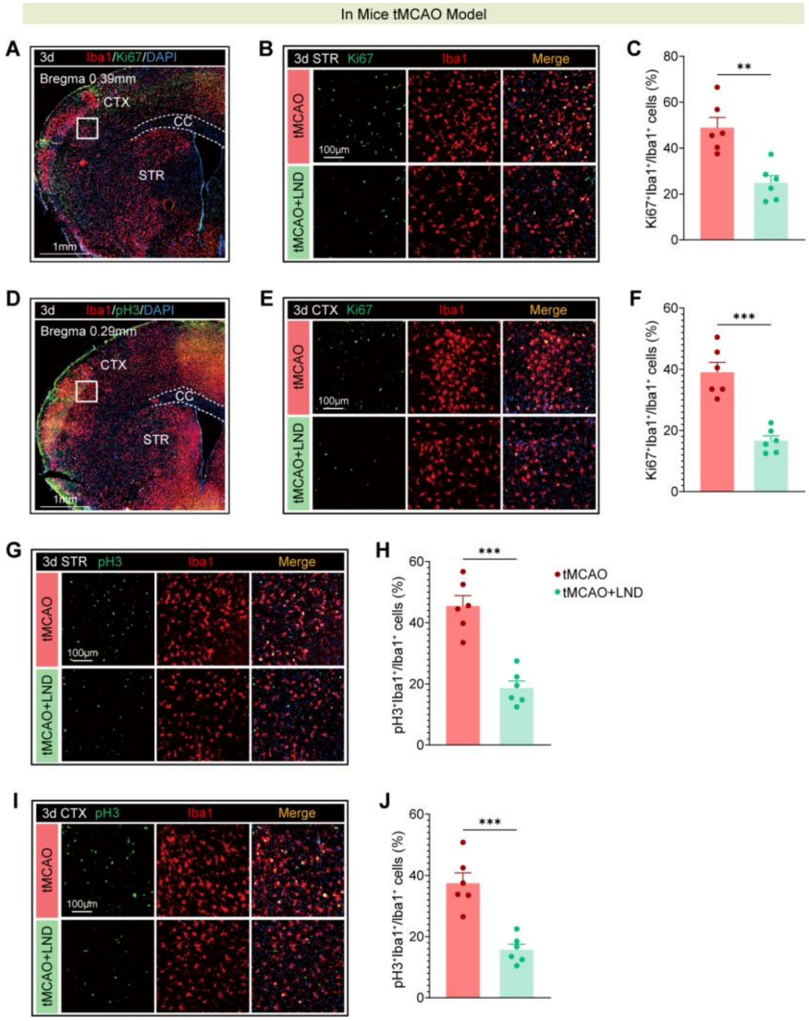

Fig. S15 Inhibition of HK2 by lonidamine reduces microglial proliferation in the striatum and cortex after ischemic stroke. A, D Representative coronal brain sections showing immunofluorescence staining of Iba1, Ki67 or phospho-Histone H3 (pH3) at 3 days post-tMCAO, with or without lonidamine (LND) treatment. **B, E** High-magnification images of Iba1 and Ki67 in the peri-infarct striatum (STR) and cortex (CTX), respectively. **C, F** Quantification of percentage of $Ki67^{+}Iba1^{+}$ cells in STR and CTX. LND treatment significantly reduced microglial proliferation. **G, I** Representative images of Iba1 and pH3 co-labeling in STR and CTX. **H, J** Quantification of percentage of $pH3^{+}Iba1^{+}$ cells showing reduced mitotic activity in LND-treated mice. n = 6 mice/group. Data are presented as mean ± SEM. *p<0.05, **p<0.01, ***p<0.001.

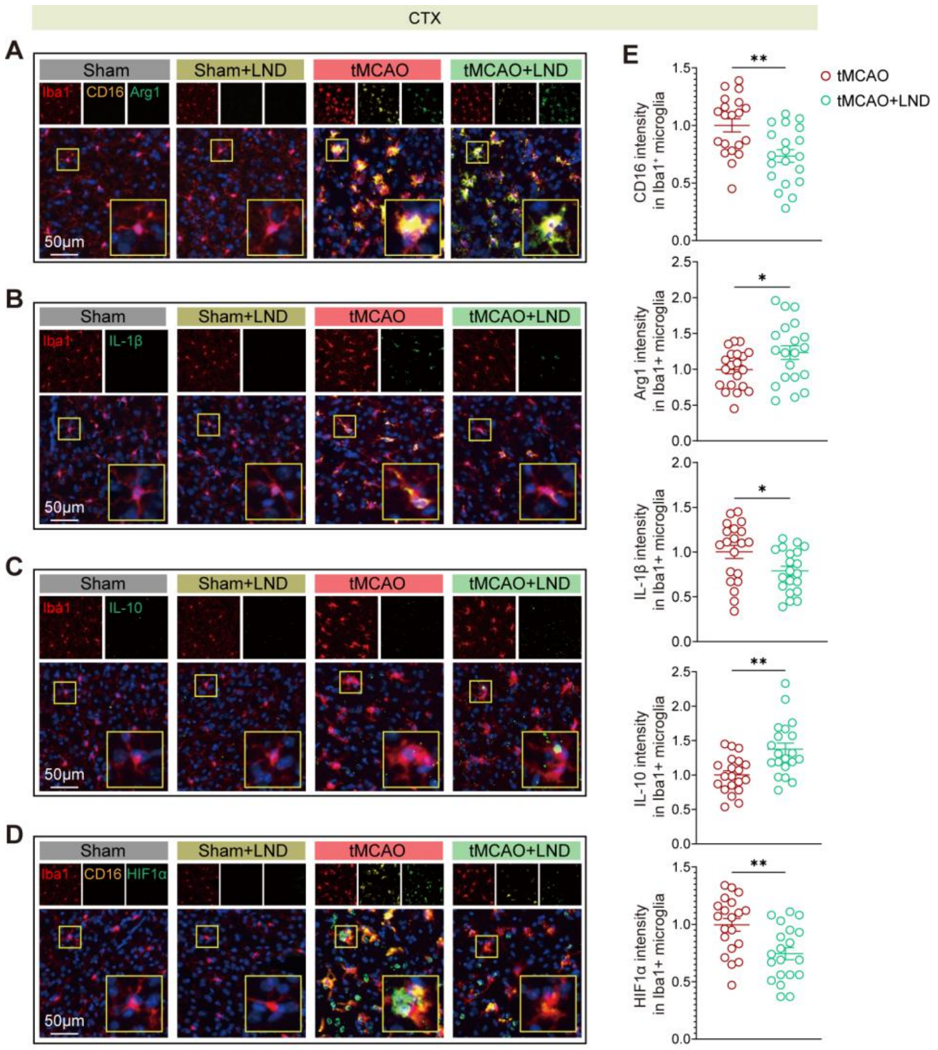

Fig. S16 Inhibition of HK2 by lonidamine reprograms microglial phenotype, reduces neuroinflammation, and modulates the ischemic microenvironment in the cortex at day 3 post-tMCAO. A–D Representative immunofluorescence images of cortical sections co-stained for Iba1 and either CD16 and Arg1 (A), IL-1β (B), IL-10 (C), or HIF-1α (D), showing differential expression patterns among Sham, Sham+LND, tMCAO, and tMCAO+LND groups. **E** Quantification of fluorescence intensity of CD16, Arg1, IL-1β, IL-10, and HIF-1α in $Iba1^{+}$ microglia. LND treatment significantly decreased pro-inflammatory CD16, IL-1β, and HIF-1α expression, while increasing anti-inflammatory Arg1 and IL-10 intensity. n ≥ 20 cells per group. Data are presented as mean ± SEM. *$p < 0.05$, **$p < 0.01$.
