# Supplementary material for: Flipping the Switch: MeCP2‐Mediated Lactylation Rewires Microglial Metabolism and Inflammation via the HK2/mTOR Axis in Poststroke Neuroinflammation
Source: Adv Sci (Weinh). 2025 Dec 22;13(12):e13400. doi: 10.1002/advs.202513400 (PMC12948279; doi:10.1002/advs.202513400)

Original Western Blots

These are all the WB band images from this article. We have provided the uncropped full membranes.

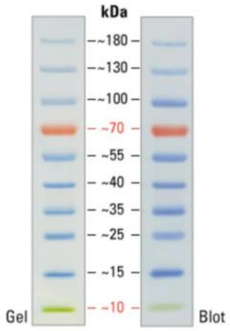

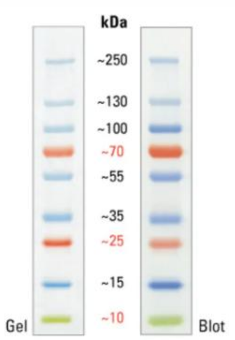

We used two protein markers in our WB experiment. One is suitable for high molecular weight proteins (Thermo Fisher Scientific, catalog number 26619), and the other is for medium to low molecular weight proteins (catalog number 26616, 10-180 kDa). In all WB bands for high molecular weight proteins, we used marker 26619 (10-250 kDa).

The red box highlights our target band, with β-actin used as the internal control.

Figure 1

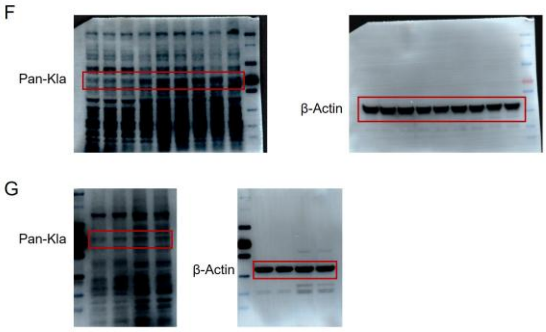

Figure 2

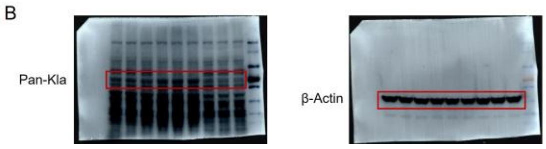

Figure 3

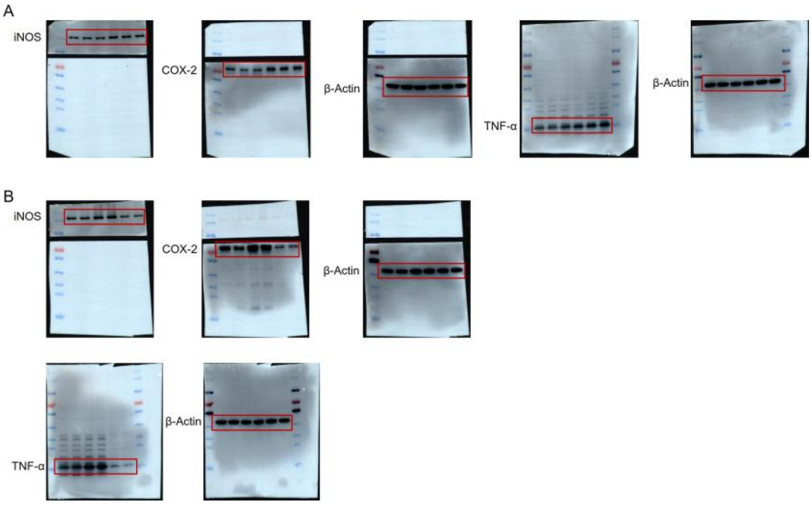

Figure 4

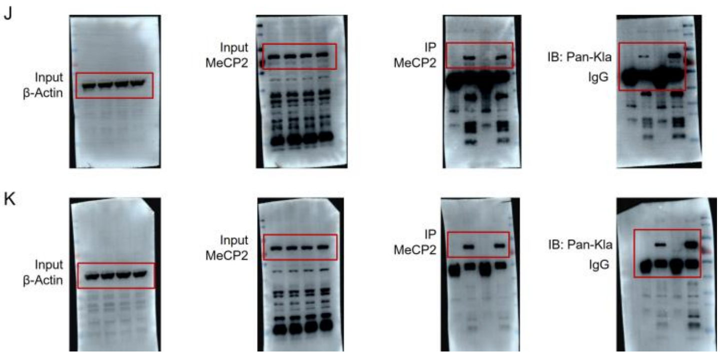

Figure 5

H

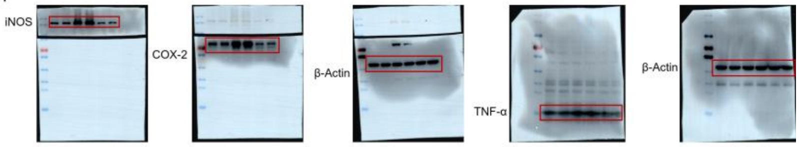

J

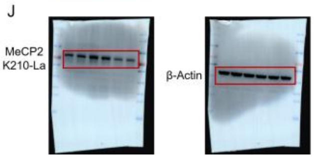

Figure 6

B

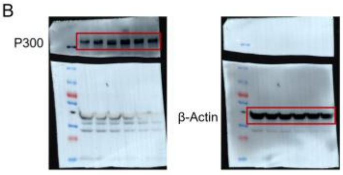

C

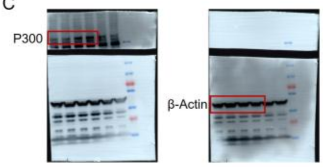

F

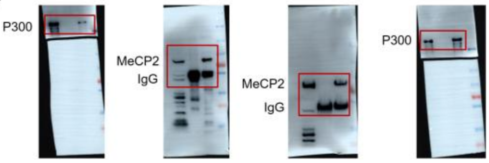

G

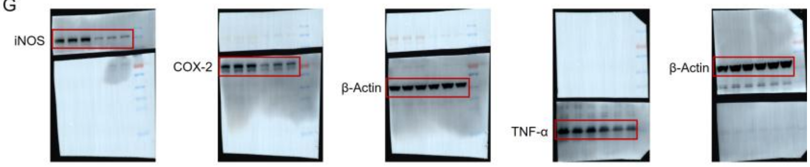

H

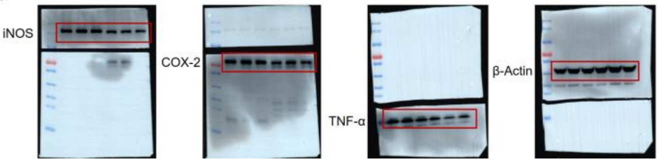

Figure 8

C

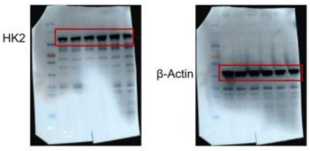

H

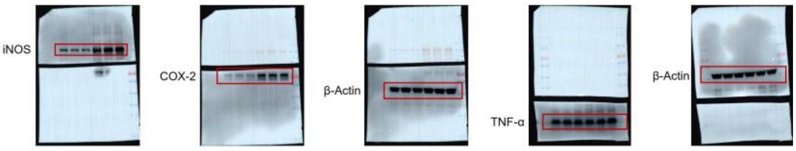

L

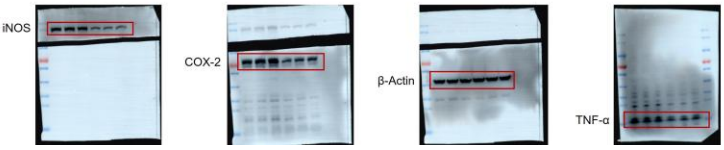

Figure 9

B

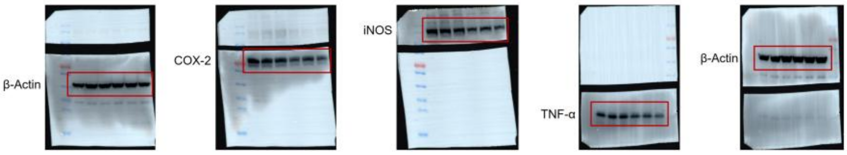

C

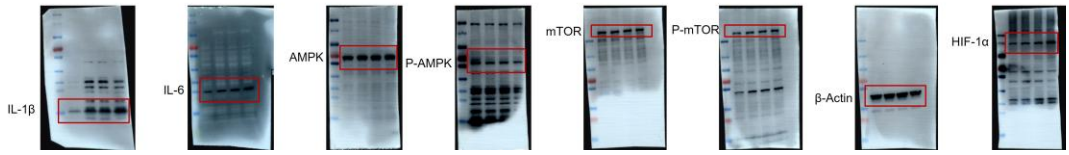

Figure 9

E

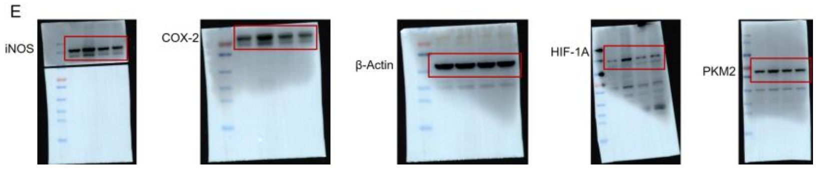

Figure 9

F

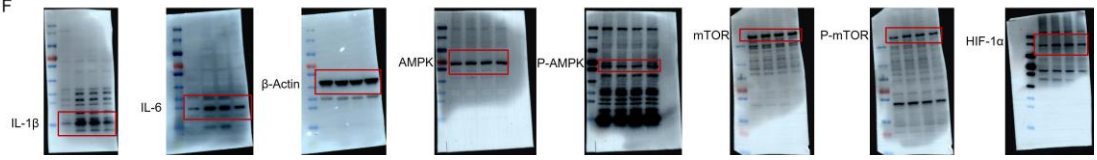

Figure 9

H

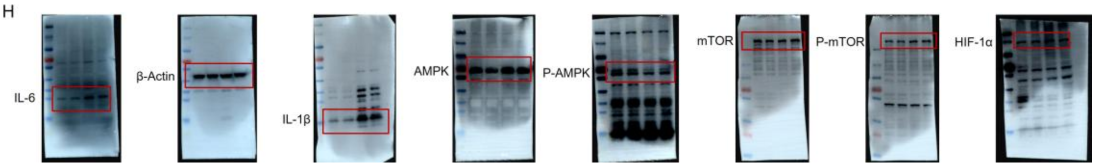

Figure S2

A

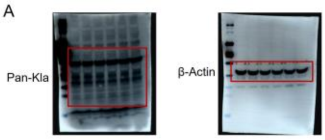

B

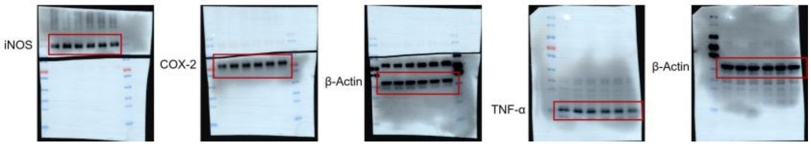

Figure S5

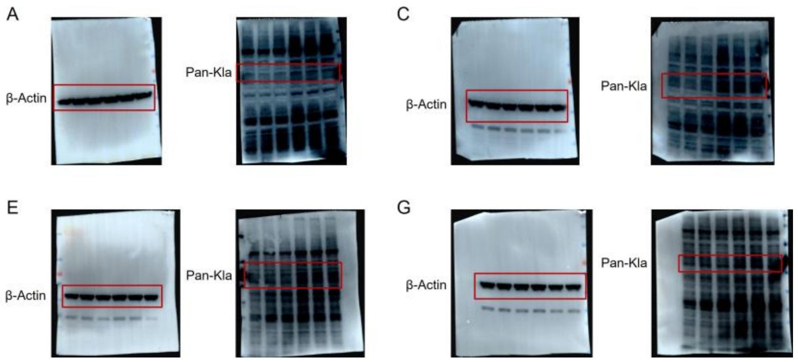

Figure S6

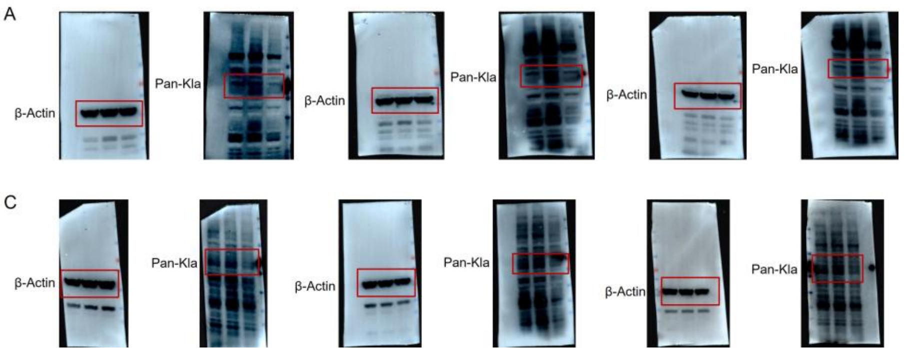

Figure S7

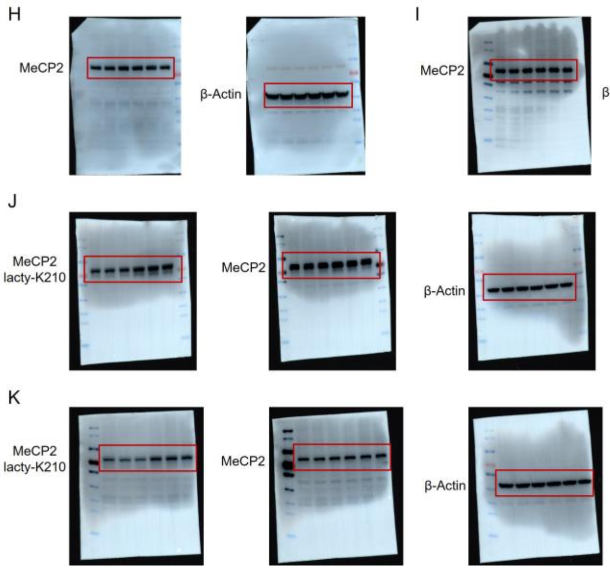

Figure S8

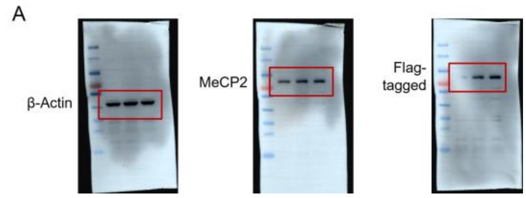

Figure S9

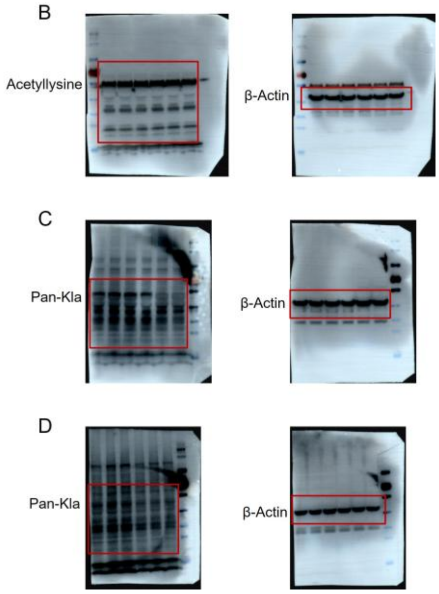

Figure S14

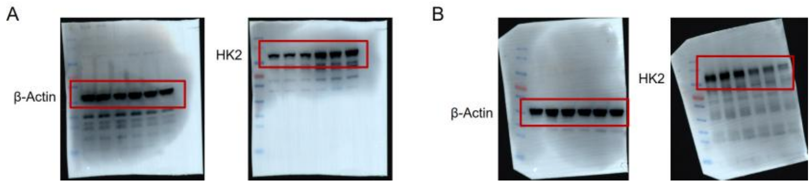

Supplement: Supplementary file 3 — Supporting Information [file ADVS-13-e13400-s002.zip › advs73283-sup-0003-Data/Original Western Blots.pdf]
